# Energy use and its contributors in hotel buildings: A systematic review and meta-analysis

**DOI:** 10.1371/journal.pone.0309745

**Published:** 2024-10-24

**Authors:** Rodrigo Schons Arenhart, Tailon Martins, Renan Mitsuo Ueda, Adriano Mendonça Souza, Roselaine Ruviaro Zanini

**Affiliations:** 1 Production and Systems Department, Federal University of Santa Maria, Santa Maria, Rio Grande do Sul, Brazil; 2 Production Engineering Department, Federal University of Mato Grosso do Sul, Nova Andradina, Mato Grosso do Sul, Brazil; 3 Department of Statistics, Federal University of Santa Maria, Santa Maria, Rio Grande do Sul, Brazil; Public Library of Science, UNITED KINGDOM OF GREAT BRITAIN AND NORTHERN IRELAND

## Abstract

Energy use is the major source of carbon emissions in hotel buildings. Past studies presented contributors to energy use, most related to hotels’ physical and economics characteristics. In search of effective variables affecting energy use in hotels, this systematic review and meta-analysis aims to synthesize empirical evidence. A descriptive picture of 28 previous studies, the arguments for the direction of effects in each variable, and a quantitative synthesis of the mean effect sizes were presented. Among 18 selected contributors from past studies, 15 were statistically significant (0.05 level). The analyses also revealed that the operationalization of the energy variable is important in evaluating the relationship with a contributor. Studies considering Energy Use Intensity (EUI) indicators presented weaker correlations with gross floor area (GFA) and number of guestrooms than those considering energy raw data, for example. The occupancy rate resulted in a non-significant outcome, but this result could be related to differences among the hotels categories, as identified in the subgroup and meta-regression analyses. Future research could help develop and investigate theories to sustain or deny the relationships found here, in addition to the assessment of the outcomes in other regions, bringing more variables related to sustainable management.

## Introduction

The energetic efficiency of hospitality operations has emerged as a central concern in recent studies [1–5], reflecting a greater awareness of the organizational impacts associated with energy use in the sector. Green products have also experienced rising consumption in the last few years [6] while sustainable practices in hotels are contributing to their development and generating value for the owners and society [7]. This research focuses on the overall energy use by hotels, which is predominantly grid electricity.

Among worldwide guests, 81% believe that sustainable travel is important [8], with a major concern among young generations [9]. Even so, the hotel industry accounts for a significant amount of the overall tourism resource consumption [10], with food, water and energy being the main impacted resources [11]. Tourism emissions trends are expected to triple by 2050 [12], while the Intergovernmental Panel on Climate Change (IPCC) estimated that global surface temperature had reached 1.1°C above the pre-industrial temperature between 2010 and 2019 [13]. To align growth expectations and climate issues, a decarbonization rate between 8 and 10% per year is estimated, which could help maintain a level of 1.5°C above pre-industrial temperature [14].

The tourism industry is a multidimensional system in which the dimensions interrelate and have strong interdependencies requiring interdisciplinary research [15]. In Europe, to assess the progress of ecological transitions, indicators related to production and consumption, waste management, secondary raw materials, and competitiveness and innovation have been developed in addition to environmental performance indicators [11]. Most hotel sustainable research recommends considering environmental management responsibility in general and resource management in particular [16]. It is well known that electricity consumption is the dominant source of energy in the hotel industry and that energy use is the major source of carbon emissions in this sector [10, 17–19]. With this, a pressing need by the decision-makers of the hotel industry to reduce their energy consumption is becoming relevant [20].

Some typical variables used in the last 10 years of studies to analyze energy use in hotels include the year of construction, gross floor area (GFA), occupancy rate, number of guest-nights, number of guestrooms and star rating [2, 10, 21, 22]. Regarding Energy Use Intensity indicators (EUI), most of the papers reported energy use intensity based on GFA [3, 18], while some reported it based on guest-nights [1, 10, 23] and the number of guestrooms [24]. These contributors to energy use are related to physical building characteristics, and less or no attention was given to those contributors related to economic, environmental, or social outcomes. This systematic review and meta-analysis aim to identify contributors to energy use in hotel buildings, provide a descriptive picture of the research stream, and synthesize the theoretical foundation in each variable, and determine the magnitude of the mean effect size on hotels’ energy use.

The research fulfils the lack of systematic reviews and meta-analyses considering energy use and its contributors in hotel buildings and the lack of theoretical frameworks on these relationships. The identification of robust empirical findings in the meta-analyses along with the subgroup analysis and meta-analytic regression, understanding the covariates affecting the variables outcomes, can serve as a basis for further research on energy efficiency in hotels. From a sustainable view, the study presents variables related to business sustainability that affect energy use in hotel buildings and can help practitioners, academics, and legislators build measures to improve energy efficiency and mitigate costs, which can help reduce the sector’s carbon footprint.

## Literature review

In tourism industry, empirical studies have conducted analyses indicating contributors influencing energy use in hotel buildings. For each variable included in the meta-analyses, the most relevant outcomes from previous research are given to estimate the strength and direction of effects. The theories proposed by the selected studies will also be discussed to understand and assess energy use and their contribution to the research field. The significant contributors to energy use in hotels are diverse, and regularly are dependent on physical and operational parameters, such as hotel size, GFA, construction year, source of energy, occupancy rate and star rating [21]. Cabello Eras et al. [25] affirm that, in addition to facilities and operational characteristics, energy use in hotels varies with the geographic location, mainly due to the weather and climate. Papers also revealed the contribution of social and environmental indicators like the number of employees [10, 19, 21, 24, 26, 27] and the outdoor temperature [26–28].

Previous meta-analyses covered energy consumption and hotel chains but have not applied this analysis in conjunction. Campagna and Fiorito [29] mapped the impacts of climate change on building energy consumption (mainly residential and office buildings), indicating that global warming leads to a decrease in heating consumption, an increase in cooling consumption, and a growth in total energy consumption. Hu et al. [30] verified the effectiveness of passive cooling strategies (PCSs) in residential buildings for energy savings, which reached 29% for the use of various passive strategies (e.g., natural ventilation, building orientation, thermal cooling).

In hospitality research, Gao et al. [31] aimed to determine the effect sizes of the relationship between consumers’ perceptions (e.g., internalized and perceptions of the firm) and their green behavior (e.g., word-of-mouth intentions, willingness to pay), identifying a strong positive association. Dimara et al. [32] conducted a meta-analysis reporting that 53% of guests are willing to financially support green hotels, while Yang et al. [33] encountered a positive effect in the relationship between electronic word of mouth (eWOM) and hotel performance. These meta-analyses are relevant to infer that there is a growing importance in research into the use of resources in commercial buildings and that in the hospitality sector, there is a need to encourage more sustainability practices. However, our review offers further insight into the analysis of energy use and its contributors in hotel buildings from the viewpoint of previous empirical studies that have not yet been considered.

Variables selected to meta-analyses, and assessed in previous studies, were divided into four dimensions: building characteristics, economics, social and environmental. In building characteristics, floor area (GFA) and number of guestrooms were the most used variables to estimate the relationship with energy use in hotels, due to the simple collection method and representativeness of the outcomes. Floor area was correlated with energy use in several past studies, most of which had a strongly significant positive relationship when using raw energy data [10, 18, 19, 22, 24, 26, 27, 34–41]. The number of guestrooms has also a considerable number of studies involving energy use and has presented moderate positive behavior in most reports [2, 10, 18, 19, 22, 24, 27, 34, 38, 40, 42]. Studies using normalized indicators, like EUI, for energy data, resulted in weaker correlations for both variables [18, 24, 26, 27, 40].

The other building characteristics indicators appear in fewer studies. The hotel category is being analyzed since 2012 and seems to have a positive relationship with energy use, which could be viewed as more energy demand in upscale hotels [3, 19, 21, 27, 34]. The hotel construction year and the variable related to the building age represent a common characteristic, where the first is expected to have a negative relationship with energy use and the second a positive one, but the results in the scientific literature presented mixed outcomes [18, 21, 24, 26, 27, 34, 41, 42]. The number of floors presented, as expected due to construction characteristics, a positive relationship with energy use in two studies [10, 26], it was neutral in one [34] and negative in another [19], similar to the outcomes from studies reporting the year after the last retrofit in a hotel building, that presented two positive correlations with energy use [21, 26] and one negative [19]. The area of guestrooms presented a moderately positive relationship with energy use in the studies where it was considered [2, 21, 26].

In the economic dimension, the occupancy contributor does not have a considerable effect on hotel energy consumption in some cases [2, 34, 37, 40, 42, 43], while in others the variable has a positive effect [19, 21, 26, 27, 35, 36, 44] and even a negative one [10, 18, 23, 24]. This variable suffers from a wide level of variation in its results among the published studies, which should be investigated further. Regarding guest-nights, the relationship with energy use is more robust with all the sampled studies reporting positive outcomes [2, 10, 19, 24, 26, 40, 41], supporting the idea that energy use is dependent on the number of guest-nights sold in a hotel. The number of guests presented a positive and strong relationship with energy use in three studies [10, 27, 45], but divergent results in the other two papers [38, 46], which could point out some differences in the studies, like the operationalization of the dependent variable, geographical location, or guest demand curve. In studies considering food covers [2, 19, 41] and the room revenue of hotels [19, 21, 27, 47], both variables presented positive relationships with hotel energy use.

The number of employees [10, 19, 21, 24, 26, 27] and the employees’ density [10, 26, 27, 45] were the only variables, found in the systematic review, regarding the social dimension that were analyzed in more than one study. Both showed positive relationships with energy use, but the former was stronger than the latter, suggesting that the overall number of employees has a greater influence on energy consumption than the number of employees per shift. Concerning environmental aspects, even with fewer studies, all the analyzed variables reported strong and positive associations with energy use in hotels, meaning that with higher outdoor temperatures [26–28], more water use [3, 19, 47] and carbon emissions [24, 46, 47], hotels would consume more energy.

Regarding theories and formulations about energy efficiency in hotels, only three were logically developed as frameworks in the review corpus. Yoon et al. [3] used the water-energy nexus (WEN), which is a novel approach to understanding the interactions between water and energy systems. The authors sustain that addressing resource issues in hotels through the WEN framework would provide additional and more comprehensive insights to improve resource management. Bhochhibhoya et al. [1] applied life cycle thinking to understand the performance of hotel buildings. This framework consists of an impact’s evaluation of the entire cycle of buildings associated with the extraction, manufacturing and transportation of construction materials, the impact of building construction, operation, maintenance, and the end-of-life stages [1, 48]. While Xin et al. [42] established energy consumption quotas for luxury hotels in Hainan Province. The authors affirmed that the quota defines a reasonable level for special buildings’ energy use in a period by identifying the upper limit of building energy use intensity and could be used as an indicator of energy efficiency. The proposed frameworks could not be seen as theories linking the possible effects of the variables contributing to energy use but rather as ways of achieving a more efficient use of resources in hotel chains. However, the conceptual models are limited to explain the outcomes of the empirical studies.

There is a complex relationship between hotel revenue, occupancy rates, and energy consumption. Most previous studies indicate that electricity is the primary energy source, with air conditioning and food services being major consumers. Factors like the luxury level of hotels and the number of services offered significantly impacted energy use. Additionally, there is a strong correlation between water and energy consumption, particularly in hotels with water heating systems. Factors such as the total floor area, worker density, and the type of fuel used influence both energy use and carbon emissions.

Energy management practices, such as the use of benchmarks and specific indicators, are essential for monitoring and reducing consumption, cases of replacing fossil fuels with cleaner options and implementing retrofit technologies are effective strategies for improving energy efficiency and sustainability in hotels. The sector is benefiting from the adoption of energy efficiency measures and conscious management of resources, which is crucial to reducing the environmental impact of the hotel industry, maintaining a good brand image and achieving good organizational results. Tested machine learning methods are also helpful when selecting the most appropriate and efficient equipment at the hotel design stage with data inputs from the operation [49].

## Materials and methods

This review follows the Preferred Reporting Items for Systematic Reviews and Meta-Analysis Statement updated in 2021 (PRISMA 2020), which aims to help reviewers report why the review was done, what was done, and what was found, being a useful tool when planning and conducting systematic reviews to ensure the report of all recommended information [50]. In accordance with Shamseer et al. [51], seeking to reduce bias, increase transparency, allow scrutiny, reduce any unintended duplication, and improve reliability, the protocol for this systematic review was registered on the Open Science Framework platform under the DOI code: https://doi.org/10.31219/osf.io/yuw8e. The PRISMA 2020 Checklist is presented in S1 Checklist.

### Eligibility criteria and search strategies

The studies were selected following the eligibility criteria based on other meta-analyses published in business and hospitality literature and the PRISMA-P guidelines for preparing review protocols [50, 52–55]. To be included, the studies must use quantitative data and investigate the relationship between at least one independent variable (floor area, occupancy rate, number of rooms, carbon intensity, water use, among others) and the energy use in hotel buildings. These variables were always related to hotels or hotel chains of all levels (star rating), open or closed companies. The included studies have assessed the relationships among the variables using univariate or multivariate statistics techniques (i.e., correlations and/or regressions). The covariates were dummy and continuous data, but energy use was always continuous.

When papers used the same sample as another study, the more representative (precise) one was maintained in the analysis as the dataset, and the other(s) were used if they presented different variables from the first study. When the studies used the same data and variables, without any difference in the correlations among them, the first published was used in meta-analysis. If the same study presents more than two correlation coefficients in nearby geographic areas, e.g., the same country, then only the two more representative samples were incorporated in the meta-analysis. In case different operationalization of the same variable in a study, i.e., reports with the use of raw energy data and/or the use of normalized energy data, the two correlation outcomes were considered only if effect sizes appeared more than ten times, otherwise, raw energy data was used. The eligibility criteria were applied by three independent researchers who had read the abstracts collected from the two search strings developed. The search was conducted in the Scopus and Web of Science (WoS) databases, which are well-established in the subject considering their vast collection, representativeness, and relevant publishers indexed, e.g., Elsevier, IEEE, MDPI, and Springer [56, 57].

The snowball sampling technique, applied by Feil et al. [58] and Schmid and Morschett [55], was also employed. This sampling procedure involves the manual reading of the citations and references of the selected papers, aiming to discover more relevant papers that were not presented in the systematic database search. A search for previous reviews was done, but neither a systematic review nor a meta-analysis involving energy use and its contributors in hotels was found until the day before the protocol registration, October 26, 2023.

**Fig 1:**
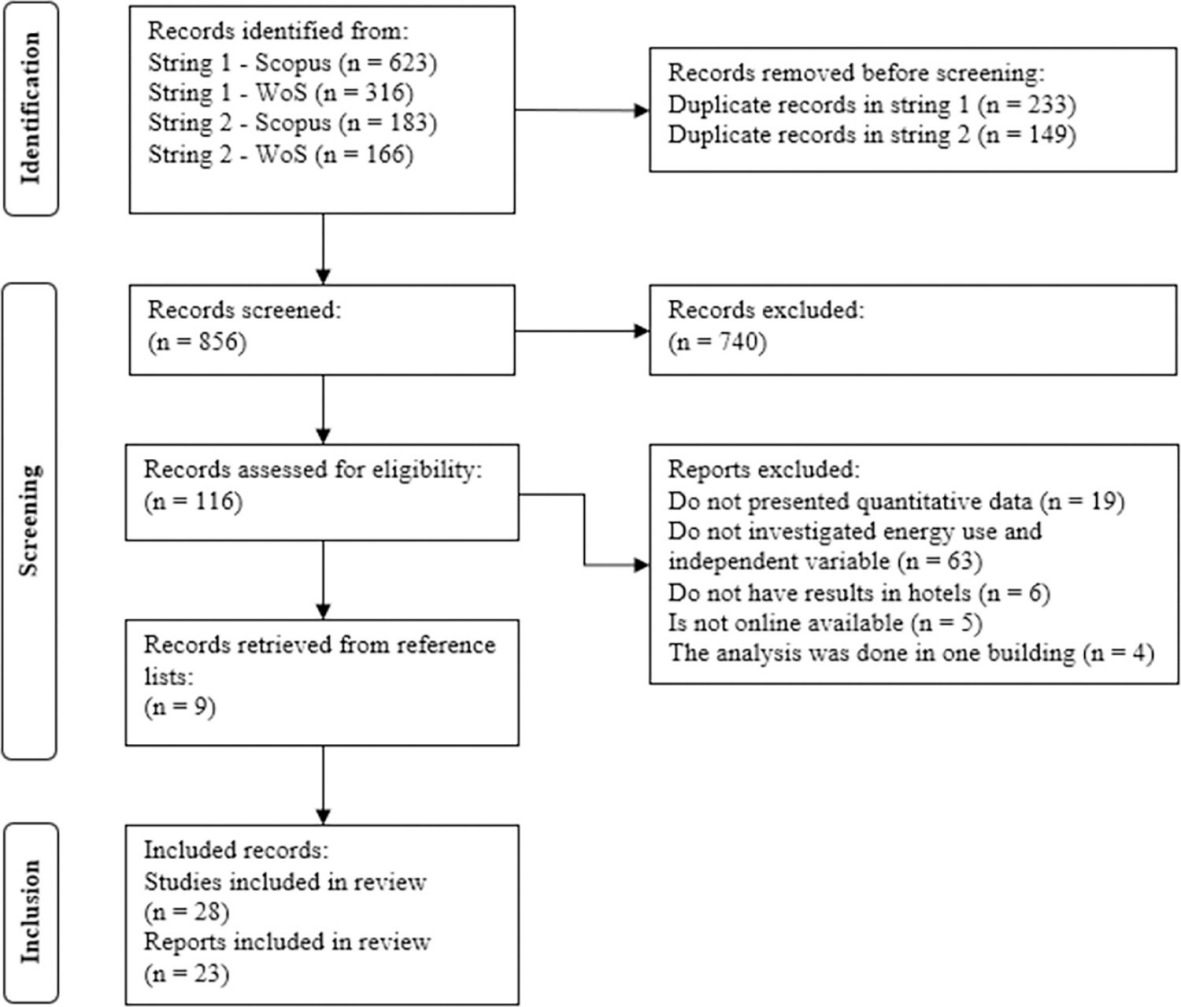
PRISMA flowchart for study selection.

Two search strings were applied in the title, abstract, and keywords fields in the two databases with the use of the following words: i) String 1 "energy" OR "energy use" OR "energy consumption" OR "energy performance" OR "resource consumption" AND "hotel*" AND "sustai*"; ii) String 2 "energy" OR "energy use" OR "energy consumption" OR "energy performance" OR "resource consumption" AND “hotel*” AND “correlat*” OR “regression*”. In the report characteristics, there were no restrictions about the publication year or data collection. Conference papers, short communications, dissertations, thesis, or technical reports also be considered due to the possibility of reducing publication bias. These procedures follow recent meta-analysis reviews [53, 55] and the method for addressing publication bias proposed by Borenstein et al. [59]. Previous reviews are not considered to be part of the analysis. Any publication language should be considered, but the string keywords will be searched in English. Fig 1 presents the PRISMA flowchart for study selection.

The search strings were applied in the databases between September and October 2023, and the last database check took place on May 30, 2024. In a total of 867 abstracts were evaluated by three researchers, and 751 abstracts were excluded from the analysis, they did not meet the established eligibility criteria because they did not directly analyze energy use and its contributors in hotels. 116 studies were checked, of which 63 did not investigate the relationship between energy use and some independent variable, 19 did not present quantitative data for energy use and/or independent variables, five were not possible to retrieve through online repositories, six presented results outside of hotels, and four presented the analysis in only one building. Thus, 19 papers were selected to compose the research sample through database searches, and nine more were selected using the snowballing technique. The total number of studies incorporated into the review was 28. Among them, four studies repeated two databases and applied the same analysis, so the two latter published, Bhochhibhoya et al. [1] and Xuchao et al. [17], were disregarded for the meta-analysis. Chan and Mak [39] and Chan and Lam [35] used the same collected data from Chan and Lam [36], but the analyses were different among the studies. Kong et al. [34] had used some of the data reported by Xin et al. [42], but only the occupancy rate was repeated, so the rest of the variables were maintained in the meta-analysis. The number of reports from studies maintained in the meta-analysis was 23, the information about each included study can be seen in S1 Table.

### Meta-analytic procedures

The meta-analysis procedures used in this study follow the random effects model, considering the true effect can vary between studies [59]. This is the most appropriate model when sources beyond sampling error could account for heterogeneity in effect sizes [52], which is the case for current data because many contributors to the characteristics analyzed can vary across different samples. This model provides a more accurate estimate of the effect size when the effects present high variability between studies [60], different from a fixed-effect model, which in this case is not a recommendable practice. The between studies variance was estimated with the DerSimonian-Laird estimator because it allows for capturing the real differences in the effects across studies and is the most used method in meta-analyses [55, 59, 61].

This review used meta-analysis on bivariate correlation coefficients, which are natural candidates to be an index of effect magnitude suitable for cumulation across studies [60]. As suggested in specialized literature [59–61] and applied in practice by Schmid and Morschett [55], the correlation coefficients between the variables were transformed using Fischer’s Z scale. For presentation purposes, the values are then transformed back into correlation coefficients. The meta-analyses between the independent variables and energy use in hotels were presented in tables and or forest plots, with their effect sizes, confidence intervals, sample size, study weight, and heterogeneity information. To be included in the meta-analysis, an independent variable had to be investigated in at least three sampled studies. S2 Table shows the independent variables retrieved from the studies included in the meta-analysis and their classification in the organizational aspect of the hotels (environmental, social, economic, or building characteristics).

To estimate if the effect size is consistent among the studies and consider its implications, we used a measure of the weighted squared deviations (Q statistic), the between-studies variance (T^2^), the between-studies standard deviation (T) and the ratio of true heterogeneity to total observed variation (I^2^) [59]. When a selected effect size varies substantially, the study focuses on the variance and applies subgroup analysis or meta-regression, aiming to understand the presented behavior by examining the study-level covariates. The Q-statistic served as a significance test, T^2^ and T pointed to the range of variance between studies, i.e., the quantity of variance, and the I^2^ reveals the proportion of dispersion observed due to the heterogeneity [59]. We applied the tentative benchmarks for I^2^, estimated by Higgins et al. [62] and Baker et al. [63], from which values between 50% and 75% could be placed at a moderate-high level and would require a better understanding of the heterogeneity sources.

Study characteristics that could serve as moderators were collected in the data extraction process and include publication year, approximated geographic location (latitude, longitude, climate zone, and continental area), sample size, period of study, publication year, if the study was supported by theory or not, main method, journal quality, predominant energy source, and hotel category (star rating). The subgroup analysis or the meta-regression were done only if the number of studies was at least equal to ten, because investigating two or more factors in a few studies does not provide enough information to look for subgroup interactions [61]. The statistical results of the heterogeneity analysis were presented.

As with any other meta-analysis, the data may suffer from publication bias due to the heavy emphasis on published research [53]. In addition to the funnel plot in analysis with more than 10 studies, to check for small study effects, the review used statistical tests (i.e., rank correlation test by Begg and Mazumdar [64], regression test by Egger et al. [65]) to provide a quantitative result for possible asymmetries in the funnel plot [61]. We also applied Fail-Safe N tests by Rosenthal [66] and Orwin [67] to estimate the number of studies needed to generate nonsignificant results, verifying the outcomes’ stability. Cumulative meta-analyses, proposed by Borenstein et al. [59], were also done with more than 10 studies, considering the behavior of each added study. The studies were included in different sequences, e.g., the most precise to the least precise, the energy type data (first the raw data than the EUI indicators), and the star level category (from the lower levels to upscale hotels). The publication year was always the second criteria to include the studies. It is important to note that the risk of publication bias exists even with the guidelines taken to reduce it, from the collection of studies to the cumulative meta-analysis and statistical tests to verify asymmetries. All meta-analysis procedures were performed in the R software (RStudio version 2023.03.0+386), with “meta” and “metafor” packages. S1 Appendix presents the meta-analyses coding book.

Aiming to present an estimate of confidence in this meta-analysis, cumulative evidence was applied as an evaluation grade based on objective elements that could represent the risk of bias in individual studies. The elements are the use of primary or secondary data, sample size, the level of support for a theory, the period of analysis, and the amplitude of the study site. The five elements were considered at equal rates of 20% each. Individual studies received grades based on the five assumptions, and the mean value of all studies is the estimated evidence of the review. For data type, primary data received the assessment of “10”, while secondary data was “5”. In sample size, if a study brings a sample of 200 objects or more, it is represented as “10”, and so on for other values (e.g., a sample of 80 is classified as 4.00). Studies based on a theory are classified as “10”, otherwise “5”. Study periods with less than one year are graded “1”, studies between one and two years are “5”, and studies with more than two years are “10”. If a study considers one continent or, more it receives “1”, if the sample includes more than one country “3”, if includes one country “5”, if includes one region of a country “8” and if includes a specific location in a country “10”.

## Results

### Overview of study characteristics

**Fig 2:**
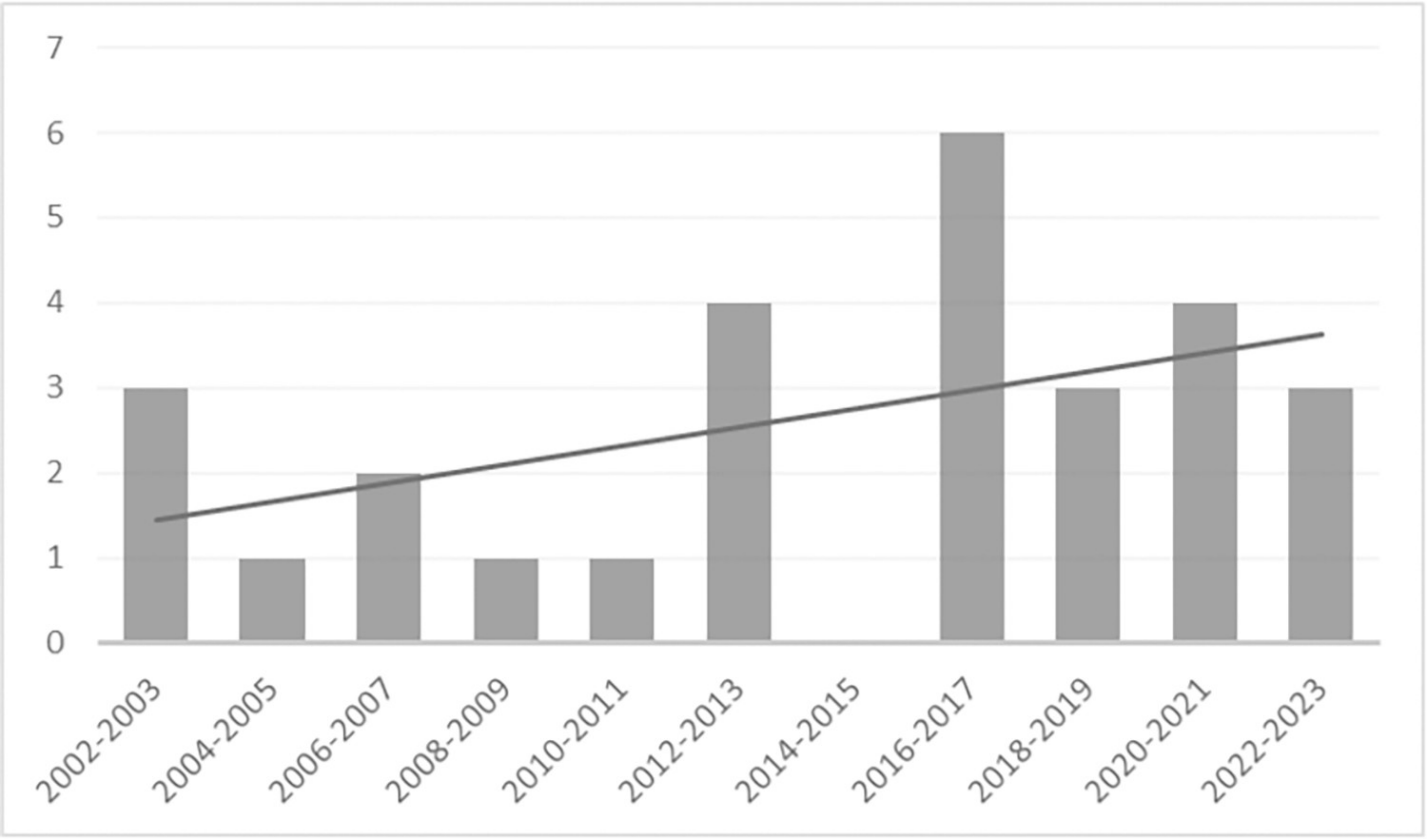
Annual distribution of the selected studies.

The studies in the review were analyzed to obtain a descriptive picture of the research stream on the subject. Descriptive and content information about each included study is displayed in S1 Table. The 28 documents included in the review corpus were published between 2002 and 2023, with a current tendency to rise that could be due to the global focus on climate change and sustainability issues. Fig 2 shows the annual distribution of the selected studies. Researchers are paying more attention to investigating the environmental impacts of tourism and analyzing which practices could bring more sustainability to the sector.

The publication mean is more than one article per year from 2002 to 2013 and, after 2016 almost two articles per year were published. The publication increase could be related to the Sustainable Development Goals from the United Nations (UN), adopted in 2015 through the “2030 Agenda”, and the Paris Climate Agreement, which has established goals to mitigate global warming and promote environmental sustainability. The COVID-19 pandemic could also have a significant impact on the subject, affecting not only the research undergoing in tourism enterprises but even the guests and employees’ behavior during or after the pandemic. Cai and Gou [68] explored the changes in energy consumption of commercial buildings in Singapore during the pandemic impact, and reported that in 93 hotel buildings, the mean EUI indicator had 44.47 kWh/m^2^ of reduction (16%), mainly influenced by the lesser use of Heating, Ventilation, and Air Conditioning (HVAC) systems. It could be expected that the subject will follow this trend in the future years in the context of the Paris Agreement outcomes (investigating progress and challenges), innovations in renewable energy technologies, and transitions to a greener economy.

Asia and Europe have concentrated the research in this field, with 16 and seven investigations representing approximately 82% of the review corpus. Africa (three) and America (one) have four articles, and one research study considered worldwide hotels. The study sites vary from district to country, but fewer articles have looked at the Global South and underdeveloped areas, which, in the tourism case, could represent significant outcomes. The most common publication platforms are the scientific journals Energy and Buildings (eight articles published), Sustainability (three), Journal of Cleaner Production (two), Journal of Sustainable Tourism (two) and International Journal of Hospitality Management (two).

The studies applied different techniques to describe data and assess the effects of contributors on energy use in hotels, correlation analysis (presented in 19 articles), regression techniques (17) and descriptive statistics (15) were by far the most used techniques. Benchmarking techniques (5) together with consumption indicators (13), mainly regarding Energy Use Intensity (EUI) were also used as complementary to other methods. Of the 28 documents, 21 used primary data, which reflects a tendency toward local approaches, e.g., surveys in communities, due to the difficulty of collecting secondary data regarding hospitality operations from reliable sources.

Besides the aspects related to benchmarking and EUI, few theoretical frameworks are proposed in the sampled studies. The majority studies (24) assess the relationship between several variables and energy use in hotels in a descriptive form and does not refer to any developed theory. But three concepts are presented, the Life Cycle Thinking, using Life Cycle Assessment and Cost tools (LCA and LCC), investigated by Bhochhibhoya [46] and Bhochhibhoya et al. [1], the Water-Energy Nexus (WEN), based on Yoon et al. [3], and the Energy Consumption Quota, used in Xin et al. [42].

### Empirical results

The selection of the meta-analysis variables was done from four dimensions that were indicated in the studies: building characteristics, economics, social, and environmental dimensions. The selected contributors to energy use in hotels and their frequency in sampled studies are presented in Table 1.

**Table 1:** Selected contributors to energy use in hotels and their frequency in sampled studies.

Dimension	Variable	Description	Frequency
Building characteristics	Floor area	Hotel gross floor area (m^2^)	30
	Guestrooms	Hotel number of guestrooms	21
	Star level	Hotel category level (1 to 5)	5
	Construction year	Hotel construction year (year)	5
	Building age	Hotel building age (years)	4
	Number of floors	Hotel number of floors (stories)	4
	Guestrooms area	Hotel total guestrooms area (m^2^)	3
	Retrofit	Years after the last retrofit (years)	3
Economics	Occupancy	Hotels occupancy rate (%)	21
	Guest-nights	Number of guest-nights sold	8
	Number of guests	Total number of guests received	5
	Food covers	Number of food covers sold	4
	Room revenue	Revenue per available room or room average rate	4
Social	Number of employees	Hotel total number of employees	7
	Employees density	Number of employees per m^2^	4
Environmental	Carbon emissions	Metric tons of CO_2e_ emissions	3
	Outdoor temperature	Mean outdoor temperature (°C)	3
	Water use	Water used in hotel operation (m^3^)	3

#### Correlational meta-analyses and subgroup analyses

The results of the bivariate correlation coefficients meta-analyses are displayed in Table 2. In which “k” is the number of effect sizes, “N” is the total sample size, “r” is the correlation value or the mean effect size, “CI” is the confidence interval (alpha-value of 5%), “p” is the p-value of the random effects meta-analysis or of the subgroup analysis, and “Q”, “T”, “I^2^” and “FSN” are the estimators of consistency among the studies, as previously presented. The FSN estimate followed Orwin method. The forest plots for all meta-analyses are presented in S1 Fig. Significant subgroup analyses are listed in Table 3, and all subgroup analyses, conducted for variables with more than 10 effect sizes, are in S3 Table.

**Table 2:** Bivariate correlations coefficients meta-analyses.

Contributor	k	N	r	95%-CI	p	Q	T	I^2^ (%)	FSN
Floor area	30	1503	0.6465	[0.4890; 0.7632]	<0.0001	**536.55**	0.6207	94.6	2303
Guestrooms	21	1101	0.5047	[0.2952; 0.6679]	<0.0001	**305.44**	0.5531	93.5	1161
Construction year	5	437	-0.0436	[-0.1862; 0.1007]	0.5546	7.34	0.1077	45.5	31
Star level	5	268	0.5822	[0.3129; 0.7649]	<0.0001	**12.50**	0.2985	68.0	285
Number of floors	4	124	0.3393	[0.0531; 0.5740]	0.0211	6.04	0.2100	50.4	77
Building age	4	103	0.1076	[-0.0971; 0.3036]	0.3029	1.34	0.0000	0.0	32
Guestrooms area	3	108	0.4880	[0.3242; 0.6233]	<0.0001	1.16	0.0000	0.0	145
Retrofit	3	59	0.3587	[0.0264; 0.6197]	0.0350	2.74	0.1638	26.9	54
Occupancy	21	969	0.2029	[-0.0276; 0.4129]	0.0839	**208.19**	0.4882	90.4	336
Guest-nights	8	457	0.7090	[0.5432; 0.8216]	<0.0001	**47.89**	0.3447	85.4	750
Number of guests	5	411	0.6530	[0.0290; 0.9108]	0.0418	**197.46**	0.8461	98.0	381
Food covers	4	275	0.6834	[0.3492; 0.8634]	0.0005	**34.95**	0.4280	91.4	361
Room revenue	4	275	0.8123	[0.5868; 0.9208]	<0.0001	**20.42**	0.4035	85.3	416
Number of employees	7	360	0.7821	[0.6183; 0.8808]	<0.0001	**35.02**	0.3730	82.9	680
Employees density	4	326	0.4514	[0.0669; 0.7192]	0.0230	**30.98**	0.4013	90.3	197
Outdoor temperature	3	225	0.9130	[0.6996; 0.9769]	<0.0001	**12.45**	0.5425	83.9	469
Carbon emissions	3	118	0.8544	[0.5925; 0.9529]	<0.0001	**19.26**	0.4935	89.6	376
Water Use	3	63	0.7786	[0.3849; 0.9326]	0.0013	5.97	0.4493	66.5	356

Note: Q values in bold are statistically significant at 0.01 alpha level.

**Table 3:** Subgroup analyses.

Contributor	Moderator	k	r	95%-CI	p	Q	T	I^2^ (%)
Floor area	Raw data	18	0.8419	[0.7887; 0.8826]	<0.0001	**72.46**	0.2823	76.5
	EUI	12	0.1359	[-0.0098; 0.2759]		**30.79**	0.1942	64.3
Guestrooms	Raw data	13	0.6614	[0.4596; 0.7983]	0.0004	**153.69**	0.5104	92.2
	EUI	8	0.1785	[0.0195; 0.3287]		**13.75**	0.1551	49.1
Occupancy	General	15	0.3557	[0.1153; 0.5567]	0.0099	**149.27**	0.4513	90.6
	Upscale	6	-0.2125	[-0.5237; 0.1487]		**17.54**	0.3741	71.5

Note: Q values in bold are statistically significant at 0.01 alpha level.

**Fig 3:**
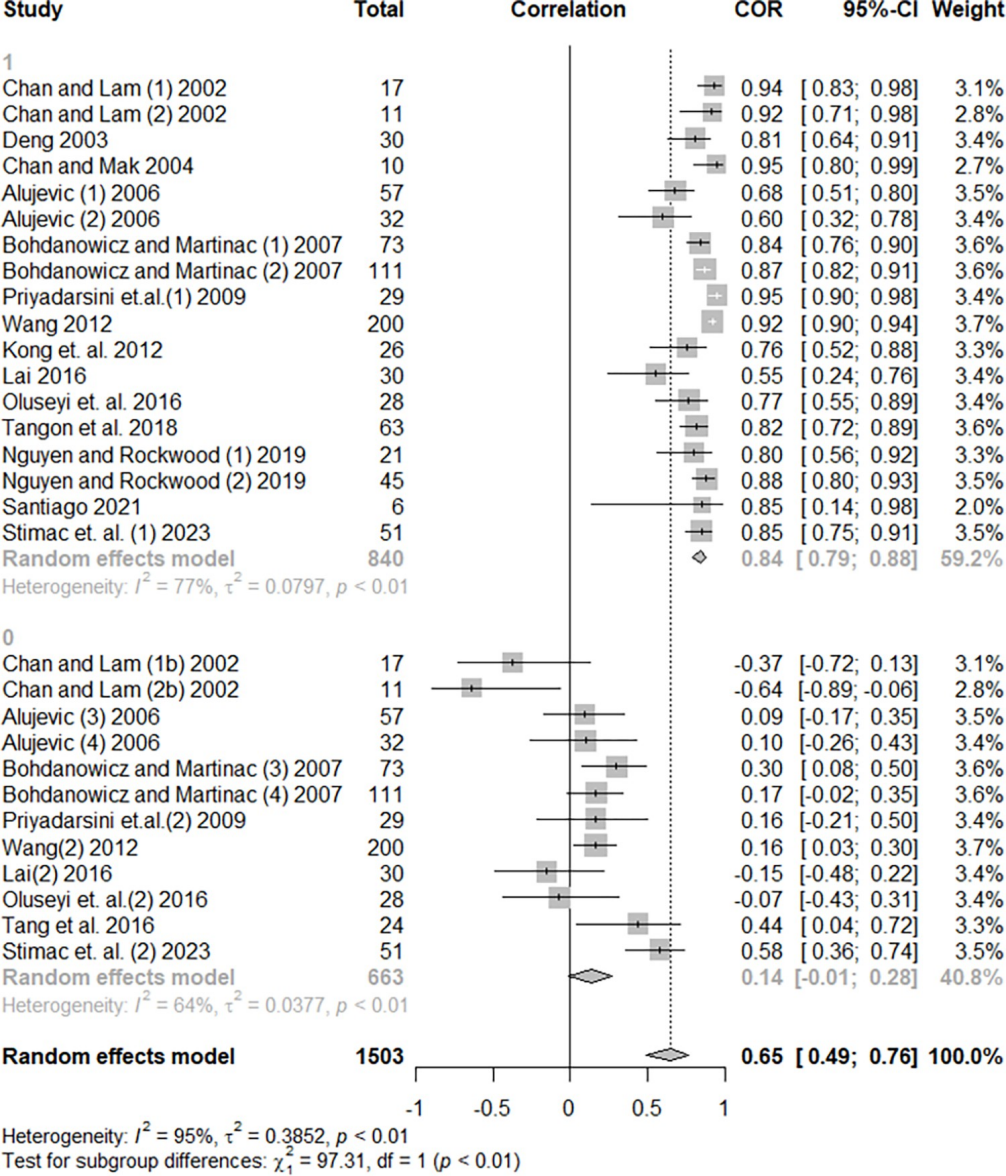
Forest plot of the effect of floor area on hotel energy use.

Considering building characteristics, the hotels’ gross floor area (GFA) resulted in a positive and significant effect on hotel energy use (r = 0.65, p < 0.0001). When considering the T, Q, and I^2^ values, the heterogeneity presented could be assumed to be high. The subgroup analyses revealed that the effect depends on the operationalization of the variable, which is more strongly correlated with energy use when energy is operationalized as raw data (r = 0.84). While the EUI indicator tends to minimize the correlation between GFA and energy use (r = 0.14), it is important to note that in most cases, the EUI is normalized considering the gross floor area, so it is already a value that depends on the area dimension. Fig 3 shows the Forest Plot of the effect of floor area on hotel energy use. The individual effect sizes and the difference when EUI is used can be viewed.

**Fig 4:**
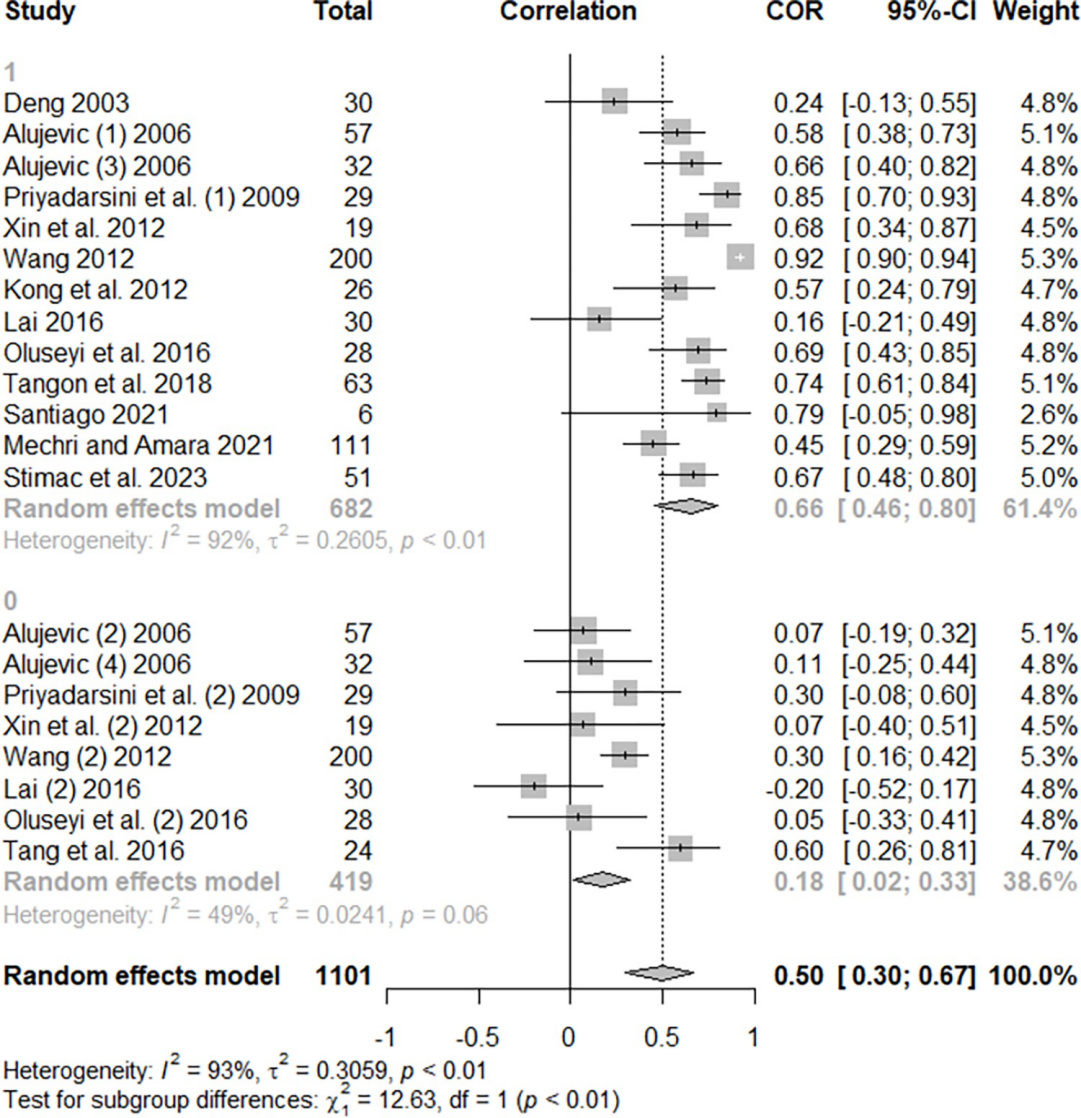
Forest plot of the effect of guestrooms on hotel energy use.

There was a moderately positive and significant effect on hotel energy use when considering the number of guestrooms, with a mean effect of 0.50 (p < 0.0001). The parameters of the heterogeneity were also high, and the subgroup analysis found statistically significant divergence in mean effects due to the operationalization of the energy variable, which presents moderately high positive effect with energy raw data (r = 0.66) and a weak positive effect with EUI indicators (r = 0.18). Fig 4 shows the Forest Plot of the effect of guestrooms on hotel energy use. Another significant subgroup analysis was related to the climatic zones in which the hotels were located (p = 0.0302). While the values in the Equatorial, Tropical, and Temperate zones maintained a moderately high positive behavior (0.45 < r < 0.63), the mean effect size in the sub-tropical zone was low (r = 0.07). This result must be taken with reservations, since the division into four categories generated small subgroups, as seen in S3 Table. The heterogeneity verified in both meta-analyses (GFA and guestroom) will be studied with more depth in meta-regressions. Five studies evaluated the relationship between energy use and the construction year of the hotel buildings, but their results presented high dispersion and did not have a significant mean effect (r = -0.04, p = 0.5546).

The star level of hotels was significant and positively related to energy use in hotels (r = 0.58, p < 0.0001) and the heterogeneity was low compared to other variables. The number of floors, guestroom area, and years after retrofits were also variables that returned statistically significant results but presented weaker relationships with energy use in hotels (r = 0.34; 0.49; and 0.36, respectively). The building age variable, following the outcome of the year of construction, had a low mean effect and a lack of statistical representativeness (r = 0.11, p = 0.3029). The increase in studies with variables that had less than 10 bivariate correlations is important to see if the results are maintained consistently.

**Fig 5:**
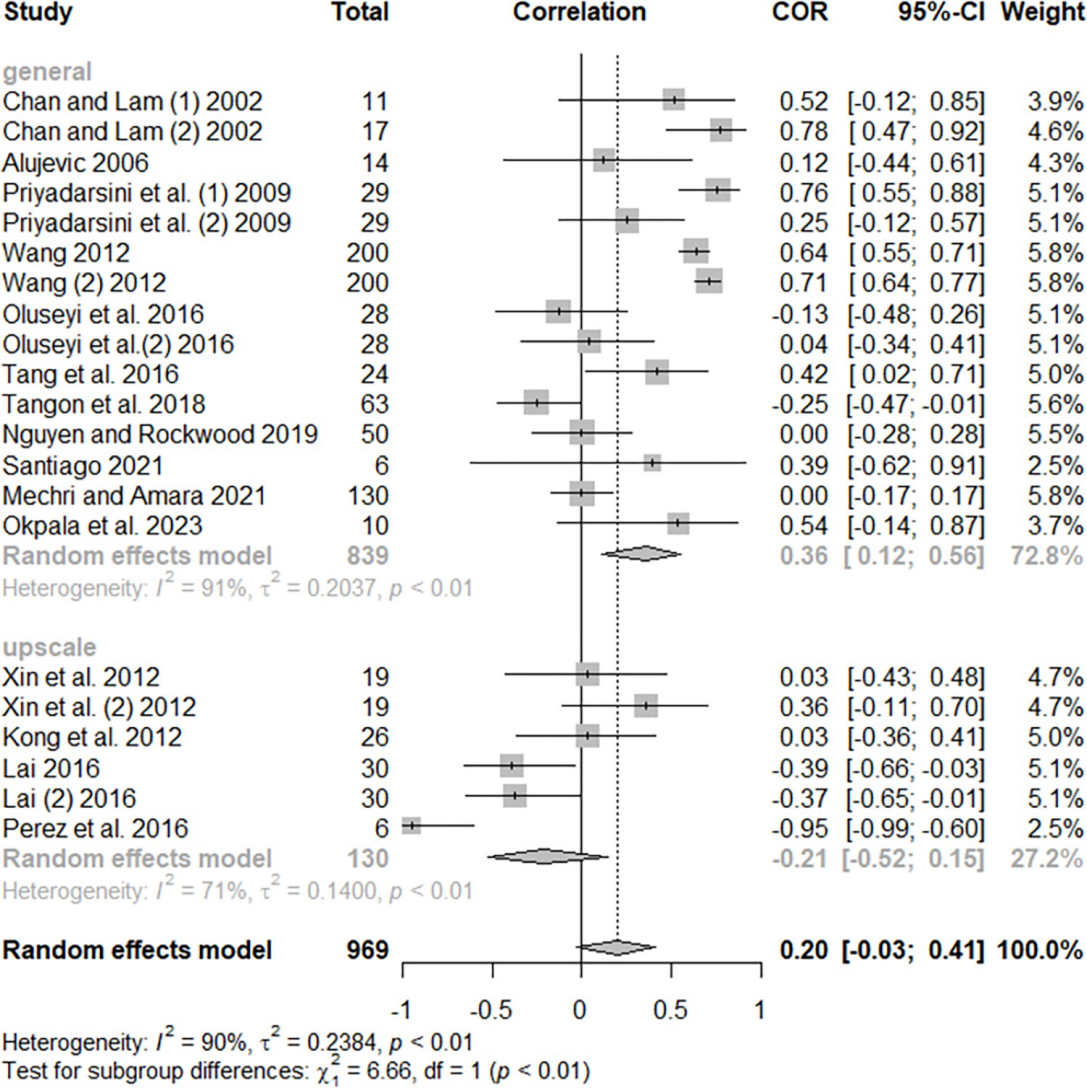
Forest plot of the effect of occupancy on hotel energy use.

In the economic aspect of hotel operation, the occupation variable had no significant relationship with energy use (r = 0.20, p = 0.0839). The subgroup analysis revealed that the effect varies in hotels classified as general (r = 0.36) and those classified as upscale hotels (r = -0.21). A difference in results depending on the methods used was also identified in the occupation variable, studies using regressions presented a positive and low effect size (r = 0.31), while studies that applied only correlations reached a weak and negative effect size (r = -0.04). The test presented a lower statistical significance (p = 0.0456) but it is an important factor to consider. Fig 5 shows the Forest Plot of the effect of occupancy on hotel energy use. The difference between groups was significant (p = 0.0099) and helped to diminish the heterogeneity between studies, but further research considering this variable operationalization will be important to confirm this difference. The number of guest-nights sold, the number of food covers, and the room revenue resulted in positive and significant results, while the number of guests had high variability between studies, which resulted in a positive mean effect with a high dispersion (r = 0.65, p = 0.0418, T = 0.8461, I^2^ = 98%). The number of guest-nights sold (r = 0.71, p < 0.0001) and the number of guests received in the hotels could also represent a part of the occupancy in hotels, therefore signaling the importance of more empirical investigations regarding these variables and energy use, as well as considering the aspects of energy intensive indicators presence or absence. The number of food covers commercialized (r = 0.68, p = 0.0005) represents an aspect related to energy used in the food and beverage department but is also an indicator of hotels’ economic performance. The revenue from the rooms (r = 0.81, p < 0.0001) reflects the stronger economic contributor to hotel energy use but, as with the other significant economic aspects, had fewer studies (four) in comparison with the occupancy variable (20).

The number of employees, in the social dimension, had a strong positive and significant effect on hotel energy use (r = 0.78, p < 0.0001). Employee density, measured by square meter in most cases, had a moderate positive significant effect (r = 0.45, p = 0.0230) with significant heterogeneity when compared to the previous social variable. In the environmental aspect, outdoor temperature (r = 0.91, p < 0.0001), carbon emissions (r = 0.85, p < 0.0001), and water use (r = 0.78, p = 0.0013) presented strong and significant positive relationships with energy use. The social and environmental variables presented significant relationships with energy use but still had fewer studies and variables analyzed when compared with buildings’ characteristics and hotels’ economic performance.

### Publication bias and cumulative evidence

**Fig 6:**
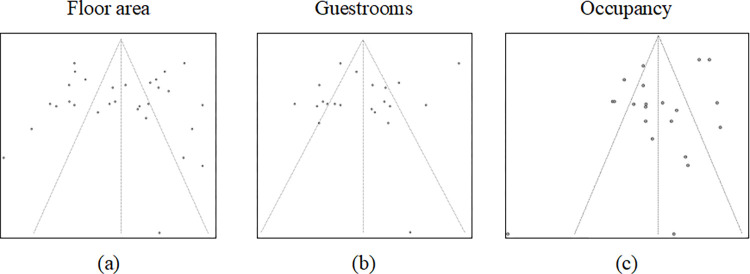
Funnel plots related to floor area, guestrooms, and occupancy variables.

The funnel plot is a mechanism to verify the relationship between study size and effect sizes, where the expected outcome is low standard error in larger studies at the top of the funnel and high standard error at the bottom [59]. The authors affirm that in the absence of bias, roughly half of the studies fall on either side of the mean, but the distribution of the outcomes in the funnel plot is also important. Fig 6 shows the funnel plots to assess small studies’ effects related to floor area, guestrooms, and occupancy variables. These three independent variables were the only ones with more than ten effect sizes and were the only variables that could be analyzed for publication bias.

In the graphical analysis of the funnel plots, the floor area presented a similar pattern along the plot, but it is evident that even the larger studies have a high dispersion from the mean, which could be seen at the top of the graph (a). This difference, as presented in the subgroup analysis and in the meta-analytic regression, could be due to the operationalization of the variable, which in some cases applied raw energy data while in other cases used EUI indicators. Considering the number of guestrooms in hotels, the funnel plot (b) shows less dispersion from the mean distribution when compared to the floor area, even with the same variable operationalization problem, but there seems to be a lack of small studies on both graphs, which is one signal of small study bias. The variable related to occupancy in hotels presented a considerable dispersion at the top of the funnel plot (c) and at one study at the bottom, which could reveal the necessity of bringing more studies related to this variable, as the subgroup analysis and meta-regression also suggested due to the difference among hotel levels.

To quantify evidence for funnel plot symmetry, the rank correlation test and the linear regression test were used. Both tests assume that under the null hypothesis of no small-study effects in a meta-analysis, there is no association between effect size and precision, so if the tests provide significant p-values (p < 0.05), the null hypothesis is rejected, indicating asymmetry of the funnel plot [61]. Table 4 shows the quantitative results for funnel plot asymmetry and Fail-Safe N (FSN) estimations. None of the tests applied in the meta-analyses related to floor area and guestrooms rejected the null hypothesis at a 0.05 level, so there was no quantitative evidence supporting small-study effects in the meta-analyses of floor area and guestroom level related to energy use in hotels. The linear regression test almost indicated asymmetry in the funnel plot of occupancy (p = 0.0591), which can be related to the one study at the bottom of the graph that had a divergent outcome and could represent a case of a small-study effect. Fail-Safe N shows robust values for Floor Area and Guestrooms, using the example of Floor Area, applying Rosenthal’s method, 174,424 studies with null effects would be necessary for the nonsignificant result and 2,303 studies for the effect size to reach the target of 0.01, by the more restrictive Orwin method. Occupancy presented reduced estimates in both methods, as there were no significant results for this variable in the main meta-analysis.

**Table 4:** Quantitative results for funnel plot asymmetry and Fail-Safe N.

	Rank correlation test		Linear regression test		FSN	
	Test result (z)	p-value	Test result (t)	p-value	Rosenthal (N)	Orwin (N)
Floor area	0.00	1.0000	-0.32	0.7526	174424	2303
Guestrooms	0.52	0.6061	-1.43	0.1700	27414	1161
Occupancy	1.27	0.2035	-2.01	0.0591	310	336

To identify patterns during the incorporation of studies, a cumulative meta-analysis was developed for each of the variables according to the factors that were identified as relevant for heterogeneity between studies. In the case of floor area, studies with raw data were incorporated first (from the date of publication) and then studies with normalized energy data (EUI) were selected. In the cases of guestrooms and occupancy, the study precision factor was used, where less precise studies (with smaller samples) were incorporated first. Table 5 shows the cumulative meta-analysis for floor area, guestrooms, and occupancy related to energy use in hotels.

**Table 5:** Cumulative meta-analysis for floor area, guestrooms and occupancy related with energy use in hotels.

Cumulative meta-analysis for floor area					
Add number	Study	Mean effect	p-value	T	I^2^
1	Cham and Lam (2002)–raw data	0.9350	<0.0001	0.0000	0.0
3	Deng (2003)	0.8883	<0.0001	0.2242	42.0
10	Wang (2012)	0.8700	<0.0001	0.3137	82.4
18	Stimac et al. (2023)–raw data	0.8419	<0.0001	0.2823	76.5
19	Cham and Lam (2002)–EUI data	0.8202	<0.0001	0.3622	83.8
22	Alujević (2006)	0.7548	<0.0001	0.5230	91.3
25	Priyadarsini et al. (2009)	0.7086	<0.0001	0.5949	93.7
30	Stimac et al. (2023) EUI data	0.6465	<0.0001	0.6207	94.6
Cumulative meta-analysis for number of guestrooms					
Add number	Study	Mean effect	p-value	T	I^2^
1	Santiago (2021)	0.7940	0.0609	0.0000	0.0
3	Xin et al. (2012)	0.5197	0.0709	0.4357	65.6
6	Oluseyi et al. (2016)	0.5761	<0.0001	0.1766	34.3
10	Deng (2003)	0.5180	<0.0001	0.3543	72.0
16	Alujević (2006)	0.4641	<0.0001	0.3475	75.9
21	Wang (2012)	0.5047	<0.0001	0.5531	93.5
Cumulative meta-analysis for occupancy levels					
Add number	Study	Mean effect	p-value	T	I^2^
1	Perez et al. (2016)	-0.9497	0.0015	0.0000	0.0
2	Santiago (2021)	-0.6090	0.5282	1.4771	86.7
6	Cham and Lam (2002)	0.2424	0.5282	0.6828	77.2
10	Kong et al. (2012)	0.2647	0.0970	0.4034	65.8
15	Lai (2016)	0.2175	0.0987	0.4350	75.7
21	Wang (2012)	0.2029	0.0839	0.4882	90.4

In the cumulative meta-analysis involving floor area and energy use, there was a stabilization of the mean effect and measures of heterogeneity in the studies with raw energy data (r = 0.8419, T = 0.2823, I^2^ = 76.5%). This outcome changed with the inclusion of energy consumption data from EUI indicators, reducing the mean effect size and increasing the measures of heterogeneity (r = 0.6465, T = 0.6207, I^2^ = 94.6). This result indicates that while raw energy use data in hotels has a significant and strong relationship with gross floor area, the normalized energy indicator has a less representative relationship.

As the results of the meta-regressions for the cases involving the guestrooms and occupancy variables resulted in approximately 50% explanation of the variation in the models via the covariates evaluated, a cumulative meta-analysis was conducted according to the precision of the studies. In the case of energy use involved with the number of guestrooms, there was a stable mean effect size (0.46 < r < 0.52), but the heterogeneity increased in the last two studies, which may indicate some point of difference. Occupancy showed high variability in the results, beginning to stabilize its effect size only in the sixth study added (0.20 < r < 0.29), but heterogeneity remained high throughout the sample considered. It is important to bear in mind that in the case of occupancy, there was no significant p-value for the meta-analysis and that different results were found according to hotel category through the analysis of subgroups. S4 Table shows the complete cumulative meta-analyses for the three variables.

To analyze the possible risk of bias in individual studies, an estimate of the cumulative evidence of the meta-analyses was generated. Considering the 25 studies used for the different meta-analyses, the average evidence estimate was 59%, which can be taken as a moderate value. It is important to highlight factors that could be further developed in the literature to achieve studies with greater reliability. Only four studies had sample sizes of more than 100, while 14 studies had samples of less than 30 objects. Increasing sample sizes, while delimiting a region of study, is a fundamental aspect of the precision and generalizability of studies. Similarly, the data collection period for analysis was longer than two years in only six studies, resulting in short-term evaluations that can often lose relevance over time. S5 Table presents the estimate of cumulative evidence from the studies included in meta-analyses.

#### Meta-regression analyses

The results of the meta-analytic regression analyses are presented in Table 6. Where “k” denotes the number of effect sizes, “β” is an estimative of the regression coefficients, “CI” is the confidence interval (alpha-value of 5%), “p” is the p-value of the coefficient estimate, “Q”, “T” and “I^2^” are the estimates of consistency among the studies, and “R^2^” reflects the proportion of total between-study variance explained by the regression model.

**Table 6:** Meta-analytic regression analyses.

Contributor	k	Moderators	β	95%-CI	p	Q	T	I^2^ (%)	R^2^ (%)
Floor area	30	Intercept	0.1241	[-0.0483; 0.2965]	0.1583	**103.25**	0.2483	72.9	84.00
		**Data type**	**1.1023**	**[0.8764; 1.3282]**	**<0.0001**				
Guestrooms	21	**Intercept**	**1.1498**	**[0.0169; 2.2827]**	**0.0271**	**112.92**	0.3841	86.6	51.77
		**Data type**	**0.6260**	**[0.2489; 1.0032]**	**0.0011**				
		Category	-0.2453	[-0.5429; 0.0524]	0.1063				
		Latitude	-0.0063	[-0.0191; 0.0065]	0.3326				
Occupancy	21	Intercept	67.7148	[-0.2415; 135.6711]	0.0508	**67.64**	0.2874	74.9	65.36
		**Category**	**-0.4312**	**[-0.6869; -0.1754]**	**0.0010**				
		**Longitude**	**0.0039**	**[0.0000; 0.0077]**	**0.0484**				
		Year	-0.0330	[-0.0667; 0.0008]	0.0556				

Note 1: Q values in bold are statistically significant at 0.01 alpha level.

Note 2: Moderators in bold are statistically significant at 0.05 alpha level.

Regarding floor area, the meta-regression found that one covariate presented in the studies provided a significant parameter estimate to explain the relationship with energy use. The data type covariate (raw energy consumption, or EUI) had a significant and strong effect on hotel energy use related to GFA (β = 1.1023, p < 0.0001). This estimated coefficient contributed to the regression model to diminish the heterogeneity in the results (Q = 103.25, T = 0.2483, I^2^ = 72.9) and the explanation of approximately 84% of the between-studies variance.

The number of guestrooms in hotels also presented one statistically significant moderator related to energy use in hotels, but two other covariates helped to explain more of the model variance. Data type had a positive and significant effect on hotel energy use related to the guestrooms (β = 0.6260, p = 0.0011). The category of a hotel (β = -0.2453, p = 0.1063) and the latitude of a hotel (β = -0.0063, p = 0.3326) presented negative and non-significant estimators. The meta-regression reduced the heterogeneity between studies (Q = 112.92, T = 0.3841, I^2^ = 86.6%) and explained approximately 52% of the variance.

**Fig 7:**
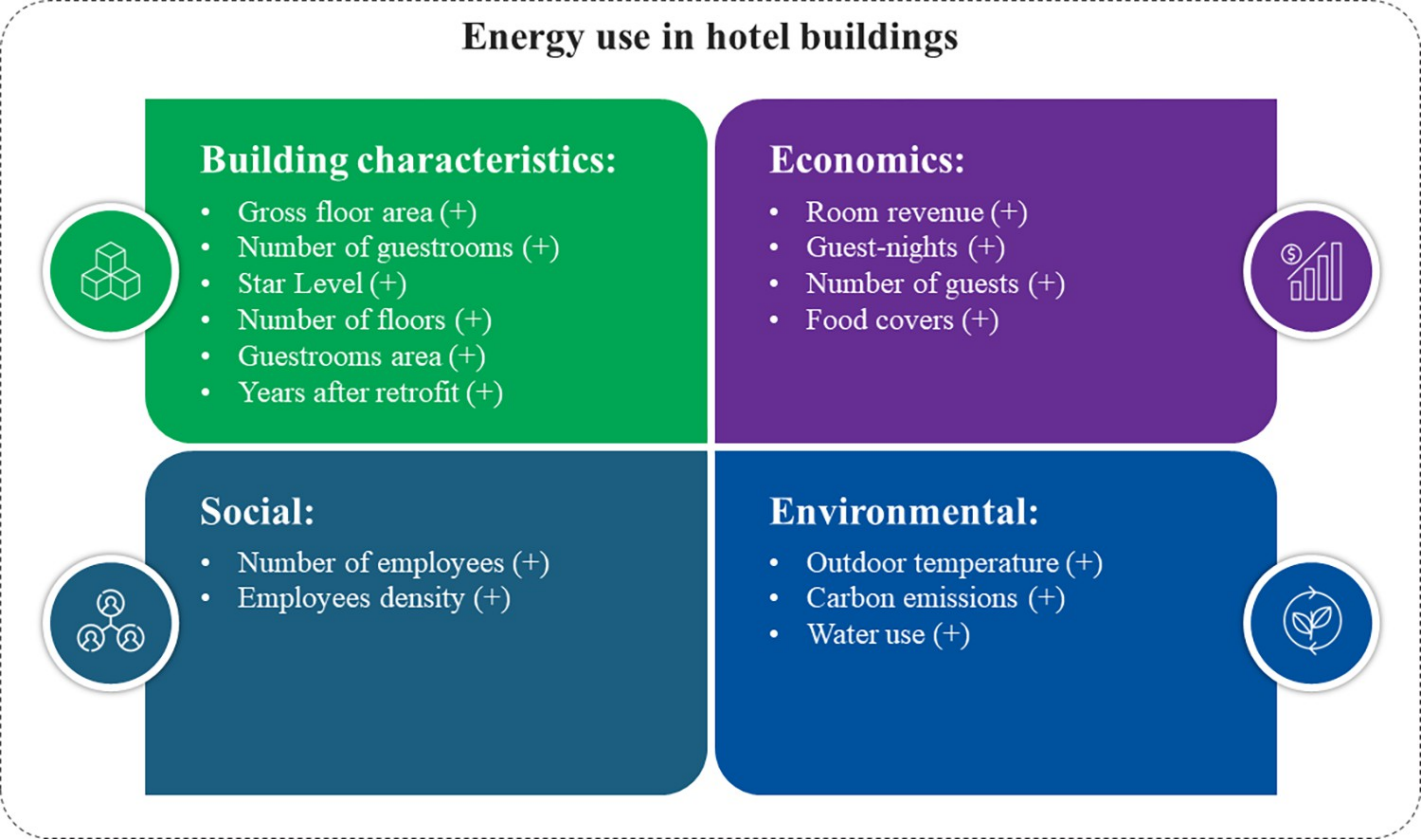
Overview of the significant contributors affecting hotels’ energy use.

In the economic analysis, the occupancy has two statistically significant covariates in the meta-regression, the category level (β = -0.4788, p = 0.0010) and the hotels’ longitude estimate (β = 0.0056, p = 0.0050). It is noted that while the meta-analysis reported a non-significant mean effect size, the operationalization of this contributor, considering hotel star level and its regional characteristics, could be a way to explain the relationship between occupancy and hotel energy use in further studies. The regression model presented less heterogeneity (Q = 94.22, T = 0.3435, I^2^ = 80.9%) and can explain approximately 50% of the between-studies variance. Fig 7 gives an overview of the significant contributors affecting hotels’ energy use.

## Discussion

Of the 18 variables selected for the analysis, only three had more than ten effect sizes, showing a phase of studies focused on physical parameters (floor area and number of guestrooms) and hotel occupancy. This is reflected in a small number of theories being applied and developed and less use of parameters related to the economic, environmental, and social specificities of resource consumption in the sector. In common, the three theories presented refer, in varying degrees, to environmental and economic aspects, with less focus on social issues. Bhochhibhoya et al. [1] state that the use of LCA and LCC can support decisions on environmentally friendly construction technologies, reducing environmental impact and the cost of capital, and resulting in more sustainable operations. Yoon et al. [3] affirm that WEN aims to understand the interactions between water and energy systems, bringing more sustainable resource use and management practices. Xin et al. [42] sustain that setting a reasonable energy quota for buildings could improve energy building efficiency by identifying targets for energy use among different hotel levels.

Although these are interesting proposals for analyzing the issue of resource consumption in the hotel industry, the approaches do not limit how some factors can affect energy use. In this context, it is important to develop a logic that seeks to elucidate how factors from different areas affect resource use in hotels. This study can serve as a starting point for estimating the main contributors and as a basis for further research that evaluates the logic of the relationships found here in different geographical locations, with diverse hotel categories. To this end, some considerations about the relationships between the main contributors and energy use in hotels and the manipulation of this data should be presented to achieve greater standardization and the possibility of replicating outcomes among studies.

The floor area was the variable most used to explain energy use among the studies (16 reports included in the meta-analysis). It can be inferred that there is a significant and strong relationship between energy use (in raw data) and the gross floor area of hotel buildings. This positive relationship becomes weaker when energy intensity indicators (EUI) are used, the most common of which is normalization by floor area (kWh/m^2^). Nine of the studies in the meta-analysis applied correlation analyses for both forms of energy consumption and highlighted the advantages and disadvantages of using raw data and normalized energy efficiency indicators. Six studies conducted the analyses using only raw energy data, while one presented the outcomes considering indicators only. Given the different relationships represented by the two subgroups, it is suggested that original data should always be used, even when normalized data is also considered.

A similar pattern was seen in the operationalization of the guestrooms’ variable. With raw data, the meta-analysis pointed to a moderately positive relationship, while with normalized data, the relationship was positive but weak. Once again, the main EUI normalization factor is the division of gross energy consumption by gross floor area (kWh/m^2^). It can be inferred that data related to the physical parameters of buildings, especially floor area and number of guestrooms, should always consider gross data in their analysis. It is not recommended to exclude the use of EUI, but contextualization and prior analysis of the raw data seem fundamental for a complete evaluation. This finding is reinforced by the results of the meta-analyses for both variables, as well as the subgroup and meta-regression analyses.

Concerning the other variables in the group of physical building characteristics, the category of hotels (star level) showed a positive, moderate, and significant relationship with energy use, as well as the guestroom area. The number of floors and the years after retrofit variables showed moderately weaker relationships with energy use, and the year of construction and the buildings’ age did not result in a significant effect size. It should be noted that these variables have only been studied in between three and five studies, so their results cannot be considered representative. Future research could expand the area of knowledge by including these variables.

In the occupancy rate, the meta-analysis was not able to indicate a significant effect size however, indications of factors influencing its relationship with energy consumption can be inferred. In the subgroup analysis, unlike the variables related to physical characteristics, there was no differentiation due to the type of energy data used, but rather in terms of the hotels’ categories considered. The relationship between occupancy and energy use in general hotels (3 stars or less) was moderately positive, while in upscale hotels, the effect was weak and negative. This indicates that the occupancy rate has a slightly positive influence on energy use in general hotels, while in higher category hotels, this relationship is weaker and inversely related to energy use. As seen in some studies [10, 19, 21, 24, 40, 41, 45], this may be related to the number of extra services available in upscale hotels and the high energy demand of these spaces. Furthermore, through meta-regression, the influence of the covariate of hotel categories can be visualized, and an influence, on a smaller scale, of the covariate is related to the estimated longitude of these hotels. The influence verified through longitude may be due to a series of factors, such as the climate and energy demand of the location, the amount of sunlight perceived, the energy infrastructure, and available resources. There is a high need for further research into occupancy rates and their relationship to energy use, while subgroups with hotel levels, locations, and services offered should also be included in the investigations.

In the other economic variables related to energy use, the results pointed to a moderately strong positive and statistically significant relationship for all the variables with energy use. However, apart from the variable related to guest-nights, which was analyzed eight times, the others had only five (number of guests) or four (room revenue and food covers) effect sizes. This shows that although economic variables are statistically significant for understanding energy use in hotels, few studies have used them. Further research should consider the economic variables presented in this study, with an extra recommendation to incorporate new economic variables from company annual reporting methodologies such as the GRI.

The incorporation of new variables from annual reporting methodologies should also be considered in the social and environmental areas. The number of employees showed a strong and statistically significant positive mean effect size, while the density of employees showed a moderately significant relationship, with a similar pattern to the floor area and guestroom variables. In the social sphere, future studies can enrich the analyses by considering the number of female employees, staff turnover, and number of training hours, among others. On the environmental issue, even with a few studies, a major influence can be seen on the consumption of other resources linked to energy use, such as water use and carbon emissions. The waste generated can also be a factor to be added to future analyses. The external temperature also had a strong influence on energy use, which should depend, as well as the occupancy rate, to a greater or lesser extent on the geographic location. It is important to highlight that both social and environmental aspects must be expanded in further studies to confirm the relationships presented in the meta-analyses.

These outcomes suggest the importance of considering the hotels’ scale, structure, and services when planning the buildings’ energy efficiency. Larger hotels with more rooms should implement specific strategies to manage their resource consumption. Hotel chains should focus on energy-efficient technologies and operational practices adapted to the hotels’ size and occupancy. This includes using advanced Energy Management Systems (EMS), retrofitting buildings with greener technologies, and optimizing services to minimize energy waste. Adapting sustainable practices according to the hotels’ quality level can help balance service offerings with energy efficiency. Upscale hotels, in particular, could invest in strategies to increase occupancy rates, as their energy use remains high due to their extensive structures and services provided, even with low occupancy. This approach will promote sustainability and optimize operational costs.

It is also important to highlight that the relationships found by several studies evaluated can be related to theories broadly linked to the theme of sustainable development, among which the Stakeholders Theory and the Triple Bottom Line (TBL). The first is related to meeting the expectations of various groups, such as customers, employees, investors, local communities and government agents, promoting a positive image and a reduced environmental impact through sustainable measures in an upper emission sector. The second involves seeking a balance between financial results and practices that benefit society and minimize environmental impact, forming an economic, social and environmental junction to guarantee long-term sustainable development.

The Stakeholders perspective corroborates other studies, such as Theodoulidis et al. [69], who, analyzing the tourism sector (airlines, casinos, hotels, and restaurants) suggest that different stakeholders’ interests moderate companys’ strategic decisions and that these effects become stronger in the long term. MackAskill et al. [43] estate that the effectiveness of programs to reduce resource use is dependent on influence of the stakeholder, where initiatives to reduce water consumption are proved to be well placed with hotel guest on their research. The TBL approach is also in line with other results, like Assaf et al. [70], which sustain that an increase in environmental, social, and financial reporting has a significant impact on hotel performance. Lai and Lu [71] suggested that capital projects, especially those energy retrofits, can significantly reduce carbon emission while consuming less energy, which reflect the importance of a broad funding energy policy specifically for low-end or small hotel companies which are less motivated to implement capital projects to mitigate climate changes.

It should be noted that the Floor Area, Guestrooms, and Occupancy variables had a minimum of 10 effect sizes for meta-analysis, leading to a broad and representative analysis of these relationships with energy use in hotels, including subgroup analysis and meta-regression. This was not the case with the other 15 variables extracted in the research, which had less than 10 effect sizes in each analysis and should be interpreted with greater caution. The less representative variables could help to delimit relationships between contributors and energy use in hotels at an early stage, being a reference for further studies. Structurally adapted from Luceri et al. [72], Table 7 summarizes the key findings, theoretical and practical implications, and future research directions.

**Table 7:** Key findings, theoretical and practical implications and future research directions.

Key findings and theoretical implications	Managerial/practical implications
Effects of previous variables on energy use in hotel buildings were reviewed and synthesized	Consider the scale, structure, and services of hotels when planning the buildings’ energy efficiency
High concentration of articles in Asia and Europe	Need for research in more regions
Gross Floor Area and Total Number of Guestrooms affect hotel energy use positively	Larger hotels with more rooms should implement strategies to manage their resource consumption
Occupancy had inconclusive results, with hotel star level affecting the outcome	Upscale hotels could invest in strategies to increase occupancy rates avoiding excessive energy use
13 contributors to energy use in hotels are evaluated	Use of advanced EMS, retrofitting buildings with greener technologies, and optimizing services to minimize energy waste
Divergences in using raw energy data and EUI indicators	Consider raw energy data when evaluating the relationship with hotel physical parameters
**Future research directions**	
**Methodology**	
Development and investigation of theories to support the relationship in the empirical literature	
Incorporation of new variables should be considered mainly in the social and environmental areas (e.g., female employees, staff turnover, hours of training, waste generated)	
Role of company annual reporting methodologies such as GRI	
The contribution of the COVID-19 Pandemic in the relationship between contributors and energy use in hotels	
Development of longitudinal studies to evaluate the dynamics among variables	
**Contributors to energy use**	
Expand research on occupancy rates and their relationship to energy use, including possible moderators	
Expand social and environmental relationships with energy use in hotels (e.g., number of employees, carbon emissions, water use, ambient temperature)	
Expand the area of knowledge by considering significant underrepresented variables (star level, retrofit, guestrooms area, guest-nights, room revenue, among others)	
**Moderators**	
Include hotel levels, locations, and services offered in further investigations	
Evaluate potential impacts of innovative technologies, such as EMS and smart meters	

## Conclusion

The main objective of this article was to synthesize knowledge about the possible contributors to energy use in hotel buildings. The content of 28 articles was systematically analyzed to provide a descriptive view of the research stream. The primary studies are, in most cases, based on primary data, without theory formulation and with a lack of standardized data. The high concentration of articles in Asia (57%) and Europe (25%) also stood out, generating a lack of a global overview of the contributors to energy use in hotels, making it clear that there is a need for research in the other regions, especially in the global south, because these relationships may differ due to local issues and large countries (e.g., USA, Brazil, India) are not represented in the corpus research.

Using the meta-analytic procedure, the review integrated the results of the explicative studies, in which the direct effect of 18 contributors to energy use in hotels was assessed by estimating mean effect sizes based on the primary studies. Previous research led to some conclusive findings in cases like the effects of floor area and number of guestrooms on raw energy data, while the occupancy rate had inconclusive results when considering all the hotels, but an explanation was found regarding the hotel categories. From the other 15 variables, there were no conclusive outcomes due to the reduced number of effect sizes considered in primary studies (less than 10 per variable), but statistically significant mean effect sizes were retrieved from 13 variables (hotel star level, number of floors, guestroom area, years after last retrofit, number of guest-nights, number of guests, food covers, room revenue, number of employees, employee density, outdoor temperature, carbon emissions and water use). This shed light on the possible variables contributing to energy use in hotels and could be the basis for further studies aiming to improve energy efficiency.

Although meta-analyses are frequently referred to as a powerful statistical method to summarize empirical evidence, considerations must be made. The possible meta-bias was verified in graphical and statistical tests, where a high variation was seen throughout all three main meta-analyses, but the only test that showed signals of small-studies effects was concerning the occupancy rate, which had already failed to show a significant result in the meta-analysis. Furthermore, through the cumulative meta-analysis, the patterns identified in the subgroup and the meta-regression analysis could be represented and ratified. The cumulative evidence, seeking to consider possible individual study bias, resulted in a moderate value (59%), which can be attributed to the low sample size and short analysis intervals normally applied in corpus studies. It can be inferred that the field of research is at an early stage, as there is no systematic and repetitive analysis among studies since the variables considered vary in type and form, which brings the importance of standardizing further studies with variables and indicators that are widespread, representative, and easy to collect in the hotel chain.

Limitations must also be acknowledged. The review considered all languages but only applied English keywords, so all the selected articles were written in this language. A level of publication bias could be present in the study even with the results of the funnel plot and statistical tests because these tests are mainly effective in estimating small-study effects, while publication bias is a more comprehensive topic. The choice of keywords applied in the search could also not represent relevant articles and could have included non-relevant studies. Even with the application of subgroup analysis and meta-regression (considering aspects like hotel category, geographical location, and type of energy data), differences in methodologies, hotels’ physical characteristics (spa, pools, among others), and environmental conditions were not considered due to a lack of reliable data. Some specific studies resulted in diverging outcomes, but the review does not assess this issue by, for example, applying the trim and fill methodology to understand the behavior of excluding and adding articles in the analysis. Further meta-analyses could review the outcomes presented in this study and investigate the presented limitations. New applied research on the topic could be conducted covering periods before, during, and after the COVID-19 pandemic. Further meta-analysis reviews evaluation involving data from years of health crisis may be used, such as in subgroup analyses, to identify possible divergences with other periods due to potential variations in the use of HVAC systems, occupancy rates, consumer behavior, and the adoption of energy management systems.

Considering the implications of the study, the review synthesizes the empirical research on energy use in hotel buildings and can serve to recommend further research on this topic. The development and investigation of theories to support the relationship found in the empirical literature seems to be the main research gap to be studied. The review also supports the use of different variables aiming to explain energy use in hotels, with special attention to those with significant influence but fewer studies and those regarding global standardized methods, like GRI variables. Scholars should be cautious regarding the use of energy indicators to operationalize the energy data, the indicators are interesting in understanding the intensity of consumption regarding hotel characteristics, but the use of raw data is necessary to investigate the relationship with the contributors in a direct form, some classification could be made afterwards.

From a social, economic, and environmental point of view, the study contributes by highlighting the significant contributions of variables related to business sustainability to energy use in hotel buildings. The results can help managers build measures to improve energy efficiency and mitigate costs, which can lead to better financial planning and the creation of spaces with a sustainable ideal number of guests and employees. Legislators can develop tariff proposals according to energy demand classes standardized by representative indicators, as well as subsidies for good energy use practices adopted by hotel units and other financial measures that encourage the implementation of renewable technologies such as solar and wind energy. In this way, the work conveys a sustainable importance for today’s hotel operations, which can be decisive in achieving less carbon-intensive practices and reducing the sector’s carbon footprint.

## Supporting information

S1 Checklist PRISMA 2020 checklist. (PDF)

S1 Table Information about each included study. (PDF)

S2 Table Independent variables retrieved from the included studies. (PDF)

S3 Table Subgroup analyses for variables with more than 10 effect sizes. (PDF)

S4 Table Complete cumulative meta-analyses. (PDF)

S5 Table Estimate of cumulative evidence from the studies included in meta-analysis. (PDF)

S1 Appendix Meta-analyses coding book. (PDF)

S1 Fig Forest plots for all meta-analyses. (PDF)

## References

[1] Bhochhibhoya S, Pizzol M, Marinello F, Cavalli R. Sustainability performance of hotel buildings in the Himalayan region. J Clean Prod. 2020;250. doi: 10.1016/j.jclepro.2019.119538

[2] Mechri H eddine, Amara S. Investigation and analysis of energy and water use of hotel buildings in Tunisia. Energy Build. 2021;241. doi: 10.1016/j.enbuild.2021.110930

[3] Yoon H, Sauri D, Rico A. The water-energy nexus in hotels and recreational activities of a mass tourism resort: the case of Benidorm. Curr Issues Tour. 2022;25: 592–610. doi: 10.1080/13683500.2021.1893283

[4] Fatima T, Elbanna S. Advancing sustainable performance management in the hospitality industry: A novel framework based on a health-inclusive balanced scorecard. Tour Manag Perspect. 2023;48. doi: 10.1016/j.tmp.2023.101141

[5] Majid GM, Tussyadiah I, Kim YR, Pal A. Intelligent automation for sustainable tourism: a systematic review. J Sustain Tour. 2023;31: 2421–2440. doi: 10.1080/09669582.2023.2246681

[6] Rahman I, Chen H, Bernard S. The incidence of environmental status signaling on three hospitality and tourism green products: a scenario-based quasi-experimental analysis. Tour Manag Perspect. 2023;46. doi: 10.1016/j.tmp.2023.101076

[7] López-Gamero MD, Molina-Azorín JF, Tarí JJ, Pertusa-Ortega EM. Interaction between sustainability practices and the mediating role of hotel performance. J Sustain Tour. 2023;0: 1–26. doi: 10.1080/09669582.2023.2198165

[8] Statista. Sustainable tourism worldwide—statistics and facts. New York; 2023. Available: https://www.statista.com/topics/1916/green-tourism/#topicOverview

[9] Garrod B, Zhao AL, Koenig-Lewis N. A greener way to stay: The role of perceived sustainability in generating loyalty to Airbnb. Int J Hosp Manag. 2023;110: 103432. doi: 10.1016/j.ijhm.2023.103432

[10] Tangon S, Chontanawat J, Chiarakorn S. Factors affecting electricity consumption intensity of hotel buildings in Thailand. Asia-Pacific J Sci Technol. 2018;23.

[11] Bux C, Aluculesei AC, Moagăr-Poladian S. How to Monitor the Transition to Sustainable Food Services and Lodging Accommodation Activities: A Bibliometric Approach. Sustain. 2022;14: 1–21. doi: 10.3390/su14159102

[12] Gössling S, Scott D. The decarbonisation impasse: global tourism leardes’ views on climate change mitigation. J Sustain Tour. 2018;26. doi: 10.1080/09669582.2018.1529770

[13] IPCC. Climate Change 2023: Synthesis Report. Contribution of Working Groups I, II and III to the Sixth Assessment Report of the Intergovernmental Panel on Climate Change. Geneva; 2023. doi: 10.59327/IPCC/AR6-9789291691647

[14] Gössling S, Balas M, Mayer M, Sun Y. A review of tourism and climate change mitigation: the scales, scopes, stakeholders and strategies of carbon management. Tour Manag. 2023;95. doi: 10.1016/j.tourman.2022.104681

[15] Steiger R, Demiroglu OC, Pons M, Salim E. Climate and carbon risk of tourism in Europe. J Sustain Tour. 2022;0: 1–31. doi: 10.1080/09669582.2022.2163653

[16] Migdadi YKAA. Identifying the Best Practices in Hotel Green Supply Chain Management Strategy: A Global Study. J Qual Assur Hosp Tour. 2023;24: 504–544. doi: 10.1080/1528008X.2022.2065657

[17] Xuchao W, Priyadarsini R, Siew Eang L. Benchmarking energy use and greenhouse gas emissions in Singapore’s hotel industry. Energy Policy. 2010;38: 4520–4527. doi: 10.1016/j.enpol.2010.04.006

[18] Lai JHK. Energy use and maintenance costs of upmarket hotels. Int J Hosp Manag. 2016;56: 33–43. doi: 10.1016/j.ijhm.2016.04.011

[19] Santiago DE. Energy use in hotels: A case study in Gran Canaria. Int J Low-Carbon Technol. 2021;16: 1264–1276. doi: 10.1093/ijlct/ctab048

[20] Palani H, Karatas A. Identifying energy-use behavior and energy-Use profiles of hotel guests. Appl Sci. 2021;11. doi: 10.3390/app11136093

[21] Tang M, Fu X, Cao H, Shen Y, Deng H, Wu G. Energy performance of hotel buildings in Lijiang, China. Sustain. 2016;8. doi: 10.3390/su8080780

[22] Štimac M, Matković M, Karasalihović Sedlar D. Correlations between Hotel Size and Gas Consumption with a Feasibility Analysis of a Fuel Switch—A Coastal Case Study Croatia Adriatic. Sustain. 2023;15. doi: 10.3390/su15118595

[23] Perez FJD, Chinarro D, Mouhaffel AG, Martin RD, Otin RP. Modelling of energy and water supplies in hotels in lanzarote and fuerteventura with and without desalination plant (SWROP). Indian J Sci Technol. 2016;9: 1–19. doi: 10.17485/ijst/2016/v9i47/101908

[24] Oluseyi PO, Babatunde OM, Babatunde OA. Assessment of energy consumption and carbon footprint from the hotel sector within Lagos, Nigeria. Energy Build. 2016;118: 106–113. doi: 10.1016/j.enbuild.2016.02.046

[25] Cabello Eras JJ, Sousa Santos V, Sagastume Gutiérrez A, Guerra Plasencia MÁ, Haeseldonckx D, Vandecasteele C. Tools to improve forecasting and control of the electricity consumption in hotels. J Clean Prod. 2016;137: 803–812. doi: 10.1016/j.jclepro.2016.07.192

[26] Priyadarsini R, Xuchao W, Eang LS. A study on energy performance of hotel buildings in Singapore. Energy Build. 2009;41: 1319–1324. doi: 10.1016/j.enbuild.2009.07.028

[27] Wang JC. A study on the energy performance of hotel buildings in Taiwan. Energy Build. 2012;49: 268–275. doi: 10.1016/j.enbuild.2012.02.016

[28] Papageorgiou G, Efstathiades A, Nicolaou N, Maimaris A. Energy management in the hotel industry of Cyprus. 2018 IEEE Int Energy Conf ENERGYCON 2018. 2018; 1–5. doi: 10.1109/ENERGYCON.2018.8398763

[29] Campagna LM, Fiorito F. On the Impact of Climate Change on Building Energy Consumptions: A Meta‐Analysis. Energies. 2022;15. doi: 10.3390/en15010354

[30] Hu M, Zhang K, Nguyen Q, Tasdizen T. The effects of passive design on indoor thermal comfort and energy savings for residential buildings in hot climates: A systematic review. Urban Clim. 2023;49: 101466. doi: 10.1016/j.uclim.2023.101466

[31] Gao YL, Mattila AS, Lee S. A meta-analysis of behavioral intentions for environment-friendly initiatives in hospitality research. Int J Hosp Manag. 2016;54: 107–115. doi: 10.1016/j.ijhm.2016.01.010

[32] Dimara E, Manganari E, Skuras D. Don’t change my towels please: Factors influencing participation in towel reuse programs. Tour Manag. 2017;59: 425–437. doi: 10.1016/j.tourman.2016.09.003 PMC5137323 27942562

[33] Yang Y, Park S, Hu X. Electronic word of mouth and hotel performance: A meta-analysis. Tour Manag. 2018;67: 248–260. doi: 10.1016/j.tourman.2018.01.015

[34] Kong X, Lu S, Gao P, Zhu N, Wu W, Cao X. Research on the energy performance and indoor environment quality of typical public buildings in the tropical areas of China. Energy Build. 2012;48: 155–167. doi: 10.1016/j.enbuild.2012.01.021

[35] Chan WW, Lam JC. Prediction of pollutant emission through electricity consumption by the hotel industry in Hong Kong. Int J Hosp Manag. 2002;21: 381–391. doi: 10.1016/S0278-4319(02)00027-0

[36] Chan WW, Lam JC. A study on pollutant emission through gas consumption in the Hong Kong hotel industry. J Sustain Tour. 2002;10: 70–81. doi: 10.1080/09669580208667153

[37] Nguyen AT, Rockwood D. Developing an energy benchmarking system for hotel buildings using the statistical method and the simulation-based approach. J Green Build. 2019;14.

[38] Deng S. Energy and water uses and their performance explanatory indicators in hotels in Hong Kong. Energy Build. 2003;35: 775–784. doi: 10.1016/S0378-7788(02)00238-4

[39] Chan WW, Mak BL. An estimation of the environmental impact of diesel oil usage in Hong Kong hotels. J Sustain Tour. 2004;12: 346–355. doi: 10.1080/09669580408667242

[40] Alujević VZ. Energy use and environmental impact from hotels on the Adriatic Coast in Croatia—current status and future possibilities for HVAC systems. Royal Institute of Technology—Sweden. 2006.

[41] Bohdanowicz P, Martinac I. Determinants and benchmarking of resource consumption in hotels-Case study of Hilton International and Scandic in Europe. Energy Build. 2007;39: 82–95. doi: 10.1016/j.enbuild.2006.05.005

[42] Xin Y, Lu S, Zhu N, Wu W. Energy consumption quota of four and five star luxury hotel buildings in Hainan province, China. Energy Build. 2012;45: 250–256. doi: 10.1016/j.enbuild.2011.11.014

[43] MacAskill S, Becken S, Coghlan A. Engaging hotel guests to reduce energy and water consumption: A quantitative review of guest impact on resource use in tourist accommodation. Clean Responsible Consum. 2023;11. doi: 10.1016/j.clrc.2023.100156

[44] Okpala C, Njoku H, Ako P. A Data Envelopment Analysis to Benchmark Hotel Energy Consumption in an Urban Locality †. Eng Proc. 2023;53: 1–7. doi: 10.3390/IOCBD2023-15204

[45] Wang JC, Huang KT. Energy consumption characteristics of hotel’s marketing preference for guests from regions perspective. Energy. 2013;52: 173–184. doi: 10.1016/j.energy.2013.01.044

[46] Bhochhibhoya S. Sustainability assessment of building system in Himalayan region. Università degli Studi di Padova. 2016.

[47] Arenhart RS, Souza AM, Zanini RR. Energy Use and Its Key Factors in Hotel Chains. Sustain. 2022;14: 1–14. doi: 10.3390/su14148239

[48] Passer A, Kreiner H, Maydl P. Assessment of the environmental performance of buildings: a critical evaluation of the influence of technical building equipment on residential buildings. Int J Life Cycle Assess. 2012;17. doi: 10.1007/s11367-012-0435-6

[49] Zhang J, Yuan C, Yang J, Zhao L. Research on Energy Consumption Prediction Models for High-Rise Hotels in Guangzhou, Based on Different Machine Learning Algorithms. Buildings. 2024;14: 356. doi: 10.3390/buildings14020356

[50] Page MJ, McKenzie JE, Bossuyt PM, Boutron I, Hoffmann TC, Mulrow CD, et al. The PRISMA 2020 statement: An updated guideline for reporting systematic reviews. BMJ. 2021;372. doi: 10.1136/bmj.n71 33782057 PMC8005924

[51] Shamseer L, Moher D, Clarke M, Ghersi D, Liberati A, Petticrew M, et al. Preferred reporting items for systematic review and meta-analysis protocols (prisma-p) 2015: Elaboration and explanation. BMJ. 2015;349: 1–25. doi: 10.1136/bmj.g7647 25555855

[52] Greene D, Demeter C, Dolnicar S. The Comparative Effectiveness of Interventions Aimed at Making Tourists Behave in More Environmentally Sustainable Ways: A Meta-Analysis. J Travel Res. 2023. doi: 10.1177/00472875231183701

[53] Bailey N. Exploring the relationship between institutional factors and FDI attractiveness: A meta-analytic review. Int Bus Rev. 2018;27: 139–148. doi: 10.1016/j.ibusrev.2017.05.012

[54] Song W, Zhang Z, Chen Z, Wang F, Yang B. Thermal comfort and energy performance of personal comfort systems (PCS): A systematic review and meta-analysis. Energy Build. 2022;256. doi: 10.1016/j.enbuild.2021.111747

[55] Schmid D, Morschett D. Decades of research on foreign subsidiary divestment: What do we really know about its antecedents? Int Bus Rev. 2020;29. doi: 10.1016/j.ibusrev.2019.101653

[56] Paul J, Criado AR. The art of writing literature review: What do we know and what do we need to know? Int Bus Rev. 2020;29. doi: 10.1016/j.ibusrev.2020.101717

[57] Rodrigues SD, Garcia VJ. Transactive energy in microgrid communities: A systematic review. Renew Sustain Energy Rev. 2023;171. doi: 10.1016/j.rser.2022.112999

[58] Feil AA, Schreiber D, Haetinger C, Haberkamp ÂM, Kist JI, Rempel C, et al. Sustainability in the dairy industry: a systematic literature review. Environ Sci Pollut Res. 2020;27: 33527–33542. doi: 10.1007/s11356-020-09316-9 32566986

[59] Borenstein M, Hedges L V., Higgins JPT, Rothstein HR. Introduction to meta-analysis. 2nd ed. Hoboken: John Wiley & Sons; 2021.

[60] Hedges L V., Olkin I. Statistical methods for meta-analysis. Orlando: Academic Press; 1985.

[61] Schwarzer G, Carpenter JR, Rücker G. Meta-Analysis with R. Heidelberg: Springer Cham; 2015.

[62] Higgins JPT, Thompson SG, Deeks JJ, Altman DG. Measuring inconsistency in meta-analyses. BMJ. 2003;327. doi: 10.1136/bmj.327.7414.557 12958120 PMC192859

[63] Baker WL, White CM, Cappelleri JC, Kluger J, Coleman CI. Understanding heterogeneity in meta-analysis: the role of meta-regression. Int J Clin Pract. 2009;63. doi: 10.1111/j.1742-1241.2009.02168.x 19769699

[64] Begg CB, Mazumdar M. Operating characteristics of a rank correlation test for publication bias. Biometrics. 1994;50. 7786990

[65] Egger M, Smith GD, Schneider M, Minder C. Bias in meta-analysis detected by a simple graphical test. Br Med J. 1997;315. doi: 10.1136/bmj.315.7109.629 9310563 PMC2127453

[66] Rosenthal R. The file dawer problem and tolerance for null results. Psychol Bull. 1979;86: 638.

[67] Orwin RG. A fail-safe N for effect size in meta-analysis. J Educ Stat. 1983;8: 157–159.

[68] Cai S, Gou Z. Impact of COVID-19 on the energy consumption of commercial buildings: A case study in Singapore. Energy Built Environ. 2024;5: 364–373. doi: 10.1016/j.enbenv.2022.11.004

[69] Theodoulidis B, Diaz D, Crotto F, Rancati E. Exploring corporate social responsibility and financial performance through stakeholder theory in the tourism industries. Tour Manag. 2017;62: 173–188. doi: 10.1016/j.tourman.2017.03.018

[70] Assaf AG, Josiassen A, Cvelbar LK. Does Triple Bottom Line reporting improve hotel performance? Int J Hosp Manag. 2012;31: 596–600. doi: 10.1016/j.ijhm.2011.08.005

[71] Lai JHK, Lu M. Carbon emission and maintenance cost of commercial buildings: Quantification, analysis and benchmarking. J Clean Prod. 2024;447: 141459. doi: 10.1016/j.jclepro.2024.141459

[72] Luceri B, (Tammo) Bijmolt THA, Bellini S, Aiolfi S. What drives consumers to shop on mobile devices? Insights from a Meta-Analysis. J Retail. 2022;98: 178–196. doi: 10.1016/j.jretai.2022.02.002

