# Supplementary material for: Energy use and its contributors in hotel buildings: A systematic review and meta-analysis
Source: PLoS One. 2024 Oct 24;19(10):e0309745. doi: 10.1371/journal.pone.0309745 (PMC11500959; doi:10.1371/journal.pone.0309745)
Supplement: S1 Table — (PDF) [file pone.0309745.s002.pdf]

S2 Table. Information about each included study

Number	Authors	Publication Year	Source	Study Region	Latitude (centre)	Longitude (centre)	Sample size	Study Period	Theory-driven	Analysis design	Variables	Correlation Coefficient	p-value	Hotels category	Contribution	Data type	Mean energy use	Energy data unit	Energy data type	Data extractor responsible	Date of extraction	Included or not included in meta-analysis
1	Silu Bhochhibhoya, Massimo Pizzol, Francesco Marinello, Raffaele Cavalli	2020	Journal of Cleaner Production	Solukhumbu, Nepal	27.74	86.73	45	2014	Life Cycle Assessment (LCA) and Life Cycle Costing (LCC)	ANOVA and Correlation Analysis	Carbon emissions construction	NA	NA	General	Data is from study 20	Secondary data	10.74	kWh/Person.Night	EUI	R.S.A., T.M., and R.M.U.	27th October to 11th November. 2023	Not used in meta-analysis due duplicated data
1	Silu Bhochhibhoya, Massimo Pizzol, Francesco Marinello, Raffaele Cavalli	2020	Journal of Cleaner Production	Solukhumbu, Nepal	27.74	86.73	45	2014	Life Cycle Assessment (LCA) and Life Cycle Costing (LCC)	ANOVA and Correlation Analysis	Carbon emission operation	NA	NA	General	Data is from study 20	Secondary data	10.74	kWh/Person.Night	EUI	R.S.A., T.M., and R.M.U.	27th October to 11th November. 2023	Not used in meta-analysis due duplicated data
1	Silu Bhochhibhoya, Massimo Pizzol, Francesco Marinello, Raffaele Cavalli	2020	Journal of Cleaner Production	Solukhumbu, Nepal	27.74	86.73	45	2014	Life Cycle Assessment (LCA) and Life Cycle Costing (LCC)	ANOVA and Correlation Analysis	Carbon emissions replacement	NA	NA	General	Data is from study 20	Secondary data	10.74	kWh/Person.Night	EUI	R.S.A., T.M., and R.M.U.	27th October to 11th November. 2023	Not used in meta-analysis due duplicated data
1	Silu Bhochhibhoya, Massimo Pizzol, Francesco Marinello, Raffaele Cavalli	2020	Journal of Cleaner Production	Solukhumbu, Nepal	27.74	86.73	45	2014	Life Cycle Assessment (LCA) and Life Cycle Costing (LCC)	ANOVA and Correlation Analysis	carbon emissions	NA	NA	General	Data is from study 20	Secondary data	10.74	kWh/Person.Night	EUI	R.S.A., T.M., and R.M.U.	27th October to 11th November. 2023	Not used in meta-analysis due duplicated data
1	Silu Bhochhibhoya, Massimo Pizzol, Francesco Marinello, Raffaele Cavalli	2020	Journal of Cleaner Production	Solukhumbu, Nepal	27.74	86.73	45	2014	Life Cycle Assessment (LCA) and Life Cycle Costing (LCC)	ANOVA and Correlation Analysis	Construction cost	NA	NA	General	Data is from study 20	Secondary data	10.74	kWh/Person.Night	EUI	R.S.A., T.M., and R.M.U.	27th October to 11th November. 2023	Not used in meta-analysis due duplicated data
1	Silu Bhochhibhoya, Massimo Pizzol, Francesco Marinello, Raffaele Cavalli	2020	Journal of Cleaner Production	Solukhumbu, Nepal	27.74	86.73	45	2014	Life Cycle Assessment (LCA) and Life Cycle Costing (LCC)	ANOVA and Correlation Analysis	Operation cost	NA	NA	General	Data is from study 20	Secondary data	10.74	kWh/Person.Night	EUI	R.S.A., T.M., and R.M.U.	27th October to 11th November. 2023	Not used in meta-analysis due duplicated data
1	Silu Bhochhibhoya, Massimo Pizzol, Francesco Marinello, Raffaele Cavalli	2020	Journal of Cleaner Production	Solukhumbu, Nepal	27.74	86.73	45	2014	Life Cycle Assessment (LCA) and Life Cycle Costing (LCC)	ANOVA and Correlation Analysis	Maintenance cost replacement	NA	NA	General	Data is from study 20	Secondary data	10.74	kWh/Person.Night	EUI	R.S.A., T.M., and R.M.U.	27th October to 11th November. 2023	Not used in meta-analysis due duplicated data
1	Silu Bhochhibhoya, Massimo Pizzol, Francesco Marinello, Raffaele Cavalli	2020	Journal of Cleaner Production	Solukhumbu, Nepal	27.74	86.73	45	2014	Life Cycle Assessment (LCA) and Life Cycle Costing (LCC)	ANOVA and Correlation Analysis	custo total	NA	NA	General	Data is from study 20	Secondary data	10.74	kWh/Person.Night	EUI	R.S.A., T.M., and R.M.U.	27th October to 11th November. 2023	Not used in meta-analysis due duplicated data
1	Silu Bhochhibhoya, Massimo Pizzol, Francesco Marinello, Raffaele Cavalli	2020	Journal of Cleaner Production	Solukhumbu, Nepal	27.74	86.73	45	2014	Life Cycle Assessment (LCA) and Life Cycle Costing (LCC)	ANOVA and Correlation Analysis	Loss rate	NA	NA	General	Data is from study 20	Secondary data	10.74	kWh/Person.Night	EUI	R.S.A., T.M., and R.M.U.	27th October to 11th November. 2023	Not used in meta-analysis due duplicated data
1	Silu Bhochhibhoya, Massimo Pizzol, Francesco Marinello, Raffaele Cavalli	2020	Journal of Cleaner Production	Solukhumbu, Nepal	27.74	86.73	45	2014	Life Cycle Assessment (LCA) and Life Cycle Costing (LCC)	ANOVA and Correlation Analysis	Tourist number	NA	NA	General	Data is from study 20	Secondary data	10.74	kWh/Person.Night	EUI	R.S.A., T.M., and R.M.U.	27th October to 11th November. 2023	Not used in meta-analysis due duplicated data
1	Silu Bhochhibhoya, Massimo Pizzol, Francesco Marinello, Raffaele Cavalli	2020	Journal of Cleaner Production	Solukhumbu, Nepal	27.74	86.73	45	2014	Life Cycle Assessment (LCA) and Life Cycle Costing (LCC)	ANOVA and Correlation Analysis	Building area	NA	NA	General	Data is from study 20	Secondary data	10.74	kWh/Person.Night	EUI	R.S.A., T.M., and R.M.U.	27th October to 11th November. 2023	Not used in meta-analysis due duplicated data
2	Hyerim Yoon, David Sauri, Antonio Rico	2021	Current Issues in Tourism	Benidorm, Spain	38.54	-0.13	12	2017 - 2018	Water-energy Nexus (WEN)	Descriptive Statistics and Spearman correlations	water use	0.85	<0,01	General	Main Study	Primary data	536.84	kWh/m^2	EUI	R.S.A., T.M., and R.M.U.	27th October to 11th November. 2023	Used in meta-analysis
2	Hyerim Yoon, David Sauri, Antonio Rico	2021	Current Issues in Tourism	Benidorm, Spain	38.54	-0.13	12	2017 - 2018	Water-energy Nexus (WEN)	Descriptive Statistics and Spearman correlations	restaurants	0.65	<0,05	General	Main Study	Primary data	536.84	kWh/m^2	EUI	R.S.A., T.M., and R.M.U.	27th October to 11th November. 2023	Not used in meta-analysis due number of effect sizes less than 3
2	Hyerim Yoon, David Sauri, Antonio Rico	2021	Current Issues in Tourism	Benidorm, Spain	38.54	-0.13	12	2017 - 2018	Water-energy Nexus (WEN)	Descriptive Statistics and Spearman correlations	Garden area	0.23	>0,10	General	Main Study	Primary data	536.84	kWh/m^2	EUI	R.S.A., T.M., and R.M.U.	27th October to 11th November. 2023	Not used in meta-analysis due number of effect sizes less than 3
2	Hyerim Yoon, David Sauri, Antonio Rico	2021	Current Issues in Tourism	Benidorm, Spain	38.54	-0.13	12	2017 - 2018	Water-energy Nexus (WEN)	Descriptive Statistics and Spearman correlations	rank of overnight	0.72	<0,01	General	Main Study	Primary data	536.84	kWh/m^2	EUI	R.S.A., T.M., and R.M.U.	27th October to 11th November. 2023	Not used in meta-analysis due number of effect sizes less than 3
2	Hyerim Yoon, David Sauri, Antonio Rico	2021	Current Issues in Tourism	Benidorm, Spain	38.54	-0.13	12	2017 - 2018	Water-energy Nexus (WEN)	Descriptive Statistics and Spearman correlations	Category (star level)	0.19	>0,10	General	Main Study	Primary data	536.84	kWh/m^2	EUI	R.S.A., T.M., and R.M.U.	27th October to 11th November. 2023	Used in meta-analysis
3	Rodrigo Schons Arenhart, Adriano Mendonça Souza, Roselaine Ruviaro Zanini	2022	Sustainability	Worldwide	NA	NA	45	2019	Descriptive study	correlation and regression analyses	carbon emissions	0.94	<0,05	General	Main Study	Secondary data	101	kWh/occupied room	EUI	R.S.A., T.M., and R.M.U.	27th October to 11th November. 2023	Used in meta-analysis
3	Rodrigo Schons Arenhart, Adriano Mendonça Souza, Roselaine Ruviaro Zanini	2022	Sustainability	Worldwide	NA	NA	45	2019	Descriptive study	correlation and regression analyses	RevPAR	0.84	<0,05	General	Main Study	Secondary data	101	kWh/occupied room	EUI	R.S.A., T.M., and R.M.U.	27th October to 11th November. 2023	Used in meta-analysis
3	Rodrigo Schons Arenhart, Adriano Mendonça Souza, Roselaine Ruviaro Zanini	2022	Sustainability	Worldwide	NA	NA	45	2019	Descriptive study	correlation and regression analyses	Net return	0.36	<0,05	General	Main Study	Secondary data	101	kWh/occupied room	EUI	R.S.A., T.M., and R.M.U.	27th October to 11th November. 2023	Not used in meta-analysis due number of effect sizes less than 3
3	Rodrigo Schons Arenhart, Adriano Mendonça Souza, Roselaine Ruviaro Zanini	2022	Sustainability	Worldwide	NA	NA	45	2019	Descriptive study	correlation and regression analyses	water use	0.54	<0,05	General	Main Study	Secondary data	101	kWh/occupied room	EUI	R.S.A., T.M., and R.M.U.	27th October to 11th November. 2023	Used in meta-analysis
4	Anh Tuan Nguyen, David Rockwood	2019	Journal of Green Building	Danang, Vietnam	16.05	108.15	16	2015	Descriptive study	Descriptive Statistics, correlations, Simulation, and energy use indicator	floor area	0.90	<0,05	1.5	Second use in the same study	Primary data	55820	kWh/year	raw	R.S.A., T.M., and R.M.U.	27th October to 11th November. 2023	Not used in meta-analysis due sample number restriction
4	Anh Tuan Nguyen, David Rockwood	2019	Journal of Green Building	Danang, Vietnam	16.05	108.15	21	2015	Descriptive study	Descriptive Statistics, correlations, Simulation, and energy use indicator	floor area	0.80	<0,05	3	Second use in the same study	Primary data	300999	kWh/year	raw	R.S.A., T.M., and R.M.U.	27th October to 11th November. 2023	Used in meta-analysis
4	Anh Tuan Nguyen, David Rockwood	2019	Journal of Green Building	Danang, Vietnam	16.05	108.15	8	2015	Descriptive study	Descriptive Statistics, correlations, Simulation, and energy use indicator	floor area	0.71	<0,05	4	Second use in the same study	Primary data	1325203	kWh/year	raw	R.S.A., T.M., and R.M.U.	27th October to 11th November. 2023	Not used in meta-analysis due sample number restriction
4	Anh Tuan Nguyen, David Rockwood	2019	Journal of Green Building	Danang, Vietnam	16.05	108.15	5	2015	Descriptive study	Descriptive Statistics, correlations, Simulation, and energy use indicator	floor area	0.98	<0,05	5	Second use in the same study	Primary data	12647491	kWh/year	raw	R.S.A., T.M., and R.M.U.	27th October to 11th November. 2023	Not used in meta-analysis due sample number restriction
4	Anh Tuan Nguyen, David Rockwood	2019	Journal of Green Building	Danang, Vietnam	16.05	108.15	45	2015	Descriptive study	Descriptive Statistics, correlations, Simulation, and energy use indicator	floor area	0.88	<0,05	General	Main Study	Primary data	434460	kWh/year	raw	R.S.A., T.M., and R.M.U.	27th October to 11th November. 2023	Used in meta-analysis
4	Anh Tuan Nguyen, David Rockwood	2019	Journal of Green Building	Danang, Vietnam	16.05	108.15	50	2015	Descriptive study	Descriptive Statistics, correlations, Simulation, and energy use indicator	Occupancy rate	0.00	>0,05	General	Main Study	Primary data	87.4	kWh/m^2.year	EUI	R.S.A., T.M., and R.M.U.	27th October to 11th November. 2023	Used in meta-analysis
5	Joseph H. K. Lai	2016	International Journal of Hospitality Management	Hong Kong	22.4	114.15	30	2013	Descriptive study	Descriptive statistics, benchmarking charts and Pearson correlations	Building age	0.11	>0,10	4.5	Main Study	Primary data	19976	MWh/year	raw	R.S.A., T.M., and R.M.U.	27th October to 11th November. 2023	Used in meta-analysis
5	Joseph H. K. Lai	2016	International Journal of Hospitality Management	Hong Kong	22.4	114.15	30	2013	Descriptive study	Descriptive statistics, benchmarking charts and Pearson correlations	floor area	0.55	<0,01	4.5	Main Study	Primary data	19976	MWh/year	raw	R.S.A., T.M., and R.M.U.	27th October to 11th November. 2023	Used in meta-analysis
5	Joseph H. K. Lai	2016	International Journal of Hospitality Management	Hong Kong	22.4	114.15	30	2013	Descriptive study	Descriptive statistics, benchmarking charts and Pearson correlations	Guestrooms	0.16	>0,10	4.5	Main Study	Primary data	19976	MWh/year	raw	R.S.A., T.M., and R.M.U.	27th October to 11th November. 2023	Used in meta-analysis
5	Joseph H. K. Lai	2016	International Journal of Hospitality Management	Hong Kong	22.4	114.15	30	2013	Descriptive study	Descriptive statistics, benchmarking charts and Pearson correlations	Occupancy rate	-0.39	<0,05	4.5	Main Study	Primary data	19976	MWh/year	raw	R.S.A., T.M., and R.M.U.	27th October to 11th November. 2023	Used in meta-analysis
5	Joseph H. K. Lai	2016	International Journal of Hospitality Management	Hong Kong	22.4	114.15	30	2013	Descriptive study	Descriptive statistics, benchmarking charts and Pearson correlations	floor area	-0.15	>0,10	4.5	Second use in the same study	Primary data	474.55	kWh/m^2	EUI	R.S.A., T.M., and R.M.U.	27th October to 11th November. 2023	Used in meta-analysis
5	Joseph H. K. Lai	2016	International Journal of Hospitality Management	Hong Kong	22.4	114.15	30	2013	Descriptive study	Descriptive statistics, benchmarking charts and Pearson correlations	Guestrooms	-0.20	>0,10	4.5	Second use in the same study	Primary data	474.55	kWh/m^2	EUI	R.S.A., T.M., and R.M.U.	27th October to 11th November. 2023	Used in meta-analysis
5	Joseph H. K. Lai	2016	International Journal of Hospitality Management	Hong Kong	22.4	114.15	30	2013	Descriptive study	Descriptive statistics, benchmarking charts and Pearson correlations	Occupancy rate	-0.37	<0,05	4.5	Second use in the same study	Primary data	474.55	kWh/m^2	EUI	R.S.A., T.M., and R.M.U.	27th October to 11th November. 2023	Used in meta-analysis
6	Wu Xuchao, Rajagopalan Priyadarsini, Lee Siew Eang	2010	Energy Policy	Singapore	1.37	103.81	29	2004	Descriptive study	Descriptive statistics, regression techniques, consumption indicators and benchmarking	floor area	NA	NA	General	Data is from study 16	Secondary data	427	kWh/m^2	EUI	R.S.A., T.M., and R.M.U.	27th October to 11th November. 2023	Not used in meta-analysis due duplicated data
6	Wu Xuchao, Rajagopalan Priyadarsini, Lee Siew Eang	2010	Energy Policy	Singapore	1.37	103.81	29	2004	Descriptive study	Descriptive statistics, regression techniques, consumption indicators and benchmarking	number of floors	NA	NA	General	Data is from study 16	Secondary data	427	kWh/m^2	EUI	R.S.A., T.M., and R.M.U.	27th October to 11th November. 2023	Not used in meta-analysis due duplicated data
6	Wu Xuchao, Rajagopalan Priyadarsini, Lee Siew Eang	2010	Energy Policy	Singapore	1.37	103.81	29	2004	Descriptive study	Descriptive statistics, regression techniques, consumption indicators and benchmarking	Guestrooms	NA	NA	General	Data is from study 16	Secondary data	427	kWh/m^2	EUI	R.S.A., T.M., and R.M.U.	27th October to 11th November. 2023	Not used in meta-analysis due duplicated data
6	Wu Xuchao, Rajagopalan Priyadarsini, Lee Siew Eang	2010	Energy Policy	Singapore	1.37	103.81	29	2004	Descriptive study	Descriptive statistics, regression techniques, consumption indicators and benchmarking	area of guestroom	NA	NA	General	Data is from study 16	Secondary data	427	kWh/m^2	EUI	R.S.A., T.M., and R.M.U.	27th October to 11th November. 2023	Not used in meta-analysis due duplicated data

6	Wu Xuchao, Rajagopalan Priyadarsini, Lee Siew Eang	2010	Energy Policy	Singapore	1.37	103.81	29	2004	Descriptive study	Descriptive statistics, regression techniques, consumption indicators and benchmarking	building age	NA	NA	General	Data is from study 16	Secondary data	427	kWh/m^2	EUI	R.S.A., T.M., and R.M.U.	27th October to 11th November. 2023	Not used in meta-analysis due duplicated data
6	Wu Xuchao, Rajagopalan Priyadarsini, Lee Siew Eang	2010	Energy Policy	Singapore	1.37	103.81	29	2004	Descriptive study	Descriptive statistics, regression techniques, consumption indicators and benchmarking	years after retrofit	NA	NA	General	Data is from study 16	Secondary data	427	kWh/m^2	EUI	R.S.A., T.M., and R.M.U.	27th October to 11th November. 2023	Not used in meta-analysis due duplicated data
6	Wu Xuchao, Rajagopalan Priyadarsini, Lee Siew Eang	2010	Energy Policy	Singapore	1.37	103.81	29	2004	Descriptive study	Descriptive statistics, regression techniques, consumption indicators and benchmarking	workers per shift	NA	NA	General	Data is from study 16	Secondary data	427	kWh/m^2	EUI	R.S.A., T.M., and R.M.U.	27th October to 11th November. 2023	Not used in meta-analysis due duplicated data
6	Wu Xuchao, Rajagopalan Priyadarsini, Lee Siew Eang	2010	Energy Policy	Singapore	1.37	103.81	29	2004	Descriptive study	Descriptive statistics, regression techniques, consumption indicators and benchmarking	worker density	NA	NA	General	Data is from study 16	Secondary data	427	kWh/m^2	EUI	R.S.A., T.M., and R.M.U.	27th October to 11th November. 2023	Not used in meta-analysis due duplicated data
6	Wu Xuchao, Rajagopalan Priyadarsini, Lee Siew Eang	2010	Energy Policy	Singapore	1.37	103.81	29	2004	Descriptive study	Descriptive statistics, regression techniques, consumption indicators and benchmarking	energy audit	NA	NA	General	Data is from study 16	Secondary data	427	kWh/m^2	EUI	R.S.A., T.M., and R.M.U.	27th October to 11th November. 2023	Not used in meta-analysis due duplicated data
6	Wu Xuchao, Rajagopalan Priyadarsini, Lee Siew Eang	2010	Energy Policy	Singapore	1.37	103.81	29	2004	Descriptive study	Descriptive statistics, regression techniques, consumption indicators and benchmarking	laundry	NA	NA	General	Data is from study 16	Secondary data	427	kWh/m^2	EUI	R.S.A., T.M., and R.M.U.	27th October to 11th November. 2023	Not used in meta-analysis due duplicated data
6	Wu Xuchao, Rajagopalan Priyadarsini, Lee Siew Eang	2010	Energy Policy	Singapore	1.37	103.81	29	2004	Descriptive study	Descriptive statistics, regression techniques, consumption indicators and benchmarking	5 star	NA	NA	General	Data is from study 16	Secondary data	427	kWh/m^2	EUI	R.S.A., T.M., and R.M.U.	27th October to 11th November. 2023	Not used in meta-analysis due duplicated data
6	Wu Xuchao, Rajagopalan Priyadarsini, Lee Siew Eang	2010	Energy Policy	Singapore	1.37	103.81	29	2004	Descriptive study	Descriptive statistics, regression techniques, consumption indicators and benchmarking	4 star	NA	NA	General	Data is from study 16	Secondary data	427	kWh/m^2	EUI	R.S.A., T.M., and R.M.U.	27th October to 11th November. 2023	Not used in meta-analysis due duplicated data
6	Wu Xuchao, Rajagopalan Priyadarsini, Lee Siew Eang	2010	Energy Policy	Singapore	1.37	103.81	29	2004	Descriptive study	Descriptive statistics, regression techniques, consumption indicators and benchmarking	3 star	NA	NA	General	Data is from study 16	Secondary data	427	kWh/m^2	EUI	R.S.A., T.M., and R.M.U.	27th October to 11th November. 2023	Not used in meta-analysis due duplicated data
6	Wu Xuchao, Rajagopalan Priyadarsini, Lee Siew Eang	2010	Energy Policy	Singapore	1.37	103.81	29	2004	Descriptive study	Descriptive statistics, regression techniques, consumption indicators and benchmarking	Occupancy rate	NA	NA	General	Data is from study 16	Secondary data	427	kWh/m^2	EUI	R.S.A., T.M., and R.M.U.	27th October to 11th November. 2023	Not used in meta-analysis due duplicated data
7	P. O. Oluseyi, O. M. Babatunde, O. A. Babatunde	2016	Energy and Buildings	Lagos, Nigeria	6.55	3.31	28	2014	Descriptive study	descriptive statistics, correlations analysis, linear regression and consumption indicators	floor area	0.77	<0,01	General	Main Study	Primary data	NI	MWh	raw	R.S.A., T.M., and R.M.U.	27th October to 11th November. 2023	Used in meta-analysis
7	P. O. Oluseyi, O. M. Babatunde, O. A. Babatunde	2016	Energy and Buildings	Lagos, Nigeria	6.55	3.31	28	2014	Descriptive study	descriptive statistics, correlations analysis, linear regression and consumption indicators	building age	0.26	>0,01	General	Main Study	Primary data	NI	MWh	raw	R.S.A., T.M., and R.M.U.	27th October to 11th November. 2023	Used in meta-analysis
7	P. O. Oluseyi, O. M. Babatunde, O. A. Babatunde	2016	Energy and Buildings	Lagos, Nigeria	6.55	3.31	28	2014	Descriptive study	descriptive statistics, correlations analysis, linear regression and consumption indicators	Guestrooms	0.69	<0,01	General	Main Study	Primary data	NI	MWh	raw	R.S.A., T.M., and R.M.U.	27th October to 11th November. 2023	Used in meta-analysis
7	P. O. Oluseyi, O. M. Babatunde, O. A. Babatunde	2016	Energy and Buildings	Lagos, Nigeria	6.55	3.31	28	2014	Descriptive study	descriptive statistics, correlations analysis, linear regression and consumption indicators	Occupancy rate	-0.13	>0,01	General	Main Study	Primary data	NI	MWh	raw	R.S.A., T.M., and R.M.U.	27th October to 11th November. 2023	Used in meta-analysis
7	P. O. Oluseyi, O. M. Babatunde, O. A. Babatunde	2016	Energy and Buildings	Lagos, Nigeria	6.55	3.31	28	2014	Descriptive study	descriptive statistics, correlations analysis, linear regression and consumption indicators	guest-nights	0.66	<0,01	General	Main Study	Primary data	NI	MWh	raw	R.S.A., T.M., and R.M.U.	27th October to 11th November. 2023	Used in meta-analysis
7	P. O. Oluseyi, O. M. Babatunde, O. A. Babatunde	2016	Energy and Buildings	Lagos, Nigeria	6.55	3.31	28	2014	Descriptive study	descriptive statistics, correlations analysis, linear regression and consumption indicators	number of employees	0.76	<0,01	General	Main Study	Primary data	NI	MWh	raw	R.S.A., T.M., and R.M.U.	27th October to 11th November. 2023	Used in meta-analysis
7	P. O. Oluseyi, O. M. Babatunde, O. A. Babatunde	2016	Energy and Buildings	Lagos, Nigeria	6.55	3.31	28	2014	Descriptive study	descriptive statistics, correlations analysis, linear regression and consumption indicators	floor area	0.42	<0,05	General	Second use in the same study	Primary data	41.281	MWh/guestroom	EUI	R.S.A., T.M., and R.M.U.	27th October to 11th November. 2023	Not used in meta-analysis due duplicated data
7	P. O. Oluseyi, O. M. Babatunde, O. A. Babatunde	2016	Energy and Buildings	Lagos, Nigeria	6.55	3.31	28	2014	Descriptive study	descriptive statistics, correlations analysis, linear regression and consumption indicators	carbon emissions	0.56	<0,01	General	Main Study	Primary data	41.281	MWh/guestroom	EUI	R.S.A., T.M., and R.M.U.	27th October to 11th November. 2023	Used in meta-analysis
7	P. O. Oluseyi, O. M. Babatunde, O. A. Babatunde	2016	Energy and Buildings	Lagos, Nigeria	6.55	3.31	28	2014	Descriptive study	descriptive statistics, correlations analysis, linear regression and consumption indicators	floor area	-0.07	>0,10	General	Second use in the same study	Primary data	265.95	kWh/m^2	EUI	R.S.A., T.M., and R.M.U.	27th October to 11th November. 2023	Used in meta-analysis
7	P. O. Oluseyi, O. M. Babatunde, O. A. Babatunde	2016	Energy and Buildings	Lagos, Nigeria	6.55	3.31	28	2014	Descriptive study	descriptive statistics, correlations analysis, linear regression and consumption indicators	Guestrooms	0.05	>0,10	General	Second use in the same study	Primary data	265.95	kWh/m^2	EUI	R.S.A., T.M., and R.M.U.	27th October to 11th November. 2023	Used in meta-analysis
7	P. O. Oluseyi, O. M. Babatunde, O. A. Babatunde	2016	Energy and Buildings	Lagos, Nigeria	6.55	3.31	28	2014	Descriptive study	descriptive statistics, correlations analysis, linear regression and consumption indicators	Occupancy rate	0.04	>0,10	General	Second use in the same study	Primary data	265.95	kWh/m^2	EUI	R.S.A., T.M., and R.M.U.	27th October to 11th November. 2023	Used in meta-analysis
8	Dunia E. Santiago	2021	International Journal of Low-Carbon Technologies	Gran Canaria	28.01	-15.59	6	2019-2020	Descriptive study	descriptive statistics, correlation analysis, linear regression	Cold water temperature	-0.33	<0,05	General	Main Study	Primary data	200000	kWh	raw	R.S.A., T.M., and R.M.U.	27th October to 11th November. 2023	Not used in meta-analysis due number of effect sizes less than 3
8	Dunia E. Santiago	2021	International Journal of Low-Carbon Technologies	Gran Canaria	28.01	-15.59	6	2019-2020	Descriptive study	descriptive statistics, correlation analysis, linear regression	Category (star level)	0.40	<0,05	General	Main Study	Primary data	200000	kWh	raw	R.S.A., T.M., and R.M.U.	27th October to 11th November. 2023	Used in meta-analysis
8	Dunia E. Santiago	2021	International Journal of Low-Carbon Technologies	Gran Canaria	28.01	-15.59	6	2019-2020	Descriptive study	descriptive statistics, correlation analysis, linear regression	Guestrooms	0.79	<0,05	General	Main Study	Primary data	200000	kWh	raw	R.S.A., T.M., and R.M.U.	27th October to 11th November. 2023	Used in meta-analysis
8	Dunia E. Santiago	2021	International Journal of Low-Carbon Technologies	Gran Canaria	28.01	-15.59	6	2019-2020	Descriptive study	descriptive statistics, correlation analysis, linear regression	number of floors	-0.29	<0,05	General	Main Study	Primary data	200000	kWh	raw	R.S.A., T.M., and R.M.U.	27th October to 11th November. 2023	Used in meta-analysis
8	Dunia E. Santiago	2021	International Journal of Low-Carbon Technologies	Gran Canaria	28.01	-15.59	6	2019-2020	Descriptive study	descriptive statistics, correlation analysis, linear regression	years after retrofit	-0.29	>0,05	General	Main Study	Primary data	200000	kWh	raw	R.S.A., T.M., and R.M.U.	27th October to 11th November. 2023	Used in meta-analysis
8	Dunia E. Santiago	2021	International Journal of Low-Carbon Technologies	Gran Canaria	28.01	-15.59	6	2019-2020	Descriptive study	descriptive statistics, correlation analysis, linear regression	floor area	0.85	<0,05	General	Main Study	Primary data	200000	kWh	raw	R.S.A., T.M., and R.M.U.	27th October to 11th November. 2023	Used in meta-analysis
8	Dunia E. Santiago	2021	International Journal of Low-Carbon Technologies	Gran Canaria	28.01	-15.59	6	2019-2020	Descriptive study	descriptive statistics, correlation analysis, linear regression	pools (volume)	0.65	<0,05	General	Main Study	Primary data	200000	kWh	raw	R.S.A., T.M., and R.M.U.	27th October to 11th November. 2023	Not used in meta-analysis due number of effect sizes less than 3
8	Dunia E. Santiago	2021	International Journal of Low-Carbon Technologies	Gran Canaria	28.01	-15.59	6	2019-2020	Descriptive study	descriptive statistics, correlation analysis, linear regression	pools (area)	0.75	<0,05	General	Main Study	Primary data	200000	kWh	raw	R.S.A., T.M., and R.M.U.	27th October to 11th November. 2023	Not used in meta-analysis due number of effect sizes less than 3
8	Dunia E. Santiago	2021	International Journal of Low-Carbon Technologies	Gran Canaria	28.01	-15.59	6	2019-2020	Descriptive study	descriptive statistics, correlation analysis, linear regression	pools (Temperature)	-0.40	<0,05	General	Main Study	Primary data	200000	kWh	raw	R.S.A., T.M., and R.M.U.	27th October to 11th November. 2023	Not used in meta-analysis due number of effect sizes less than 3
8	Dunia E. Santiago	2021	International Journal of Low-Carbon Technologies	Gran Canaria	28.01	-15.59	6	2019-2020	Descriptive study	descriptive statistics, correlation analysis, linear regression	aircon*	0.53	<0,05	General	Main Study	Primary data	200000	kWh	raw	R.S.A., T.M., and R.M.U.	27th October to 11th November. 2023	Not used in meta-analysis due number of effect sizes less than 3
8	Dunia E. Santiago	2021	International Journal of Low-Carbon Technologies	Gran Canaria	28.01	-15.59	6	2019-2020	Descriptive study	descriptive statistics, correlation analysis, linear regression	Spa	-0.29	<0,05	General	Main Study	Primary data	200000	kWh	raw	R.S.A., T.M., and R.M.U.	27th October to 11th November. 2023	Not used in meta-analysis due number of effect sizes less than 3
8	Dunia E. Santiago	2021	International Journal of Low-Carbon Technologies	Gran Canaria	28.01	-15.59	6	2019-2020	Descriptive study	descriptive statistics, correlation analysis, linear regression	Kitchenette in room	-0.56	<0,05	General	Main Study	Primary data	200000	kWh	raw	R.S.A., T.M., and R.M.U.	27th October to 11th November. 2023	Not used in meta-analysis due number of effect sizes less than 3
8	Dunia E. Santiago	2021	International Journal of Low-Carbon Technologies	Gran Canaria	28.01	-15.59	6	2019-2020	Descriptive study	descriptive statistics, correlation analysis, linear regression	travelife certified	0.04	>0,05	General	Main Study	Primary data	200000	kWh	raw	R.S.A., T.M., and R.M.U.	27th October to 11th November. 2023	Not used in meta-analysis due number of effect sizes less than 3
8	Dunia E. Santiago	2021	International Journal of Low-Carbon Technologies	Gran Canaria	28.01	-15.59	6	2019-2020	Descriptive study	descriptive statistics, correlation analysis, linear regression	renewable energy	-0.27	<0,05	General	Main Study	Primary data	200000	kWh	raw	R.S.A., T.M., and R.M.U.	27th October to 11th November. 2023	Not used in meta-analysis due number of effect sizes less than 3
8	Dunia E. Santiago	2021	International Journal of Low-Carbon Technologies	Gran Canaria	28.01	-15.59	6	2019-2020	Descriptive study	descriptive statistics, correlation analysis, linear regression	food covers	0.87	<0,05	General	Main Study	Primary data	200000	kWh	raw	R.S.A., T.M., and R.M.U.	27th October to 11th November. 2023	Used in meta-analysis

8	Dunia E. Santiago	2021	International Journal of Low-Carbon Technologies	Gran Canaria	28.01	-15.59	6	2019-2020	Descriptive study	descriptive statistics, correlation analysis, linear regression	ADR	0.61	<0,05	General	Main Study	Primary data	200000	kWh	raw	R.S.A., T.M., and R.M.U.	27th October to 11th November. 2023	Not used in meta-analysis due number of effect sizes less than 3
8	Dunia E. Santiago	2021	International Journal of Low-Carbon Technologies	Gran Canaria	28.01	-15.59	6	2019-2020	Descriptive study	descriptive statistics, correlation analysis, linear regression	guest per room	0.54	<0,05	General	Main Study	Primary data	200000	kWh	raw	R.S.A., T.M., and R.M.U.	27th October to 11th November. 2023	Not used in meta-analysis due number of effect sizes less than 3
8	Dunia E. Santiago	2021	International Journal of Low-Carbon Technologies	Gran Canaria	28.01	-15.59	6	2019-2020	Descriptive study	descriptive statistics, correlation analysis, linear regression	RevPAR	0.64	<0,05	General	Main Study	Primary data	200000	kWh	raw	R.S.A., T.M., and R.M.U.	27th October to 11th November. 2023	Used in meta-analysis
8	Dunia E. Santiago	2021	International Journal of Low-Carbon Technologies	Gran Canaria	28.01	-15.59	6	2019-2020	Descriptive study	descriptive statistics, correlation analysis, linear regression	GRI*	-0.15	>0,05	General	Main Study	Primary data	200000	kWh	raw	R.S.A., T.M., and R.M.U.	27th October to 11th November. 2023	Not used in meta-analysis due number of effect sizes less than 3
8	Dunia E. Santiago	2021	International Journal of Low-Carbon Technologies	Gran Canaria	28.01	-15.59	6	2019-2020	Descriptive study	descriptive statistics, correlation analysis, linear regression	number of employees	0.79	<0,05	General	Main Study	Primary data	200000	kWh	raw	R.S.A., T.M., and R.M.U.	27th October to 11th November. 2023	Used in meta-analysis
8	Dunia E. Santiago	2021	International Journal of Low-Carbon Technologies	Gran Canaria	28.01	-15.59	6	2019-2020	Descriptive study	descriptive statistics, correlation analysis, linear regression	guest-nights	0.92	<0,05	General	Main Study	Primary data	200000	kWh	raw	R.S.A., T.M., and R.M.U.	27th October to 11th November. 2023	Used in meta-analysis
8	Dunia E. Santiago	2021	International Journal of Low-Carbon Technologies	Gran Canaria	28.01	-15.59	6	2019-2020	Descriptive study	descriptive statistics, correlation analysis, linear regression	number of adult guests	0.91	<0,05	General	Main Study	Primary data	200000	kWh	raw	R.S.A., T.M., and R.M.U.	27th October to 11th November. 2023	Not used in meta-analysis due number of effect sizes less than 3
8	Dunia E. Santiago	2021	International Journal of Low-Carbon Technologies	Gran Canaria	28.01	-15.59	6	2019-2020	Descriptive study	descriptive statistics, correlation analysis, linear regression	number of children guests	0.81	<0,05	General	Main Study	Primary data	200000	kWh	raw	R.S.A., T.M., and R.M.U.	27th October to 11th November. 2023	Not used in meta-analysis due number of effect sizes less than 3
8	Dunia E. Santiago	2021	International Journal of Low-Carbon Technologies	Gran Canaria	28.01	-15.59	6	2019-2020	Descriptive study	descriptive statistics, correlation analysis, linear regression	number of diners	0.95	<0,05	General	Main Study	Primary data	200000	kWh	raw	R.S.A., T.M., and R.M.U.	27th October to 11th November. 2023	Not used in meta-analysis due number of effect sizes less than 3
8	Dunia E. Santiago	2021	International Journal of Low-Carbon Technologies	Gran Canaria	28.01	-15.59	6	2019-2020	Descriptive study	descriptive statistics, correlation analysis, linear regression	number of repairs	0.87	<0,05	General	Main Study	Primary data	200000	kWh	raw	R.S.A., T.M., and R.M.U.	27th October to 11th November. 2023	Not used in meta-analysis due number of effect sizes less than 3
8	Dunia E. Santiago	2021	International Journal of Low-Carbon Technologies	Gran Canaria	28.01	-15.59	6	2019-2020	Descriptive study	descriptive statistics, correlation analysis, linear regression	maintenance cost	0.68	<0,05	General	Main Study	Primary data	200000	kWh	raw	R.S.A., T.M., and R.M.U.	27th October to 11th November. 2023	Not used in meta-analysis due number of effect sizes less than 3
8	Dunia E. Santiago	2021	International Journal of Low-Carbon Technologies	Gran Canaria	28.01	-15.59	6	2019-2020	Descriptive study	descriptive statistics, correlation analysis, linear regression	water use	0.94	<0,05	General	Main Study	Primary data	200000	kWh	raw	R.S.A., T.M., and R.M.U.	27th October to 11th November. 2023	Used in meta-analysis
8	Dunia E. Santiago	2021	International Journal of Low-Carbon Technologies	Gran Canaria	28.01	-15.59	6	2019-2020	Descriptive study	descriptive statistics, correlation analysis, linear regression	refrigerants reposition	0.41	<0,05	General	Main Study	Primary data	200000	kWh	raw	R.S.A., T.M., and R.M.U.	27th October to 11th November. 2023	Not used in meta-analysis due number of effect sizes less than 3
8	Dunia E. Santiago	2021	International Journal of Low-Carbon Technologies	Gran Canaria	28.01	-15.59	6	2019-2020	Descriptive study	descriptive statistics, correlation analysis, linear regression	recycled kitchen oil	0.84	<0,05	General	Main Study	Primary data	200000	kWh	raw	R.S.A., T.M., and R.M.U.	27th October to 11th November. 2023	Not used in meta-analysis due number of effect sizes less than 3
8	Dunia E. Santiago	2021	International Journal of Low-Carbon Technologies	Gran Canaria	28.01	-15.59	6	2019-2020	Descriptive study	descriptive statistics, correlation analysis, linear regression	recycled cardboard	0.81	<0,05	General	Main Study	Primary data	200000	kWh	raw	R.S.A., T.M., and R.M.U.	27th October to 11th November. 2023	Not used in meta-analysis due number of effect sizes less than 3
8	Dunia E. Santiago	2021	International Journal of Low-Carbon Technologies	Gran Canaria	28.01	-15.59	6	2019-2020	Descriptive study	descriptive statistics, correlation analysis, linear regression	Occupancy rate	0.39	<0,05	General	Main Study	Primary data	200000	kWh	raw	R.S.A., T.M., and R.M.U.	27th October to 11th November. 2023	Used in meta-analysis
9	Wilco W. Chan, Barry L. Mak	2004	Journal of Sustainable Tourism	Hong Kong	22.4	114.15	10	1994-1996	Descriptive study (Considering only the Oil Diesel for energy use in water and steam)	Linear Regression	floor area	0.95	<0,05	General	Third use data from study 24	Secondary data	35000	MJ	raw	R.S.A., T.M., and R.M.U.	27th October to 11th November. 2023	Used in meta-analysis
10	Wilco W. Chan, Joseph C. Lam	2002	Hospitality Management	Hong Kong	22.4	114.15	17	1994-1996	descriptive study (considering only the electricity source for energy use)	Linear Regression	floor area	0.94	<0,05	General	second use data from study 24	Secondary data	10000	MWh	raw	R.S.A., T.M., and R.M.U.	27th October to 11th November. 2023	Used in meta-analysis
10	Wilco W. Chan, Joseph C. Lam	2002	Hospitality Management	Hong Kong	22.4	114.15	17	1994-1996	descriptive study (considering only the electricity source for energy use)	Linear Regression	floor area	-0.37	NI	General	second use data from study 24	Secondary data	342.02	kWh/m^2	EUI	R.S.A., T.M., and R.M.U.	27th October to 11th November. 2023	Used in meta-analysis
10	Wilco W. Chan, Joseph C. Lam	2002	Hospitality Management	Hong Kong	22.4	114.15	17	1994-1996	descriptive study (considering only the electricity source for energy use)	Linear Regression	occupied rooms	0.78	>0,05	General	second use data from study 24	Secondary data	10000	MWh	raw	R.S.A., T.M., and R.M.U.	27th October to 11th November. 2023	Used in meta-analysis
11	Suwajee Tangon, Jaruwan Chontanawat, Siriluk Chiarakorn	2018	Asia-Pacific Journal of Science and Technology	Thailand	13.7	100.6	63	2011	Descriptive study	Linear Regression and consumption indicators	Occupancy rate	-0.25	<0,05	General	Second use in the same study	Primary data	2292.36	kWh/room night	EUI	R.S.A., T.M., and R.M.U.	27th October to 11th November. 2023	Used in meta-analysis
11	Suwajee Tangon, Jaruwan Chontanawat, Siriluk Chiarakorn	2018	Asia-Pacific Journal of Science and Technology	Thailand	13.7	100.6	63	2011	Descriptive study	Linear Regression and consumption indicators	Guestrooms	0.74	<0,01	General	Main Study	Primary data	800000	KWh/month	raw	R.S.A., T.M., and R.M.U.	27th October to 11th November. 2023	Used in meta-analysis
11	Suwajee Tangon, Jaruwan Chontanawat, Siriluk Chiarakorn	2018	Asia-Pacific Journal of Science and Technology	Thailand	13.7	100.6	63	2011	Descriptive study	Linear Regression and consumption indicators	floor area	0.82	<0,01	General	Main Study	Primary data	800000	KWh/month	raw	R.S.A., T.M., and R.M.U.	27th October to 11th November. 2023	Used in meta-analysis
11	Suwajee Tangon, Jaruwan Chontanawat, Siriluk Chiarakorn	2018	Asia-Pacific Journal of Science and Technology	Thailand	13.7	100.6	63	2011	Descriptive study	Linear Regression and consumption indicators	number of floors	0.52	<0,01	General	Main Study	Primary data	800000	KWh/month	raw	R.S.A., T.M., and R.M.U.	27th October to 11th November. 2023	Used in meta-analysis
11	Suwajee Tangon, Jaruwan Chontanawat, Siriluk Chiarakorn	2018	Asia-Pacific Journal of Science and Technology	Thailand	13.7	100.6	63	2011	Descriptive study	Linear Regression and consumption indicators	Bangkok	0.27	<0,05	General	Main Study	Primary data	800000	KWh/month	raw	R.S.A., T.M., and R.M.U.	27th October to 11th November. 2023	Not used in meta-analysis due number of effect sizes less than 3
11	Suwajee Tangon, Jaruwan Chontanawat, Siriluk Chiarakorn	2018	Asia-Pacific Journal of Science and Technology	Thailand	13.7	100.6	63	2011	Descriptive study	Linear Regression and consumption indicators	number of employees	0.80	<0,01	General	Main Study	Primary data	800000	KWh/month	raw	R.S.A., T.M., and R.M.U.	27th October to 11th November. 2023	Used in meta-analysis
11	Suwajee Tangon, Jaruwan Chontanawat, Siriluk Chiarakorn	2018	Asia-Pacific Journal of Science and Technology	Thailand	13.7	100.6	63	2011	Descriptive study	Linear Regression and consumption indicators	guest-nights	0.77	<0,01	General	Main Study	Primary data	800000	KWh/month	raw	R.S.A., T.M., and R.M.U.	27th October to 11th November. 2023	Used in meta-analysis
11	Suwajee Tangon, Jaruwan Chontanawat, Siriluk Chiarakorn	2018	Asia-Pacific Journal of Science and Technology	Thailand	13.7	100.6	63	2011	Descriptive study	Linear Regression and consumption indicators	number of guests	0.77	<0,01	General	Main Study	Primary data	800000	KWh/month	raw	R.S.A., T.M., and R.M.U.	27th October to 11th November. 2023	Used in meta-analysis
11	Suwajee Tangon, Jaruwan Chontanawat, Siriluk Chiarakorn	2018	Asia-Pacific Journal of Science and Technology	Thailand	13.7	100.6	63	2011	Descriptive study	Linear Regression and consumption indicators	worker density	0.62	<0,01	General	Second use in the same study	Primary data	321.84	kWh/m^2.year	EUI	R.S.A., T.M., and R.M.U.	27th October to 11th November. 2023	Used in meta-analysis
12	Francisco Javier Diaz Perez, David Chinarro, Adib Guardiola Mouhaffel, Ricardo Diaz Martin, Rosa Pino Otin	2016	Indian Journal of Science and Technology	Fuerteventura and Lanzarote (Canary Islands)	28.78	-13.78	6	2007-2015	Descriptive study	Regression analysis	Occupancy rate	-0.95	NI	4	Main Study	Primary data	16	KWh/customer.day	EUI	R.S.A., T.M., and R.M.U.	27th October to 11th November. 2023	Used in meta-analysis
13	Yajuan Xin, Shilei Lu, Neng Zhu, Wei Wu	2012	Energy and Buildings	Hainan Province, China	19.22	109.8	19	2009	Energy Consumption Quota	Descriptive statistics, consumption indicators and Pearson Correlations	Occupancy rate	0.03	>0,10	4.5	Main Study	Primary data	6850000	KWh	raw	R.S.A., T.M., and R.M.U.	27th October to 11th November. 2023	Used in meta-analysis
13	Yajuan Xin, Shilei Lu, Neng Zhu, Wei Wu	2012	Energy and Buildings	Hainan Province, China	19.22	109.8	19	2009	Energy Consumption Quota	Descriptive statistics, consumption indicators and Pearson Correlations	Building area	0.88	<0,01	4.5	Main Study	Primary data	6850000	KWh	raw	R.S.A., T.M., and R.M.U.	27th October to 11th November. 2023	Not used in meta-analysis due number of effect sizes less than 3
13	Yajuan Xin, Shilei Lu, Neng Zhu, Wei Wu	2012	Energy and Buildings	Hainan Province, China	19.22	109.8	19	2009	Energy Consumption Quota	Descriptive statistics, consumption indicators and Pearson Correlations	Building age	-0.10	>0,10	4.5	Main Study	Primary data	6850000	KWh	raw	R.S.A., T.M., and R.M.U.	27th October to 11th November. 2023	Used in meta-analysis
13	Yajuan Xin, Shilei Lu, Neng Zhu, Wei Wu	2012	Energy and Buildings	Hainan Province, China	19.22	109.8	19	2009	Energy Consumption Quota	Descriptive statistics, consumption indicators and Pearson Correlations	Guestrooms	0.69	<0,01	4.5	Main Study	Primary data	6850000	KWh	raw	R.S.A., T.M., and R.M.U.	27th October to 11th November. 2023	Used in meta-analysis
13	Yajuan Xin, Shilei Lu, Neng Zhu, Wei Wu	2012	Energy and Buildings	Hainan Province, China	19.22	109.8	19	2009	Energy Consumption Quota	Descriptive statistics, consumption indicators and Pearson Correlations	Equivalent rooms (rooms*occupancy)	0.66	<0,01	4.5	Main Study	Primary data	6850000	KWh	raw	R.S.A., T.M., and R.M.U.	27th October to 11th November. 2023	Not used in meta-analysis due number of effect sizes less than 3
13	Yajuan Xin, Shilei Lu, Neng Zhu, Wei Wu	2012	Energy and Buildings	Hainan Province, China	19.22	109.8	19	2009	Energy Consumption Quota	Descriptive statistics, consumption indicators and Pearson Correlations	Guestrooms	0.07	>0,10	4.5	Second use in the same study	Primary data	70	KWh/m^2	EUI	R.S.A., T.M., and R.M.U.	27th October to 11th November. 2023	Used in meta-analysis
13	Yajuan Xin, Shilei Lu, Neng Zhu, Wei Wu	2012	Energy and Buildings	Hainan Province, China	19.22	109.8	19	2009	Energy Consumption Quota	Descriptive statistics, consumption indicators and Pearson Correlations	Occupancy rate	0.36	>0,10	4.5	Second use in the same study	Primary data	70	KWh/m^2	EUI	R.S.A., T.M., and R.M.U.	27th October to 11th November. 2023	Used in meta-analysis
14	Mingfang Tang, Xiao Fu, Huiming Cao, Yuan Shen, Hongbing Deng, Gang Wu	2016	Sustainability	Lijiang, China	26.87	100.24	24	2012	Descriptive study	Descriptive statistics and Pearson Correlations and Regression Analysis and consumption indicators	floor area	0.44	<0,05	General	Main Study	Primary data	110	kWh/m^2	EUI	R.S.A., T.M., and R.M.U.	27th October to 11th November. 2023	Used in meta-analysis
14	Mingfang Tang, Xiao Fu, Huiming Cao, Yuan Shen, Hongbing Deng, Gang Wu	2016	Sustainability	Lijiang, China	26.87	100.24	24	2012	Descriptive study	Descriptive statistics and Pearson Correlations and Regression Analysis and consumption indicators	Guestrooms	0.60	<0,01	General	Main Study	Primary data	110	kWh/m^2	EUI	R.S.A., T.M., and R.M.U.	27th October to 11th November. 2023	Used in meta-analysis

14	Mingfang Tang, Xiao Fu, Huiming Cao, Yuan Shen, Hongbing Deng, Gang Wu	2016	Sustainability	Lijiang, China	26.87	100.24	24	2012	Descriptive study	Descriptive statistics and Pearson Correlations and Regression Analysis and consumption indicators	Category (star level)	0.76	<0,01	General	Main Study	Primary data	110	kWh/m^2	EUI	R.S.A., T.M., and R.M.U.	27th October to 11th November. 2023	Used in meta-analysis
14	Mingfang Tang, Xiao Fu, Huiming Cao, Yuan Shen, Hongbing Deng, Gang Wu	2016	Sustainability	Lijiang, China	26.87	100.24	24	2012	Descriptive study	Descriptive statistics and Pearson Correlations and Regression Analysis and consumption indicators	Construction Year	-0.21	>0,10	General	Main Study	Primary data	110	kWh/m^2	EUI	R.S.A., T.M., and R.M.U.	27th October to 11th November. 2023	Used in meta-analysis
14	Mingfang Tang, Xiao Fu, Huiming Cao, Yuan Shen, Hongbing Deng, Gang Wu	2016	Sustainability	Lijiang, China	26.87	100.24	24	2012	Descriptive study	Descriptive statistics and Pearson Correlations and Regression Analysis and consumption indicators	years after retrofit	0.27	>0,10	General	Main Study	Primary data	110	kWh/m^2	EUI	R.S.A., T.M., and R.M.U.	27th October to 11th November. 2023	Used in meta-analysis
14	Mingfang Tang, Xiao Fu, Huiming Cao, Yuan Shen, Hongbing Deng, Gang Wu	2016	Sustainability	Lijiang, China	26.87	100.24	24	2012	Descriptive study	Descriptive statistics and Pearson Correlations and Regression Analysis and consumption indicators	Revenue	0.14	>0,10	General	Main Study	Primary data	110	kWh/m^2	EUI	R.S.A., T.M., and R.M.U.	27th October to 11th November. 2023	Not used in meta-analysis due number of effect sizes less than 3
14	Mingfang Tang, Xiao Fu, Huiming Cao, Yuan Shen, Hongbing Deng, Gang Wu	2016	Sustainability	Lijiang, China	26.87	100.24	24	2012	Descriptive study	Descriptive statistics and Pearson Correlations and Regression Analysis and consumption indicators	Room revenue	0.57	<0,01	General	Main Study	Primary data	110	kWh/m^2	EUI	R.S.A., T.M., and R.M.U.	27th October to 11th November. 2023	Used in meta-analysis
14	Mingfang Tang, Xiao Fu, Huiming Cao, Yuan Shen, Hongbing Deng, Gang Wu	2016	Sustainability	Lijiang, China	26.87	100.24	24	2012	Descriptive study	Descriptive statistics and Pearson Correlations and Regression Analysis and consumption indicators	food and beverage revenue	0.00	>0,10	General	Main Study	Primary data	110	kWh/m^2	EUI	R.S.A., T.M., and R.M.U.	27th October to 11th November. 2023	Not used in meta-analysis due number of effect sizes less than 3
14	Mingfang Tang, Xiao Fu, Huiming Cao, Yuan Shen, Hongbing Deng, Gang Wu	2016	Sustainability	Lijiang, China	26.87	100.24	24	2012	Descriptive study	Descriptive statistics and Pearson Correlations and Regression Analysis and consumption indicators	area of guestroom	0.36	<0,05	General	Main Study	Primary data	110	kWh/m^2	EUI	R.S.A., T.M., and R.M.U.	27th October to 11th November. 2023	Used in meta-analysis
14	Mingfang Tang, Xiao Fu, Huiming Cao, Yuan Shen, Hongbing Deng, Gang Wu	2016	Sustainability	Lijiang, China	26.87	100.24	24	2012	Descriptive study	Descriptive statistics and Pearson Correlations and Regression Analysis and consumption indicators	number of employees	0.53	<0,01	General	Main Study	Primary data	110	kWh/m^2	EUI	R.S.A., T.M., and R.M.U.	27th October to 11th November. 2023	Used in meta-analysis
14	Mingfang Tang, Xiao Fu, Huiming Cao, Yuan Shen, Hongbing Deng, Gang Wu	2016	Sustainability	Lijiang, China	26.87	100.24	24	2012	Descriptive study	Descriptive statistics and Pearson Correlations and Regression Analysis and consumption indicators	Occupancy rate	0.42	<0,05	General	Main Study	Primary data	110	kWh/m^2	EUI	R.S.A., T.M., and R.M.U.	27th October to 11th November. 2023	Used in meta-analysis
15	Rajagopalan Priyadarsini, Wu Xuchao, Lee Siew Eang	2009	Energy and Buildings	Singapore	1.37	103.81	29	2004	Descriptive study	Descriptive statistics, regression techniques, consumption indicators and benchmarking	Occupancy rate	0.25	>0,05	General	Second use in the same study	Primary data	427	kWh/m^2	EUI	R.S.A., T.M., and R.M.U.	27th October to 11th November. 2023	Used in meta-analysis
15	Rajagopalan Priyadarsini, Wu Xuchao, Lee Siew Eang	2009	Energy and Buildings	Singapore	1.37	103.81	29	2004	Descriptive study	Descriptive statistics, regression techniques, consumption indicators and benchmarking	floor area	0.95	NI	General	Main Study	Primary data	9000	MWh/year	raw	R.S.A., T.M., and R.M.U.	27th October to 11th November. 2023	Used in meta-analysis
15	Rajagopalan Priyadarsini, Wu Xuchao, Lee Siew Eang	2009	Energy and Buildings	Singapore	1.37	103.81	29	2004	Descriptive study	Descriptive statistics, regression techniques, consumption indicators and benchmarking	Guestrooms	0.85	NI	General	Main Study	Primary data	9000	MWh/year	raw	R.S.A., T.M., and R.M.U.	27th October to 11th November. 2023	Used in meta-analysis
15	Rajagopalan Priyadarsini, Wu Xuchao, Lee Siew Eang	2009	Energy and Buildings	Singapore	1.37	103.81	29	2004	Descriptive study	Descriptive statistics, regression techniques, consumption indicators and benchmarking	guest-nights	0.84	NI	General	Main Study	Primary data	9000	MWh/year	raw	R.S.A., T.M., and R.M.U.	27th October to 11th November. 2023	Used in meta-analysis
15	Rajagopalan Priyadarsini, Wu Xuchao, Lee Siew Eang	2009	Energy and Buildings	Singapore	1.37	103.81	29	2004	Descriptive study	Descriptive statistics, regression techniques, consumption indicators and benchmarking	number of employees	0.92	NI	General	Main Study	Primary data	9000	MWh/year	raw	R.S.A., T.M., and R.M.U.	27th October to 11th November. 2023	Used in meta-analysis
15	Rajagopalan Priyadarsini, Wu Xuchao, Lee Siew Eang	2009	Energy and Buildings	Singapore	1.37	103.81	29	2004	Descriptive study	Descriptive statistics, regression techniques, consumption indicators and benchmarking	floor area	0.17	>0,05	General	Second use in the same study	Primary data	427	kWh/m^2	EUI	R.S.A., T.M., and R.M.U.	27th October to 11th November. 2023	Used in meta-analysis
15	Rajagopalan Priyadarsini, Wu Xuchao, Lee Siew Eang	2009	Energy and Buildings	Singapore	1.37	103.81	29	2004	Descriptive study	Descriptive statistics, regression techniques, consumption indicators and benchmarking	number of floors	0.44	<0,05	General	Second use in the same study	Primary data	427	kWh/m^2	EUI	R.S.A., T.M., and R.M.U.	27th October to 11th November. 2023	Used in meta-analysis
15	Rajagopalan Priyadarsini, Wu Xuchao, Lee Siew Eang	2009	Energy and Buildings	Singapore	1.37	103.81	29	2004	Descriptive study	Descriptive statistics, regression techniques, consumption indicators and benchmarking	Guestrooms	0.30	>0,05	General	Second use in the same study	Primary data	427	kWh/m^2	EUI	R.S.A., T.M., and R.M.U.	27th October to 11th November. 2023	Used in meta-analysis
15	Rajagopalan Priyadarsini, Wu Xuchao, Lee Siew Eang	2009	Energy and Buildings	Singapore	1.37	103.81	29	2004	Descriptive study	Descriptive statistics, regression techniques, consumption indicators and benchmarking	area of guestroom	0.43	<0,05	General	Second use in the same study	Primary data	427	kWh/m^2	EUI	R.S.A., T.M., and R.M.U.	27th October to 11th November. 2023	Used in meta-analysis
15	Rajagopalan Priyadarsini, Wu Xuchao, Lee Siew Eang	2009	Energy and Buildings	Singapore	1.37	103.81	29	2004	Descriptive study	Descriptive statistics, regression techniques, consumption indicators and benchmarking	Construction Year	-0.21	>0,05	General	Second use in the same study	Primary data	427	kWh/m^2	EUI	R.S.A., T.M., and R.M.U.	27th October to 11th November. 2023	Used in meta-analysis
15	Rajagopalan Priyadarsini, Wu Xuchao, Lee Siew Eang	2009	Energy and Buildings	Singapore	1.37	103.81	29	2004	Descriptive study	Descriptive statistics, regression techniques, consumption indicators and benchmarking	years after retrofit	0.53	<0,01	General	Second use in the same study	Primary data	427	kWh/m^2	EUI	R.S.A., T.M., and R.M.U.	27th October to 11th November. 2023	Used in meta-analysis
15	Rajagopalan Priyadarsini, Wu Xuchao, Lee Siew Eang	2009	Energy and Buildings	Singapore	1.37	103.81	29	2004	Descriptive study	Descriptive statistics, regression techniques, consumption indicators and benchmarking	workers per shift	0.47	<0,01	General	Second use in the same study	Primary data	427	kWh/m^2	EUI	R.S.A., T.M., and R.M.U.	27th October to 11th November. 2023	Not used in meta-analysis due number of effect sizes less than 3
15	Rajagopalan Priyadarsini, Wu Xuchao, Lee Siew Eang	2009	Energy and Buildings	Singapore	1.37	103.81	29	2004	Descriptive study	Descriptive statistics, regression techniques, consumption indicators and benchmarking	worker density	0.67	<0,01	General	Second use in the same study	Primary data	427	kWh/m^2	EUI	R.S.A., T.M., and R.M.U.	27th October to 11th November. 2023	Used in meta-analysis
15	Rajagopalan Priyadarsini, Wu Xuchao, Lee Siew Eang	2009	Energy and Buildings	Singapore	1.37	103.81	29	2004	Descriptive study	Descriptive statistics, regression techniques, consumption indicators and benchmarking	energy audit	0.37	<0,05	General	Second use in the same study	Primary data	427	kWh/m^2	EUI	R.S.A., T.M., and R.M.U.	27th October to 11th November. 2023	Not used in meta-analysis due number of effect sizes less than 3
15	Rajagopalan Priyadarsini, Wu Xuchao, Lee Siew Eang	2009	Energy and Buildings	Singapore	1.37	103.81	29	2004	Descriptive study	Descriptive statistics, regression techniques, consumption indicators and benchmarking	laundry	0.37	>0,05	General	Second use in the same study	Primary data	427	kWh/m^2	EUI	R.S.A., T.M., and R.M.U.	27th October to 11th November. 2023	Not used in meta-analysis due number of effect sizes less than 3
15	Rajagopalan Priyadarsini, Wu Xuchao, Lee Siew Eang	2009	Energy and Buildings	Singapore	1.37	103.81	29	2004	Descriptive study	Descriptive statistics, regression techniques, consumption indicators and benchmarking	5 star	0.35	>0,05	General	Second use in the same study	Primary data	427	kWh/m^2	EUI	R.S.A., T.M., and R.M.U.	27th October to 11th November. 2023	Not used in meta-analysis due number of effect sizes less than 3
15	Rajagopalan Priyadarsini, Wu Xuchao, Lee Siew Eang	2009	Energy and Buildings	Singapore	1.37	103.81	29	2004	Descriptive study	Descriptive statistics, regression techniques, consumption indicators and benchmarking	4 star	0.17	>0,05	General	Second use in the same study	Primary data	427	kWh/m^2	EUI	R.S.A., T.M., and R.M.U.	27th October to 11th November. 2023	Not used in meta-analysis due number of effect sizes less than 3
15	Rajagopalan Priyadarsini, Wu Xuchao, Lee Siew Eang	2009	Energy and Buildings	Singapore	1.37	103.81	29	2004	Descriptive study	Descriptive statistics, regression techniques, consumption indicators and benchmarking	3 star	-0.67	<0,01	General	Second use in the same study	Primary data	427	kWh/m^2	EUI	R.S.A., T.M., and R.M.U.	27th October to 11th November. 2023	Not used in meta-analysis due number of effect sizes less than 3
15	Rajagopalan Priyadarsini, Wu Xuchao, Lee Siew Eang	2009	Energy and Buildings	Singapore	1.37	103.81	13	2004	Descriptive study	Descriptive statistics, regression techniques, consumption indicators and benchmarking	ambient temperature	0.71	NI	General	Second use in the same study	Primary data	750	MWh/month	raw	R.S.A., T.M., and R.M.U.	27th October to 11th November. 2023	Used in meta-analysis

15	Rajagopalan Priyadarsini, Wu Xuchao, Lee Siew Eang	2009	Energy and Buildings	Singapore	1.37	103.81	29	2004	Descriptive study	Descriptive statistics, regression techniques, consumption indicators and benchmarking	occupied rooms	0.76	NI	General	Main Study	Primary data	9000	MWh/year	raw	R.S.A., T.M., and R.M.U.	27th October to 11th November. 2023	Used in meta-analysis
16	Paulina Bohdanowicz, Ivo Martinac	2007	Energy and Buildings	Europe	54.3	15.04	73	2004	Descriptive study	Descriptive statistics, consumption indicators, multiple regression, benchmarking	floor area	0.84	NI	Upscale	Main Study	Secondary data	11000	MWh/year	raw	R.S.A., T.M., and R.M.U.	27th October to 11th November. 2023	Used in meta-analysis
16	Paulina Bohdanowicz, Ivo Martinac	2007	Energy and Buildings	Europe	54.3	15.04	111	2004	Descriptive study	Descriptive statistics, consumption indicators, multiple regression, benchmarking	floor area	0.87	NI	General	Main Study	Secondary data	6000	MWh/year	raw	R.S.A., T.M., and R.M.U.	27th October to 11th November. 2023	Used in meta-analysis
16	Paulina Bohdanowicz, Ivo Martinac	2007	Energy and Buildings	Europe	54.3	15.04	73	2004	Descriptive study	Descriptive statistics, consumption indicators, multiple regression, benchmarking	guest-nights	0.62	NI	Upscale	Main Study	Secondary data	11000	MWh/year	raw	R.S.A., T.M., and R.M.U.	27th October to 11th November. 2023	Used in meta-analysis
16	Paulina Bohdanowicz, Ivo Martinac	2007	Energy and Buildings	Europe	54.3	15.04	111	2004	Descriptive study	Descriptive statistics, consumption indicators, multiple regression, benchmarking	guest-nights	0.82	NI	General	Main Study	Secondary data	6000	MWh/year	raw	R.S.A., T.M., and R.M.U.	27th October to 11th November. 2023	Used in meta-analysis
16	Paulina Bohdanowicz, Ivo Martinac	2007	Energy and Buildings	Europe	54.3	15.04	73	2004	Descriptive study	Descriptive statistics, consumption indicators, multiple regression, benchmarking	food covers	0.71	NI	Upscale	Main Study	Secondary data	11000	MWh/year	raw	R.S.A., T.M., and R.M.U.	27th October to 11th November. 2023	Used in meta-analysis
16	Paulina Bohdanowicz, Ivo Martinac	2007	Energy and Buildings	Europe	54.3	15.04	111	2004	Descriptive study	Descriptive statistics, consumption indicators, multiple regression, benchmarking	food covers	0.81	NI	General	Main Study	Secondary data	6000	MWh/year	raw	R.S.A., T.M., and R.M.U.	27th October to 11th November. 2023	Used in meta-analysis
16	Paulina Bohdanowicz, Ivo Martinac	2007	Energy and Buildings	Europe	54.3	15.04	73	2004	Descriptive study	Descriptive statistics, consumption indicators, multiple regression, benchmarking	Construction Year	0.14	NI	Upscale	Main Study	Secondary data	364.3	kWh/m²	EUI	R.S.A., T.M., and R.M.U.	27th October to 11th November. 2023	Used in meta-analysis
16	Paulina Bohdanowicz, Ivo Martinac	2007	Energy and Buildings	Europe	54.3	15.04	111	2004	Descriptive study	Descriptive statistics, consumption indicators, multiple regression, benchmarking	Construction Year	0.07	NI	General	Main Study	Secondary data	285	kWh/m²	EUI	R.S.A., T.M., and R.M.U.	27th October to 11th November. 2023	Used in meta-analysis
16	Paulina Bohdanowicz, Ivo Martinac	2007	Energy and Buildings	Europe	54.3	15.04	73	2004	Descriptive study	Descriptive statistics, consumption indicators, multiple regression, benchmarking	floor area	0.30	NI	Upscale	Second use in the same study	Secondary data	364.3	kWh/m²	EUI	R.S.A., T.M., and R.M.U.	27th October to 11th November. 2023	Used in meta-analysis
16	Paulina Bohdanowicz, Ivo Martinac	2007	Energy and Buildings	Europe	54.3	15.04	111	2004	Descriptive study	Descriptive statistics, consumption indicators, multiple regression, benchmarking	floor area	0.17	NI	General	Second use in the same study	Secondary data	285	kWh/m²	EUI	R.S.A., T.M., and R.M.U.	27th October to 11th November. 2023	Used in meta-analysis
17	Jen Chun Wang, Kuo-Tsang Huang	2013	Energy	Taiwan	23.95	121	34	2010	Descriptive study	Descriptive statistics, correlation analysis, consumption indicators, multiple regression	worker density	0.42	<0,05	4.5	Main Study	Primary data	25200000	MJ/Year	raw	R.S.A., T.M., and R.M.U.	27th October to 11th November. 2023	Used in meta-analysis
17	Jen Chun Wang, Kuo-Tsang Huang	2013	Energy	Taiwan	23.95	121	34	2010	Descriptive study	Descriptive statistics, correlation analysis, consumption indicators, multiple regression	cooling temperature	-0.77	<0,01	4.5	Main Study	Primary data	25200000	MJ/Year	raw	R.S.A., T.M., and R.M.U.	27th October to 11th November. 2023	Not used in meta-analysis due number of effect sizes less than 3
17	Jen Chun Wang, Kuo-Tsang Huang	2013	Energy	Taiwan	23.95	121	34	2010	Descriptive study	Descriptive statistics, correlation analysis, consumption indicators, multiple regression	Addicional services area	0.92	NI	4.5	Main Study	Primary data	207	MJ/guestnight	EUI	R.S.A., T.M., and R.M.U.	27th October to 11th November. 2023	Not used in meta-analysis due number of effect sizes less than 3
17	Jen Chun Wang, Kuo-Tsang Huang	2013	Energy	Taiwan	23.95	121	73	2010	Descriptive study	Descriptive statistics, correlation analysis, consumption indicators, multiple regression	number of guests	0.90	<0,05	4.5	Main Study	Primary data	25200000	MJ/Year	raw	R.S.A., T.M., and R.M.U.	27th October to 11th November. 2023	Used in meta-analysis
17	Jen Chun Wang, Kuo-Tsang Huang	2013	Energy	Taiwan	23.95	121	73	2010	Descriptive study	Descriptive statistics, correlation analysis, consumption indicators, multiple regression	Group of guests	0.84	<0,05	4.5	Main Study	Primary data	25200000	MJ/Year	raw	R.S.A., T.M., and R.M.U.	27th October to 11th November. 2023	Not used in meta-analysis due number of effect sizes less than 3
18	Maja Štimac, Mario Matkovic, Daria Karasalihovic Sedlar	2023	Sustainability	Croatia - Adriatic Coast	45.31	14.86	51	2016-2021	Descriptive study using only the gas consumption	Descriptive statistics, correlation analysis, regression analysis	floor area	0.85	NI	General	Main Study	Primary data	800000	kWh/year	raw	R.S.A., T.M., and R.M.U.	27th October to 11th November. 2023	Used in meta-analysis
18	Maja Štimac, Mario Matkovic, Daria Karasalihovic Sedlar	2023	Sustainability	Croatia - Adriatic Coast	45.31	14.86	51	2016-2021	Descriptive study using only the gas consumption	Descriptive statistics, correlation analysis, regression analysis	Guestrooms	0.67	NI	General	Main Study	Primary data	800000	kWh/year	raw	R.S.A., T.M., and R.M.U.	27th October to 11th November. 2023	Used in meta-analysis
18	Maja Štimac, Mario Matkovic, Daria Karasalihovic Sedlar	2023	Sustainability	Croatia - Adriatic Coast	45.31	14.86	51	2016-2021	Descriptive study using only the gas consumption	Descriptive statistics, correlation analysis, regression analysis	floor area	0.58	NI	General	Second use in the same study	Primary data	40	kWh/m²	EUI	R.S.A., T.M., and R.M.U.	27th October to 11th November. 2023	Used in meta-analysis
19	Silu Bhochhibhoya	2016	Università degli Studi di Padova	Solukhumbu, Nepal	27.74	86.73	45	2014	Life Cycle Assessment (LCA) and Life Cycle Costing (LCC)	Descriptive statistics, ANOVA, correlation analysis	Carbon emissions construction	-0.03	>0,05	General	Main Study	Primary data	10.74	kWh/Person.Night	EUI	R.S.A., T.M., and R.M.U.	27th October to 11th November. 2023	Not used in meta-analysis due number of effect sizes less than 3
19	Silu Bhochhibhoya	2016	Università degli Studi di Padova	Solukhumbu, Nepal	27.74	86.73	45	2014	Life Cycle Assessment (LCA) and Life Cycle Costing (LCC)	Descriptive statistics, ANOVA, correlation analysis	Carbon emission operation	0.89	<0,05	General	Main Study	Primary data	10.74	kWh/Person.Night	EUI	R.S.A., T.M., and R.M.U.	27th October to 11th November. 2023	Not used in meta-analysis due number of effect sizes less than 3
19	Silu Bhochhibhoya	2016	Università degli Studi di Padova	Solukhumbu, Nepal	27.74	86.73	45	2014	Life Cycle Assessment (LCA) and Life Cycle Costing (LCC)	Descriptive statistics, ANOVA, correlation analysis	Carbon emissions replacement	0.39	<0,05	General	Main Study	Primary data	10.74	kWh/Person.Night	EUI	R.S.A., T.M., and R.M.U.	27th October to 11th November. 2023	Not used in meta-analysis due number of effect sizes less than 3
19	Silu Bhochhibhoya	2016	Università degli Studi di Padova	Solukhumbu, Nepal	27.74	86.73	45	2014	Life Cycle Assessment (LCA) and Life Cycle Costing (LCC)	Descriptive statistics, ANOVA, correlation analysis	carbon emissions	0.89	<0,05	General	Main Study	Primary data	10.74	kWh/Person.Night	EUI	R.S.A., T.M., and R.M.U.	27th October to 11th November. 2023	Used in meta-analysis
19	Silu Bhochhibhoya	2016	Università degli Studi di Padova	Solukhumbu, Nepal	27.74	86.73	45	2014	Life Cycle Assessment (LCA) and Life Cycle Costing (LCC)	Descriptive statistics, ANOVA, correlation analysis	Construction cost	0.31	<0,05	General	Main Study	Primary data	10.74	kWh/Person.Night	EUI	R.S.A., T.M., and R.M.U.	27th October to 11th November. 2023	Not used in meta-analysis due number of effect sizes less than 3
19	Silu Bhochhibhoya	2016	Università degli Studi di Padova	Solukhumbu, Nepal	27.74	86.73	45	2014	Life Cycle Assessment (LCA) and Life Cycle Costing (LCC)	Descriptive statistics, ANOVA, correlation analysis	Operation cost	0.41	<0,05	General	Main Study	Primary data	10.74	kWh/Person.Night	EUI	R.S.A., T.M., and R.M.U.	27th October to 11th November. 2023	Not used in meta-analysis due number of effect sizes less than 3
19	Silu Bhochhibhoya	2016	Università degli Studi di Padova	Solukhumbu, Nepal	27.74	86.73	45	2014	Life Cycle Assessment (LCA) and Life Cycle Costing (LCC)	Descriptive statistics, ANOVA, correlation analysis	Maintenance cost replacement	0.27	>0,05	General	Main Study	Primary data	10.74	kWh/Person.Night	EUI	R.S.A., T.M., and R.M.U.	27th October to 11th November. 2023	Not used in meta-analysis due number of effect sizes less than 3
19	Silu Bhochhibhoya	2016	Università degli Studi di Padova	Solukhumbu, Nepal	27.74	86.73	45	2014	Life Cycle Assessment (LCA) and Life Cycle Costing (LCC)	Descriptive statistics, ANOVA, correlation analysis	custo total	0.43	<0,05	General	Main Study	Primary data	10.74	kWh/Person.Night	EUI	R.S.A., T.M., and R.M.U.	27th October to 11th November. 2023	Not used in meta-analysis due number of effect sizes less than 3
19	Silu Bhochhibhoya	2016	Università degli Studi di Padova	Solukhumbu, Nepal	27.74	86.73	45	2014	Life Cycle Assessment (LCA) and Life Cycle Costing (LCC)	Descriptive statistics, ANOVA, correlation analysis	Loss rate	-0.02	>0,05	General	Main Study	Primary data	10.74	kWh/Person.Night	EUI	R.S.A., T.M., and R.M.U.	27th October to 11th November. 2023	Not used in meta-analysis due number of effect sizes less than 3
19	Silu Bhochhibhoya	2016	Università degli Studi di Padova	Solukhumbu, Nepal	27.74	86.73	45	2014	Life Cycle Assessment (LCA) and Life Cycle Costing (LCC)	Descriptive statistics, ANOVA, correlation analysis	number of guests	-0.48	<0,05	General	Main Study	Primary data	10.74	kWh/Person.Night	EUI	R.S.A., T.M., and R.M.U.	27th October to 11th November. 2023	Used in meta-analysis
19	Silu Bhochhibhoya	2016	Università degli Studi di Padova	Solukhumbu, Nepal	27.74	86.73	45	2014	Life Cycle Assessment (LCA) and Life Cycle Costing (LCC)	Descriptive statistics, ANOVA, correlation analysis	Building area	-0.33	<0,05	General	Main Study	Primary data	10.74	kWh/Person.Night	EUI	R.S.A., T.M., and R.M.U.	27th October to 11th November. 2023	Not used in meta-analysis due number of effect sizes less than 3
20	Houcem Eddine Mechri, Samir Amara	2021	Energy and Buildings	Tunisia	35.94	9.83	130	NI	Descriptive study	Descriptive statistics, correlation analysis, energy use indicators	Occupancy rate	0.00	NI	General	Main Study	Primary data	897	toe/year	raw	R.S.A., T.M., and R.M.U.	27th October to 11th November. 2023	Used in meta-analysis
20	Houcem Eddine Mechri, Samir Amara	2021	Energy and Buildings	Tunisia	35.94	9.83	55	NI	Descriptive study (coleta de dados sobre floor area teve problemas)	Descriptive statistics, correlation analysis, energy use indicators	floor area	NA	NI	General	Main Study	Primary data	897	toe/year	raw	R.S.A., T.M., and R.M.U.	27th October to 11th November. 2023	Not used in meta-analysis due problems in data collection (variable floor area together/mixed with building area)
20	Houcem Eddine Mechri, Samir Amara	2021	Energy and Buildings	Tunisia	35.94	9.83	55	NI	Descriptive study	Descriptive statistics, correlation analysis, energy use indicators	area of guestroom	0.56	NI	General	Main Study	Primary data	897	toe/year	raw	R.S.A., T.M., and R.M.U.	27th October to 11th November. 2023	Used in meta-analysis
20	Houcem Eddine Mechri, Samir Amara	2021	Energy and Buildings	Tunisia	35.94	9.83	111	NI	Descriptive study	Descriptive statistics, correlation analysis, energy use indicators	Guestrooms	0.45	NI	General	Main Study	Primary data	897	toe/year	raw	R.S.A., T.M., and R.M.U.	27th October to 11th November. 2023	Used in meta-analysis
20	Houcem Eddine Mechri, Samir Amara	2021	Energy and Buildings	Tunisia	35.94	9.83	103	NI	Descriptive study	Descriptive statistics, correlation analysis, energy use indicators	Number of beds	0.41	NI	General	Main Study	Primary data	897	toe/year	raw	R.S.A., T.M., and R.M.U.	27th October to 11th November. 2023	Not used in meta-analysis due number of effect sizes less than 3
20	Houcem Eddine Mechri, Samir Amara	2021	Energy and Buildings	Tunisia	35.94	9.83	133	NI	Descriptive study	Descriptive statistics, correlation analysis, energy use indicators	guest-nights	0.35	NI	General	Main Study	Primary data	897	toe/year	raw	R.S.A., T.M., and R.M.U.	27th October to 11th November. 2023	Used in meta-analysis
20	Houcem Eddine Mechri, Samir Amara	2021	Energy and Buildings	Tunisia	35.94	9.83	85	NI	Descriptive study	Descriptive statistics, correlation analysis, energy use indicators	food covers	0.28	NI	General	Main Study	Primary data	897	toe/year	raw	R.S.A., T.M., and R.M.U.	27th October to 11th November. 2023	Used in meta-analysis
21	Jen Chun Wang	2012	Energy and Buildings	Taiwan	23.95	121	200	2010	Descriptive study	Descriptive statistics, correlation analysis, energy use indicators, regression analysis	ambient temperature	0.88	NI	General	Main Study	Primary data	7000	MWh/year	raw	R.S.A., T.M., and R.M.U.	27th October to 11th November. 2023	Used in meta-analysis

21	Jen Chun Wang	2012	Energy and Buildings	Taiwan	23.95	121	200	2010	Descriptive study	Descriptive statistics, correlation analysis, energy use indicators, regression analysis	floor area	0.92	<0,01	General	Main Study	Primary data	7000	MWh/year	raw	R.S.A., T.M., and R.M.U.	27th October to 11th November. 2023	Used in meta-analysis
21	Jen Chun Wang	2012	Energy and Buildings	Taiwan	23.95	121	200	2010	Descriptive study	Descriptive statistics, correlation analysis, energy use indicators, regression analysis	floor area	0.17	<0,05	General	Second use in the same study	Primary data	200	kWh/m²	EUI	R.S.A., T.M., and R.M.U.	27th October to 11th November. 2023	Used in meta-analysis
21	Jen Chun Wang	2012	Energy and Buildings	Taiwan	23.95	121	200	2010	Descriptive study	Descriptive statistics, correlation analysis, energy use indicators, regression analysis	building stories	0.68	<0,01	General	Main Study	Primary data	7000	MWh/year	raw	R.S.A., T.M., and R.M.U.	27th October to 11th November. 2023	Not used in meta-analysis due number of effect sizes less than 3
21	Jen Chun Wang	2012	Energy and Buildings	Taiwan	23.95	121	200	2010	Descriptive study	Descriptive statistics, correlation analysis, energy use indicators, regression analysis	Construction Year	0.24	<0,01	General	Main Study	Primary data	7000	MWh/year	not used in meta-analysis (RAW)	R.S.A., T.M., and R.M.U.	27th October to 11th November. 2023	Not used in meta-analysis due duplicated data
21	Jen Chun Wang	2012	Energy and Buildings	Taiwan	23.95	121	200	2010	Descriptive study	Descriptive statistics, correlation analysis, energy use indicators, regression analysis	Construction Year	-0.15	<0,05	General	Second use in the same study	Primary data	200	kWh/m²	EUI	R.S.A., T.M., and R.M.U.	27th October to 11th November. 2023	Used in meta-analysis
21	Jen Chun Wang	2012	Energy and Buildings	Taiwan	23.95	121	200	2010	Descriptive study	Descriptive statistics, correlation analysis, energy use indicators, regression analysis	Category (star level)	0.74	<0,01	General	Main Study	Primary data	7000	MWh/year	raw	R.S.A., T.M., and R.M.U.	27th October to 11th November. 2023	Used in meta-analysis
21	Jen Chun Wang	2012	Energy and Buildings	Taiwan	23.95	121	200	2010	Descriptive study	Descriptive statistics, correlation analysis, energy use indicators, regression analysis	location district	0.27	<0,01	General	Main Study	Primary data	7000	MWh/year	raw	R.S.A., T.M., and R.M.U.	27th October to 11th November. 2023	Not used in meta-analysis due number of effect sizes less than 3
21	Jen Chun Wang	2012	Energy and Buildings	Taiwan	23.95	121	200	2010	Descriptive study	Descriptive statistics, correlation analysis, energy use indicators, regression analysis	Guestrooms	0.93	<0,01	General	Main Study	Primary data	7000	MWh/year	raw	R.S.A., T.M., and R.M.U.	27th October to 11th November. 2023	Used in meta-analysis
21	Jen Chun Wang	2012	Energy and Buildings	Taiwan	23.95	121	200	2010	Descriptive study	Descriptive statistics, correlation analysis, energy use indicators, regression analysis	Guestrooms	0.30	<0,01	General	Second use in the same study	Primary data	200	kWh/m²	EUI	R.S.A., T.M., and R.M.U.	27th October to 11th November. 2023	Used in meta-analysis
21	Jen Chun Wang	2012	Energy and Buildings	Taiwan	23.95	121	200	2010	Descriptive study	Descriptive statistics, correlation analysis, energy use indicators, regression analysis	GFA/guestrooms	0.51	<0,01	General	Main Study	Primary data	7000	MWh/year	raw	R.S.A., T.M., and R.M.U.	27th October to 11th November. 2023	Not used in meta-analysis due number of effect sizes less than 3
21	Jen Chun Wang	2012	Energy and Buildings	Taiwan	23.95	121	200	2010	Descriptive study	Descriptive statistics, correlation analysis, energy use indicators, regression analysis	(%) of GFA with dining facilities	0.14	<0,05	General	Main Study	Primary data	7000	MWh/year	raw	R.S.A., T.M., and R.M.U.	27th October to 11th November. 2023	Not used in meta-analysis due number of effect sizes less than 3
21	Jen Chun Wang	2012	Energy and Buildings	Taiwan	23.95	121	200	2010	Descriptive study	Descriptive statistics, correlation analysis, energy use indicators, regression analysis	(%) of GFA with retail shops	0.15	<0,05	General	Main Study	Primary data	7000	MWh/year	raw	R.S.A., T.M., and R.M.U.	27th October to 11th November. 2023	Not used in meta-analysis due number of effect sizes less than 3
21	Jen Chun Wang	2012	Energy and Buildings	Taiwan	23.95	121	200	2010	Descriptive study	Descriptive statistics, correlation analysis, energy use indicators, regression analysis	Occupancy rate	0.64	<0,01	General	Main Study	Primary data	7000	MWh/year	raw	R.S.A., T.M., and R.M.U.	27th October to 11th November. 2023	Used in meta-analysis
21	Jen Chun Wang	2012	Energy and Buildings	Taiwan	23.95	121	200	2010	Descriptive study	Descriptive statistics, correlation analysis, energy use indicators, regression analysis	Occupancy rate	0.71	<0,01	General	Second use in the same study	Primary data	200	kWh/m²	EUI	R.S.A., T.M., and R.M.U.	27th October to 11th November. 2023	Used in meta-analysis
21	Jen Chun Wang	2012	Energy and Buildings	Taiwan	23.95	121	200	2010	Descriptive study	Descriptive statistics, correlation analysis, energy use indicators, regression analysis	average room rate	0.47	<0,01	General	Main Study	Primary data	7000	MWh/year	raw	R.S.A., T.M., and R.M.U.	27th October to 11th November. 2023	Not used in meta-analysis due number of effect sizes less than 3
21	Jen Chun Wang	2012	Energy and Buildings	Taiwan	23.95	121	200	2010	Descriptive study	Descriptive statistics, correlation analysis, energy use indicators, regression analysis	Room revenue	0.92	<0,01	General	Main Study	Primary data	7000	MWh/year	raw	R.S.A., T.M., and R.M.U.	27th October to 11th November. 2023	Used in meta-analysis
21	Jen Chun Wang	2012	Energy and Buildings	Taiwan	23.95	121	200	2010	Descriptive study	Descriptive statistics, correlation analysis, energy use indicators, regression analysis	food and beverage revenue	0.82	<0,01	General	Main Study	Primary data	7000	MWh/year	raw	R.S.A., T.M., and R.M.U.	27th October to 11th November. 2023	Not used in meta-analysis due number of effect sizes less than 3
21	Jen Chun Wang	2012	Energy and Buildings	Taiwan	23.95	121	200	2010	Descriptive study	Descriptive statistics, correlation analysis, energy use indicators, regression analysis	revenue	0.85	<0,01	General	Main Study	Primary data	7000	MWh/year	raw	R.S.A., T.M., and R.M.U.	27th October to 11th November. 2023	Not used in meta-analysis due number of effect sizes less than 3
21	Jen Chun Wang	2012	Energy and Buildings	Taiwan	23.95	121	200	2010	Descriptive study	Descriptive statistics, correlation analysis, energy use indicators, regression analysis	number of employees	0.90	<0,01	General	Main Study	Primary data	7000	MWh/year	raw	R.S.A., T.M., and R.M.U.	27th October to 11th November. 2023	Used in meta-analysis
21	Jen Chun Wang	2012	Energy and Buildings	Taiwan	23.95	121	200	2010	Descriptive study	Descriptive statistics, correlation analysis, energy use indicators, regression analysis	worker density	0.04	>0,05	General	Main Study	Primary data	7000	MWh/year	raw	R.S.A., T.M., and R.M.U.	27th October to 11th November. 2023	Used in meta-analysis
21	Jen Chun Wang	2012	Energy and Buildings	Taiwan	23.95	121	200	2010	Descriptive study	Descriptive statistics, correlation analysis, energy use indicators, regression analysis	number of guests	0.93	<0,01	General	Main Study	Primary data	7000	MWh/year	raw	R.S.A., T.M., and R.M.U.	27th October to 11th November. 2023	Used in meta-analysis
21	Jen Chun Wang	2012	Energy and Buildings	Taiwan	23.95	121	200	2010	Descriptive study	Descriptive statistics, correlation analysis, energy use indicators, regression analysis	guest of foreing independent tourist	0.86	<0,01	General	Main Study	Primary data	7000	MWh/year	raw	R.S.A., T.M., and R.M.U.	27th October to 11th November. 2023	Not used in meta-analysis due number of effect sizes less than 3
21	Jen Chun Wang	2012	Energy and Buildings	Taiwan	23.95	121	200	2010	Descriptive study	Descriptive statistics, correlation analysis, energy use indicators, regression analysis	guest of group	0.78	<0,01	General	Main Study	Primary data	7000	MWh/year	raw	R.S.A., T.M., and R.M.U.	27th October to 11th November. 2023	Not used in meta-analysis due number of effect sizes less than 3
21	Jen Chun Wang	2012	Energy and Buildings	Taiwan	23.95	121	200	2010	Descriptive study	Descriptive statistics, correlation analysis, energy use indicators, regression analysis	guests from taiwan	0.49	<0,01	General	Main Study	Primary data	7000	MWh/year	raw	R.S.A., T.M., and R.M.U.	27th October to 11th November. 2023	Not used in meta-analysis due number of effect sizes less than 3
21	Jen Chun Wang	2012	Energy and Buildings	Taiwan	23.95	121	200	2010	Descriptive study	Descriptive statistics, correlation analysis, energy use indicators, regression analysis	guests from china	0.55	<0,01	General	Main Study	Primary data	7000	MWh/year	raw	R.S.A., T.M., and R.M.U.	27th October to 11th November. 2023	Not used in meta-analysis due number of effect sizes less than 3
21	Jen Chun Wang	2012	Energy and Buildings	Taiwan	23.95	121	200	2010	Descriptive study	Descriptive statistics, correlation analysis, energy use indicators, regression analysis	guests from north america	0.85	<0,01	General	Main Study	Primary data	7000	MWh/year	raw	R.S.A., T.M., and R.M.U.	27th October to 11th November. 2023	Not used in meta-analysis due number of effect sizes less than 3
21	Jen Chun Wang	2012	Energy and Buildings	Taiwan	23.95	121	200	2010	Descriptive study	Descriptive statistics, correlation analysis, energy use indicators, regression analysis	guests from japan	0.72	<0,01	General	Main Study	Primary data	7000	MWh/year	raw	R.S.A., T.M., and R.M.U.	27th October to 11th November. 2023	Not used in meta-analysis due number of effect sizes less than 3
21	Jen Chun Wang	2012	Energy and Buildings	Taiwan	23.95	121	200	2010	Descriptive study	Descriptive statistics, correlation analysis, energy use indicators, regression analysis	guest from europe	0.83	<0,01	General	Main Study	Primary data	7000	MWh/year	raw	R.S.A., T.M., and R.M.U.	27th October to 11th November. 2023	Not used in meta-analysis due number of effect sizes less than 3
22	George Papageorgiou, Andreas Efstathiades, Nicolas Nicolaou, Athanasios Maimaris	2018	IEEE International Energy Conference (ENERGYCON)	Cyprus	35.19	33.37	12	2017	Descriptive study	Correlation analysis	ambient temperature	0.99	<0,01	General	Main Study	Primary data	200000	kWh/year	raw	R.S.A., T.M., and R.M.U.	27th October to 11th November. 2023	Used in meta-analysis
23	Wilco W. Chan, Joseph C. Lam	2002	Journal of Sustainable Tourism	Hong Kong	22.4	114.15	11	1994-1996	Descriptive study (Considering only the gas consumption for energy use)	Linear Regression	occupied rooms	0.52	NI	General	Main Study	Primary data	8	TJ	raw	R.S.A., T.M., and R.M.U.	27th October to 11th November. 2023	Used in meta-analysis
23	Wilco W. Chan, Joseph C. Lam	2002	Journal of Sustainable Tourism	Hong Kong	22.4	114.15	11	1994-1996	Descriptive study (Considering only the gas consumption for energy use)	Linear Regression	floor area	0.92	NI	General	Main Study	Primary data	8	TJ	raw	R.S.A., T.M., and R.M.U.	27th October to 11th November. 2023	Used in meta-analysis
23	Wilco W. Chan, Joseph C. Lam	2002	Journal of Sustainable Tourism	Hong Kong	22.4	114.15	11	1994-1996	Descriptive study (Considering only the gas consumption for energy use)	Linear Regression	floor area	-0.64	NI	General	Second use in the same study	Primary data	259.27	MJ/m²	EUI	R.S.A., T.M., and R.M.U.	27th October to 11th November. 2023	Used in meta-analysis
23	Wilco W. Chan, Joseph C. Lam	2002	Journal of Sustainable Tourism	Hong Kong	22.4	114.15	11	1994-1996	Descriptive study (Considering only the gas consumption for energy use)	Linear Regression	Heating degree days	0.03	NI	General	Main Study	Primary data	8	TJ	raw	R.S.A., T.M., and R.M.U.	27th October to 11th November. 2023	Not used in meta-analysis due number of effect sizes less than 3

24	Juan José Cabello Eras, Vladimir Sousa Santos, Alexis Sagastume Gutiérrez, Mario Álvarez Guerra Plasencia, Dries Haeseldonckx, Carlo Vandecasteele	2016	Journal of Cleaner Production	Province of Cienfuegos, Cuba	22.16	-80.43	2	2011-2012	Descriptive study	Energy use indicators, descriptive statistics, Statistical Process Control, correlation analysis	Room Degree Day - RDD (CDD (tempexter.-tempref) vezes a occupancy)	0.88	NI	General	Main Study	Primary data	80	MWh/month	raw	R.S.A., T.M., and R.M.U.	27th October to 11th November. 2023	Not used in meta-analysis due number of effect sizes less than 3
25	Xiangfei Kong, Shilei Lu, Ping Gao, Neng Zhu, Wei Wu, Xuming Gao	2012	Energy and Buildings	Hainan Province, China	19.22	109.8	26	2009	Descriptive study	Descriptive statistics, Pearson correlations, Linear regression, benchmarking analyses and consumption indicators	Occupancy rate	0.03	>0,05	4.5	Second use of data from study 14	Secondary data	6000000	kWh/year	raw	R.S.A., T.M., and R.M.U.	27th October to 11th November. 2023	Used in meta-analysis
25	Xiangfei Kong, Shilei Lu, Ping Gao, Neng Zhu, Wei Wu, Xuming Gao	2012	Energy and Buildings	Hainan Province, China	19.22	109.8	26	2009	Descriptive study	Descriptive statistics, Pearson correlations, Linear regression, benchmarking analyses and consumption indicators	floor area	0.76	<0,01	4.5	Second use of data from study 14	Secondary data	6000000	kWh/year	raw	R.S.A., T.M., and R.M.U.	27th October to 11th November. 2023	Used in meta-analysis
25	Xiangfei Kong, Shilei Lu, Ping Gao, Neng Zhu, Wei Wu, Xuming Gao	2012	Energy and Buildings	Hainan Province, China	19.22	109.8	26	2009	Descriptive study	Descriptive statistics, Pearson correlations, Linear regression, benchmarking analyses and consumption indicators	Category (star level)	0.33	>0,05	4.5	Second use of data from study 14	Secondary data	6000000	kWh/year	raw	R.S.A., T.M., and R.M.U.	27th October to 11th November. 2023	Used in meta-analysis
25	Xiangfei Kong, Shilei Lu, Ping Gao, Neng Zhu, Wei Wu, Xuming Gao	2012	Energy and Buildings	Hainan Province, China	19.22	109.8	26	2009	Descriptive study	Descriptive statistics, Pearson correlations, Linear regression, benchmarking analyses and consumption indicators	number of floors	0.06	>0,05	4.5	Second use of data from study 14	Secondary data	6000000	kWh/year	raw	R.S.A., T.M., and R.M.U.	27th October to 11th November. 2023	Used in meta-analysis
25	Xiangfei Kong, Shilei Lu, Ping Gao, Neng Zhu, Wei Wu, Xuming Gao	2012	Energy and Buildings	Hainan Province, China	19.22	109.8	26	2009	Descriptive study	Descriptive statistics, Pearson correlations, Linear regression, benchmarking analyses and consumption indicators	GFA/guestrooms	0.43	<0,05	4.5	Second use of data from study 14	Secondary data	6000000	kWh/year	raw	R.S.A., T.M., and R.M.U.	27th October to 11th November. 2023	Not used in meta-analysis due number of effect sizes less than 3
25	Xiangfei Kong, Shilei Lu, Ping Gao, Neng Zhu, Wei Wu, Xuming Gao	2012	Energy and Buildings	Hainan Province, China	19.22	109.8	26	2009	Descriptive study	Descriptive statistics, Pearson correlations, Linear regression, benchmarking analyses and consumption indicators	GFA/AirCond	0.27	>0,05	4.5	Second use of data from study 14	Secondary data	6000000	kWh/year	raw	R.S.A., T.M., and R.M.U.	27th October to 11th November. 2023	Not used in meta-analysis due number of effect sizes less than 3
25	Xiangfei Kong, Shilei Lu, Ping Gao, Neng Zhu, Wei Wu, Xuming Gao	2012	Energy and Buildings	Hainan Province, China	19.22	109.8	26	2009	Descriptive study	Descriptive statistics, Pearson correlations, Linear regression, benchmarking analyses and consumption indicators	Illumination of guestrooms	-0.20	>0,05	4.5	Second use of data from study 14	Secondary data	6000000	kWh/year	raw	R.S.A., T.M., and R.M.U.	27th October to 11th November. 2023	Not used in meta-analysis due number of effect sizes less than 3
25	Xiangfei Kong, Shilei Lu, Ping Gao, Neng Zhu, Wei Wu, Xuming Gao	2012	Energy and Buildings	Hainan Province, China	19.22	109.8	26	2009	Descriptive study	Descriptive statistics, Pearson correlations, Linear regression, benchmarking analyses and consumption indicators	building age	0.08	>0,05	4.5	Second use of data from study 14	Secondary data	6000000	kWh/year	raw	R.S.A., T.M., and R.M.U.	27th October to 11th November. 2023	Used in meta-analysis
25	Xiangfei Kong, Shilei Lu, Ping Gao, Neng Zhu, Wei Wu, Xuming Gao	2012	Energy and Buildings	Hainan Province, China	19.22	109.8	26	2009	Descriptive study	Descriptive statistics, Pearson correlations, Linear regression, benchmarking analyses and consumption indicators	Central air cond	0.46	<0,05	4.5	Second use of data from study 14	Secondary data	6000000	kWh/year	raw	R.S.A., T.M., and R.M.U.	27th October to 11th November. 2023	Not used in meta-analysis due number of effect sizes less than 3
25	Xiangfei Kong, Shilei Lu, Ping Gao, Neng Zhu, Wei Wu, Xuming Gao	2012	Energy and Buildings	Hainan Province, China	19.22	109.8	26	2009	Descriptive study	Descriptive statistics, Pearson correlations, Linear regression, benchmarking analyses and consumption indicators	Guestrooms	0.57	<0,01	4.5	Second use of data from study 14	Secondary data	6000000	kWh/year	raw	R.S.A., T.M., and R.M.U.	27th October to 11th November. 2023	Used in meta-analysis
25	Xiangfei Kong, Shilei Lu, Ping Gao, Neng Zhu, Wei Wu, Xuming Gao	2012	Energy and Buildings	Hainan Province, China	19.22	109.8	26	2009	Descriptive study	Descriptive statistics, Pearson correlations, Linear regression, benchmarking analyses and consumption indicators	external wall type	0.11	>0,05	4.5	Second use of data from study 14	Secondary data	6000000	kWh/year	raw	R.S.A., T.M., and R.M.U.	27th October to 11th November. 2023	Not used in meta-analysis due number of effect sizes less than 3
25	Xiangfei Kong, Shilei Lu, Ping Gao, Neng Zhu, Wei Wu, Xuming Gao	2012	Energy and Buildings	Hainan Province, China	19.22	109.8	26	2009	Descriptive study	Descriptive statistics, Pearson correlations, Linear regression, benchmarking analyses and consumption indicators	Window type	0.05	>0,05	4.5	Second use of data from study 14	Secondary data	6000000	kWh/year	raw	R.S.A., T.M., and R.M.U.	27th October to 11th November. 2023	Not used in meta-analysis due number of effect sizes less than 3
26	Shiming Deng	2003	Energy and Buildings	Hong Kong	22.4	114.15	30	1996-1997	Descriptive study (considering only electricity)	Correlation analysis	floor area	0.81	NI	General	Main Study	Primary data	NI	GJ	raw	R.S.A., T.M., and R.M.U.	27th October to 11th November. 2023	Used in meta-analysis
26	Shiming Deng	2003	Energy and Buildings	Hong Kong	22.4	114.15	30	1996-1997	Descriptive study (considering only electricity)	Correlation analysis	Guestrooms	0.24	NI	General	Main Study	Primary data	NI	GJ	raw	R.S.A., T.M., and R.M.U.	27th October to 11th November. 2023	Used in meta-analysis
26	Shiming Deng	2003	Energy and Buildings	Hong Kong	22.4	114.15	30	1996-1997	Descriptive study (considering only electricity)	Correlation analysis	number of guests	0.23	NI	General	Main Study	Primary data	NI	GJ	raw	R.S.A., T.M., and R.M.U.	27th October to 11th November. 2023	Used in meta-analysis
27	Vlasta Zanki Alujevic´	2006	Royal Institute of Technology (Sweden)	Liburnia Rijeka - No seasonal	45.35	14.43	14	2003-2004	Descriptive study (considering only electricity)	Linear Regression and consumption indicators	Occupancy rate	0.124	NI	General	Main Study	Primary data	NI	kWh	raw	R.S.A., T.M., and R.M.U.	27th October to 11th November. 2023	Used in meta-analysis
27	Vlasta Zanki Alujevic´	2006	Royal Institute of Technology (Sweden)	Dubrovnik - Seasonal	42.65	18.1	12	2003-2004	Descriptive study (considering only electricity)	Linear Regression and consumption indicators	floor area	0.86	<0,01	General	Main Study	Primary data	NI	kWh	raw	R.S.A., T.M., and R.M.U.	27th October to 11th November. 2023	Not used in meta-analysis due sample number restriction
27	Vlasta Zanki Alujevic´	2006	Royal Institute of Technology (Sweden)	Dubrovnik - Seasonal	42.65	18.1	12	2003-2004	Descriptive study (considering only electricity)	Linear Regression and consumption indicators	Guestrooms	0.83	<0,01	General	Main Study	Primary data	NI	kWh	raw	R.S.A., T.M., and R.M.U.	27th October to 11th November. 2023	Not used in meta-analysis due sample number restriction
27	Vlasta Zanki Alujevic´	2006	Royal Institute of Technology (Sweden)	Istria - Seasonal	45.23	13.91	16	2003-2004	Descriptive study (considering only electricity)	Linear Regression and consumption indicators	floor area	0.66	>0,01	General	Main Study	Primary data	NI	kWh	raw	R.S.A., T.M., and R.M.U.	27th October to 11th November. 2023	Not used in meta-analysis due sample number restriction
27	Vlasta Zanki Alujevic´	2006	Royal Institute of Technology (Sweden)	Istria - Seasonal	45.23	13.91	16	2003-2004	Descriptive study (considering only electricity)	Linear Regression and consumption indicators	Guestrooms	0.40	>0,01	General	Main Study	Primary data	NI	kWh	raw	R.S.A., T.M., and R.M.U.	27th October to 11th November. 2023	Not used in meta-analysis due sample number restriction
27	Vlasta Zanki Alujevic´	2006	Royal Institute of Technology (Sweden)	Rijeka - seasonal	45.35	14.43	9	2003-2004	Descriptive study (considering only electricity)	Linear Regression and consumption indicators	floor area	0.86	<0,01	General	Main Study	Primary data	NI	kWh	raw	R.S.A., T.M., and R.M.U.	27th October to 11th November. 2023	Not used in meta-analysis due sample number restriction
27	Vlasta Zanki Alujevic´	2006	Royal Institute of Technology (Sweden)	Rijeka - seasonal	45.35	14.43	9	2003-2004	Descriptive study (considering only electricity)	Linear Regression and consumption indicators	Guestrooms	0.97	<0,01	General	Main Study	Primary data	NI	kWh	raw	R.S.A., T.M., and R.M.U.	27th October to 11th November. 2023	Not used in meta-analysis due sample number restriction
27	Vlasta Zanki Alujevic´	2006	Royal Institute of Technology (Sweden)	Split - seasonal	43.52	16.47	18	2003-2004	Descriptive study (considering only electricity)	Linear Regression and consumption indicators	floor area	0.70	>0,01	General	Main Study	Primary data	NI	kWh	raw	R.S.A., T.M., and R.M.U.	27th October to 11th November. 2023	Not used in meta-analysis due sample number restriction
27	Vlasta Zanki Alujevic´	2006	Royal Institute of Technology (Sweden)	Split - seasonal	43.52	16.47	18	2003-2004	Descriptive study (considering only electricity)	Linear Regression and consumption indicators	Guestrooms	0.60	>0,01	General	Main Study	Primary data	NI	kWh	raw	R.S.A., T.M., and R.M.U.	27th October to 11th November. 2023	Not used in meta-analysis due sample number restriction
27	Vlasta Zanki Alujevic´	2006	Royal Institute of Technology (Sweden)	Croatia - Seasonal	45.8	16	57	2003-2004	Descriptive study (considering only electricity)	Linear Regression and consumption indicators	floor area	0.68	>0,01	General	Main Study	Primary data	NI	kWh	raw	R.S.A., T.M., and R.M.U.	27th October to 11th November. 2023	Used in meta-analysis
27	Vlasta Zanki Alujevic´	2006	Royal Institute of Technology (Sweden)	Croatia - Seasonal	45.8	16	57	2003-2004	Descriptive study (considering only electricity)	Linear Regression and consumption indicators	Guestrooms	0.58	>0,01	General	Main Study	Primary data	NI	kWh	raw	R.S.A., T.M., and R.M.U.	27th October to 11th November. 2023	Used in meta-analysis
27	Vlasta Zanki Alujevic´	2006	Royal Institute of Technology (Sweden)	Dubrovnik - No Seasonal	42.65	18.1	6	2003-2004	Descriptive study (considering only electricity)	Linear Regression and consumption indicators	floor area	0.69	>0,01	General	Main Study	Primary data	NI	kWh	raw	R.S.A., T.M., and R.M.U.	27th October to 11th November. 2023	Not used in meta-analysis due sample number restriction
27	Vlasta Zanki Alujevic´	2006	Royal Institute of Technology (Sweden)	Dubrovnik - No Seasonal	42.65	18.1	6	2003-2004	Descriptive study (considering only electricity)	Linear Regression and consumption indicators	Guestrooms	0.74	>0,01	General	Main Study	Primary data	NI	kWh	raw	R.S.A., T.M., and R.M.U.	27th October to 11th November. 2023	Not used in meta-analysis due sample number restriction
27	Vlasta Zanki Alujevic´	2006	Royal Institute of Technology (Sweden)	Rijeka - No seasonal	45.35	14.43	21	2003-2004	Descriptive study (considering only electricity)	Linear Regression and consumption indicators	floor area	0.72	>0,01	General	Main Study	Primary data	NI	kWh	raw	R.S.A., T.M., and R.M.U.	27th October to 11th November. 2023	Not used in meta-analysis due sample number restriction
27	Vlasta Zanki Alujevic´	2006	Royal Institute of Technology (Sweden)	Rijeka - No seasonal	45.35	14.43	21	2003-2004	Descriptive study (considering only electricity)	Linear Regression and consumption indicators	Guestrooms	0.75	>0,01	General	Main Study	Primary data	NI	kWh	raw	R.S.A., T.M., and R.M.U.	27th October to 11th November. 2023	Not used in meta-analysis due sample number restriction
27	Vlasta Zanki Alujevic´	2006	Royal Institute of Technology (Sweden)	Split - No seasonal	43.52	16.47	5	2003-2004	Descriptive study (considering only electricity)	Linear Regression and consumption indicators	floor area	0.93	<0,01	General	Main Study	Primary data	NI	kWh	raw	R.S.A., T.M., and R.M.U.	27th October to 11th November. 2023	Not used in meta-analysis due sample number restriction
27	Vlasta Zanki Alujevic´	2006	Royal Institute of Technology (Sweden)	Split - No seasonal	43.52	16.47	5	2003-2004	Descriptive study (considering only electricity)	Linear Regression and consumption indicators	Guestrooms	0.77	>0,01	General	Main Study	Primary data	NI	kWh	raw	R.S.A., T.M., and R.M.U.	27th October to 11th November. 2023	Not used in meta-analysis due sample number restriction
27	Vlasta Zanki Alujevic´	2006	Royal Institute of Technology (Sweden)	Croatia - No seasonal	45.8	16	32	2003-2004	Descriptive study (considering only electricity)	Linear Regression and consumption indicators	floor area	0.60	>0,01	General	Main Study	Primary data	NI	kWh	raw	R.S.A., T.M., and R.M.U.	27th October to 11th November. 2023	Used in meta-analysis

27	Vlasta Zanki Alujevic´	2006	Royal Institute of Technology (Sweden)	Croatia - No seasonal	45.8	16	32	2003-2004	Descriptive study (considering only electricity)	Linear Regression and consumption indicators	Guestrooms	0.66	>0,01	General	Main Study	Primary data	NI	kWh	raw	R.S.A., T.M., and R.M.U.	27th October to 11th November. 2023	Used in meta-analysis
27	Vlasta Zanki Alujevic´	2006	Royal Institute of Technology (Sweden)	Croatia - Seasonal	45.8	16	4	2003-2004	Descriptive study (considering only electricity)	Linear Regression and consumption indicators	floor area	0.79	<0,01	4.5	Second use in the same study	Primary data	NI	kWh	raw	R.S.A., T.M., and R.M.U.	27th October to 11th November. 2023	Not used in meta-analysis due sample number restriction
27	Vlasta Zanki Alujevic´	2006	Royal Institute of Technology (Sweden)	Croatia - Seasonal	45.8	16	4	2003-2004	Descriptive study (considering only electricity)	Linear Regression and consumption indicators	Guestrooms	0.95	<0,01	4.5	Second use in the same study	Primary data	NI	kWh	raw	R.S.A., T.M., and R.M.U.	27th October to 11th November. 2023	Not used in meta-analysis due sample number restriction
27	Vlasta Zanki Alujevic´	2006	Royal Institute of Technology (Sweden)	Croatia - Seasonal	45.8	16	40	2003-2004	Descriptive study (considering only electricity)	Linear Regression and consumption indicators	floor area	0.69	>0,01	3	Second use in the same study	Primary data	NI	kWh	raw	R.S.A., T.M., and R.M.U.	27th October to 11th November. 2023	Not used in meta-analysis due sample number restriction
27	Vlasta Zanki Alujevic´	2006	Royal Institute of Technology (Sweden)	Croatia - Seasonal	45.8	16	40	2003-2004	Descriptive study (considering only electricity)	Linear Regression and consumption indicators	Guestrooms	0.73	>0,01	3	Second use in the same study	Primary data	NI	kWh	raw	R.S.A., T.M., and R.M.U.	27th October to 11th November. 2023	Not used in meta-analysis due sample number restriction
27	Vlasta Zanki Alujevic´	2006	Royal Institute of Technology (Sweden)	Croatia - Seasonal	45.8	16	13	2003-2004	Descriptive study (considering only electricity)	Linear Regression and consumption indicators	floor area	0.83	<0,01	2	Second use in the same study	Primary data	NI	kWh	raw	R.S.A., T.M., and R.M.U.	27th October to 11th November. 2023	Not used in meta-analysis due sample number restriction
27	Vlasta Zanki Alujevic´	2006	Royal Institute of Technology (Sweden)	Croatia - Seasonal	45.8	16	13	2003-2004	Descriptive study (considering only electricity)	Linear Regression and consumption indicators	Guestrooms	0.85	<0,01	2	Second use in the same study	Primary data	NI	kWh	raw	R.S.A., T.M., and R.M.U.	27th October to 11th November. 2023	Not used in meta-analysis due sample number restriction
27	Vlasta Zanki Alujevic´	2006	Royal Institute of Technology (Sweden)	Croatia - No seasonal	45.8	16	6	2003-2004	Descriptive study (considering only electricity)	Linear Regression and consumption indicators	floor area	0.07	>0,01	4.5	Second use in the same study	Primary data	NI	kWh	raw	R.S.A., T.M., and R.M.U.	27th October to 11th November. 2023	Not used in meta-analysis due sample number restriction
27	Vlasta Zanki Alujevic´	2006	Royal Institute of Technology (Sweden)	Croatia - No seasonal	45.8	16	6	2003-2004	Descriptive study (considering only electricity)	Linear Regression and consumption indicators	Guestrooms	0.04	>0,01	4.5	Second use in the same study	Primary data	NI	kWh	raw	R.S.A., T.M., and R.M.U.	27th October to 11th November. 2023	Not used in meta-analysis due sample number restriction
27	Vlasta Zanki Alujevic´	2006	Royal Institute of Technology (Sweden)	Croatia - No seasonal	45.8	16	20	2003-2004	Descriptive study (considering only electricity)	Linear Regression and consumption indicators	floor area	0.62	>0,01	3	Second use in the same study	Primary data	NI	kWh	raw	R.S.A., T.M., and R.M.U.	27th October to 11th November. 2023	Not used in meta-analysis due sample number restriction
27	Vlasta Zanki Alujevic´	2006	Royal Institute of Technology (Sweden)	Croatia - No seasonal	45.8	16	20	2003-2004	Descriptive study (considering only electricity)	Linear Regression and consumption indicators	Guestrooms	0.80	<0,01	3	Second use in the same study	Primary data	NI	kWh	raw	R.S.A., T.M., and R.M.U.	27th October to 11th November. 2023	Not used in meta-analysis due sample number restriction
27	Vlasta Zanki Alujevic´	2006	Royal Institute of Technology (Sweden)	Croatia - No seasonal	45.8	16	6	2003-2004	Descriptive study (considering only electricity)	Linear Regression and consumption indicators	floor area	0.83	<0,01	2	Second use in the same study	Primary data	NI	kWh	raw	R.S.A., T.M., and R.M.U.	27th October to 11th November. 2023	Not used in meta-analysis due sample number restriction
27	Vlasta Zanki Alujevic´	2006	Royal Institute of Technology (Sweden)	Croatia - No seasonal	45.8	16	6	2003-2004	Descriptive study (considering only electricity)	Linear Regression and consumption indicators	Guestrooms	0.53	>0,01	2	Second use in the same study	Primary data	NI	kWh	raw	R.S.A., T.M., and R.M.U.	27th October to 11th November. 2023	Not used in meta-analysis due sample number restriction
27	Vlasta Zanki Alujevic´	2006	Royal Institute of Technology (Sweden)	Liburnia Rijeka - No seasonal	45.35	14.43	14	2003-2004	Descriptive study (considering only electricity)	Linear Regression and consumption indicators	guest-nights	0.605	NI	General	Main Study	Primary data	NI	kWh	raw	R.S.A., T.M., and R.M.U.	27th October to 11th November. 2023	Used in meta-analysis
27	Vlasta Zanki Alujevic´	2006	Royal Institute of Technology (Sweden)	Dubrovnik - Seasonal	42.65	18.1	12	2003-2004	Descriptive study (considering only electricity)	Linear Regression and consumption indicators	floor area	0.27	>0,01	General	Second use in the same study	Primary data	75	kWh/m²	EUI	R.S.A., T.M., and R.M.U.	27th October to 11th November. 2023	Not used in meta-analysis due sample number restriction
27	Vlasta Zanki Alujevic´	2006	Royal Institute of Technology (Sweden)	Dubrovnik - Seasonal	42.65	18.1	12	2003-2004	Descriptive study (considering only electricity)	Linear Regression and consumption indicators	Guestrooms	0.12	>0,01	General	Second use in the same study	Primary data	75	kWh/m²	EUI	R.S.A., T.M., and R.M.U.	27th October to 11th November. 2023	Not used in meta-analysis due sample number restriction
27	Vlasta Zanki Alujevic´	2006	Royal Institute of Technology (Sweden)	Istria - Seasonal	45.23	13.91	16	2003-2004	Descriptive study (considering only electricity)	Linear Regression and consumption indicators	floor area	0.01	>0,01	General	Second use in the same study	Primary data	70	kWh/m²	EUI	R.S.A., T.M., and R.M.U.	27th October to 11th November. 2023	Not used in meta-analysis due sample number restriction
27	Vlasta Zanki Alujevic´	2006	Royal Institute of Technology (Sweden)	Istria - Seasonal	45.23	13.91	16	2003-2004	Descriptive study (considering only electricity)	Linear Regression and consumption indicators	Guestrooms	0.14	>0,01	General	Second use in the same study	Primary data	70	kWh/m²	EUI	R.S.A., T.M., and R.M.U.	27th October to 11th November. 2023	Not used in meta-analysis due sample number restriction
27	Vlasta Zanki Alujevic´	2006	Royal Institute of Technology (Sweden)	Rijeka - seasonal	45.35	14.43	9	2003-2004	Descriptive study (considering only electricity)	Linear Regression and consumption indicators	floor area	0.29	>0,01	General	Second use in the same study	Primary data	60	kWh/m²	EUI	R.S.A., T.M., and R.M.U.	27th October to 11th November. 2023	Not used in meta-analysis due sample number restriction
27	Vlasta Zanki Alujevic´	2006	Royal Institute of Technology (Sweden)	Rijeka - seasonal	45.35	14.43	9	2003-2004	Descriptive study (considering only electricity)	Linear Regression and consumption indicators	Guestrooms	0.71	>0,01	General	Second use in the same study	Primary data	60	kWh/m²	EUI	R.S.A., T.M., and R.M.U.	27th October to 11th November. 2023	Not used in meta-analysis due sample number restriction
27	Vlasta Zanki Alujevic´	2006	Royal Institute of Technology (Sweden)	Split - seasonal	43.52	16.47	18	2003-2004	Descriptive study (considering only electricity)	Linear Regression and consumption indicators	floor area	0.31	>0,01	General	Second use in the same study	Primary data	65	kWh/m²	EUI	R.S.A., T.M., and R.M.U.	27th October to 11th November. 2023	Not used in meta-analysis due sample number restriction
27	Vlasta Zanki Alujevic´	2006	Royal Institute of Technology (Sweden)	Split - seasonal	43.52	16.47	18	2003-2004	Descriptive study (considering only electricity)	Linear Regression and consumption indicators	Guestrooms	0.34	>0,01	General	Second use in the same study	Primary data	65	kWh/m²	EUI	R.S.A., T.M., and R.M.U.	27th October to 11th November. 2023	Not used in meta-analysis due sample number restriction
27	Vlasta Zanki Alujevic´	2006	Royal Institute of Technology (Sweden)	Croatia - Seasonal	45.8	16	57	2003-2004	Descriptive study (considering only electricity)	Linear Regression and consumption indicators	floor area	0.095	>0,01	General	Second use in the same study	Primary data	65	kWh/m²	EUI	R.S.A., T.M., and R.M.U.	27th October to 11th November. 2023	Used in meta-analysis
27	Vlasta Zanki Alujevic´	2006	Royal Institute of Technology (Sweden)	Croatia - Seasonal	45.8	16	57	2003-2004	Descriptive study (considering only electricity)	Linear Regression and consumption indicators	Guestrooms	0.07	>0,01	General	Second use in the same study	Primary data	65	kWh/m²	EUI	R.S.A., T.M., and R.M.U.	27th October to 11th November. 2023	Used in meta-analysis
27	Vlasta Zanki Alujevic´	2006	Royal Institute of Technology (Sweden)	Croatia - Seasonal	45.8	16	4	2003-2004	Descriptive study (considering only electricity)	Linear Regression and consumption indicators	floor area	0.21	>0,01	4.5	Second use in the same study	Primary data	65	kWh/m²	EUI	R.S.A., T.M., and R.M.U.	27th October to 11th November. 2023	Not used in meta-analysis due sample number restriction
27	Vlasta Zanki Alujevic´	2006	Royal Institute of Technology (Sweden)	Croatia - Seasonal	45.8	16	4	2003-2004	Descriptive study (considering only electricity)	Linear Regression and consumption indicators	Guestrooms	0.55	>0,01	4.5	Second use in the same study	Primary data	65	kWh/m²	EUI	R.S.A., T.M., and R.M.U.	27th October to 11th November. 2023	Not used in meta-analysis due sample number restriction
27	Vlasta Zanki Alujevic´	2006	Royal Institute of Technology (Sweden)	Croatia - Seasonal	45.8	16	40	2003-2004	Descriptive study (considering only electricity)	Linear Regression and consumption indicators	floor area	0.41	>0,01	3	Second use in the same study	Primary data	65	kWh/m²	EUI	R.S.A., T.M., and R.M.U.	27th October to 11th November. 2023	Not used in meta-analysis due sample number restriction
27	Vlasta Zanki Alujevic´	2006	Royal Institute of Technology (Sweden)	Croatia - Seasonal	45.8	16	40	2003-2004	Descriptive study (considering only electricity)	Linear Regression and consumption indicators	Guestrooms	0.26	>0,01	3	Second use in the same study	Primary data	65	kWh/m²	EUI	R.S.A., T.M., and R.M.U.	27th October to 11th November. 2023	Not used in meta-analysis due sample number restriction
27	Vlasta Zanki Alujevic´	2006	Royal Institute of Technology (Sweden)	Croatia - Seasonal	45.8	16	13	2003-2004	Descriptive study (considering only electricity)	Linear Regression and consumption indicators	floor area	0.20	>0,01	2	Second use in the same study	Primary data	65	kWh/m²	EUI	R.S.A., T.M., and R.M.U.	27th October to 11th November. 2023	Not used in meta-analysis due sample number restriction
27	Vlasta Zanki Alujevic´	2006	Royal Institute of Technology (Sweden)	Croatia - Seasonal	45.8	16	13	2003-2004	Descriptive study (considering only electricity)	Linear Regression and consumption indicators	Guestrooms	0.04	>0,01	2	Second use in the same study	Primary data	65	kWh/m²	EUI	R.S.A., T.M., and R.M.U.	27th October to 11th November. 2023	Not used in meta-analysis due sample number restriction
27	Vlasta Zanki Alujevic´	2006	Royal Institute of Technology (Sweden)	Dubrovnik - No Seasonal	42.65	18.1	6	2003-2004	Descriptive study (considering only electricity)	Linear Regression and consumption indicators	floor area	0.15	>0,01	General	Second use in the same study	Primary data	82	kWh/m²	EUI	R.S.A., T.M., and R.M.U.	27th October to 11th November. 2023	Not used in meta-analysis due sample number restriction
27	Vlasta Zanki Alujevic´	2006	Royal Institute of Technology (Sweden)	Dubrovnik - No Seasonal	42.65	18.1	6	2003-2004	Descriptive study (considering only electricity)	Linear Regression and consumption indicators	Guestrooms	0.55	>0,01	General	Second use in the same study	Primary data	82	kWh/m²	EUI	R.S.A., T.M., and R.M.U.	27th October to 11th November. 2023	Not used in meta-analysis due sample number restriction
27	Vlasta Zanki Alujevic´	2006	Royal Institute of Technology (Sweden)	Rijeka - No seasonal	45.35	14.43	21	2003-2004	Descriptive study (considering only electricity)	Linear Regression and consumption indicators	floor area	0.12	>0,01	General	Second use in the same study	Primary data	65	kWh/m²	EUI	R.S.A., T.M., and R.M.U.	27th October to 11th November. 2023	Not used in meta-analysis due sample number restriction
27	Vlasta Zanki Alujevic´	2006	Royal Institute of Technology (Sweden)	Rijeka - No seasonal	45.35	14.43	21	2003-2004	Descriptive study (considering only electricity)	Linear Regression and consumption indicators	Guestrooms	0.02	>0,01	General	Second use in the same study	Primary data	65	kWh/m²	EUI	R.S.A., T.M., and R.M.U.	27th October to 11th November. 2023	Not used in meta-analysis due sample number restriction
27	Vlasta Zanki Alujevic´	2006	Royal Institute of Technology (Sweden)	Split - No seasonal	43.52	16.47	5	2003-2004	Descriptive study (considering only electricity)	Linear Regression and consumption indicators	floor area	0.35	>0,01	General	Second use in the same study	Primary data	50	kWh/m²	EUI	R.S.A., T.M., and R.M.U.	27th October to 11th November. 2023	Not used in meta-analysis due sample number restriction
27	Vlasta Zanki Alujevic´	2006	Royal Institute of Technology (Sweden)	Split - No seasonal	43.52	16.47	5	2003-2004	Descriptive study (considering only electricity)	Linear Regression and consumption indicators	Guestrooms	0.35	>0,01	General	Second use in the same study	Primary data	50	kWh/m²	EUI	R.S.A., T.M., and R.M.U.	27th October to 11th November. 2023	Not used in meta-analysis due sample number restriction
27	Vlasta Zanki Alujevic´	2006	Royal Institute of Technology (Sweden)	Croatia - No seasonal	45.8	16	32	2003-2004	Descriptive study (considering only electricity)	Linear Regression and consumption indicators	floor area	0.10	>0,01	General	Second use in the same study	Primary data	65	kWh/m²	EUI	R.S.A., T.M., and R.M.U.	27th October to 11th November. 2023	Used in meta-analysis
27	Vlasta Zanki Alujevic´	2006	Royal Institute of Technology (Sweden)	Croatia - No seasonal	45.8	16	32	2003-2004	Descriptive study (considering only electricity)	Linear Regression and consumption indicators	Guestrooms	0.11	>0,01	General	Second use in the same study	Primary data	65	kWh/m²	EUI	R.S.A., T.M., and R.M.U.	27th October to 11th November. 2023	Used in meta-analysis
27	Vlasta Zanki Alujevic´	2006	Royal Institute of Technology (Sweden)	Croatia - No seasonal	45.8	16	6	2003-2004	Descriptive study (considering only electricity)	Linear Regression and consumption indicators	floor area	0.53	>0,01	4.5	Second use in the same study	Primary data	65	kWh/m²	EUI	R.S.A., T.M., and R.M.U.	27th October to 11th November. 2023	Not used in meta-analysis due sample number restriction
27	Vlasta Zanki Alujevic´	2006	Royal Institute of Technology (Sweden)	Croatia - No seasonal	45.8	16	6	2003-2004	Descriptive study (considering only electricity)	Linear Regression and consumption indicators	Guestrooms	0.34	>0,01	4.5	Second use in the same study	Primary data	65	kWh/m²	EUI	R.S.A., T.M., and R.M.U.	27th October to 11th November. 2023	Not used in meta-analysis due sample number restriction
27	Vlasta Zanki Alujevic´	2006	Royal Institute of Technology (Sweden)	Croatia - No seasonal	45.8	16	20	2003-2004	Descriptive study (considering only electricity)	Linear Regression and consumption indicators	floor area	0.19	>0,01	3	Second use in the same study	Primary data	65	kWh/m²	EUI	R.S.A., T.M., and R.M.U.	27th October to 11th November. 2023	Not used in meta-analysis due sample number restriction
27	Vlasta Zanki Alujevic´	2006	Royal Institute of Technology (Sweden)	Croatia - No seasonal	45.8	16	20	2003-2004	Descriptive study (considering only electricity)	Linear Regression and consumption indicators	Guestrooms	0.16	>0,01	3	Second use in the same study	Primary data	65	kWh/m²	EUI	R.S.A., T.M., and R.M.U.	27th October to 11th November. 2023	Not used in meta-analysis due sample number restriction
27	Vlasta Zanki Alujevic´	2006	Royal Institute of Technology (Sweden)	Croatia - No seasonal	45.8	16	6	2003-2004	Descriptive study (considering only electricity)	Linear Regression and consumption indicators	floor area	0.03	>0,01	2	Second use in the same study	Primary data	65	kWh/m²	EUI	R.S.A., T.M., and R.M.U.	27th October to 11th November. 2023	Not used in meta-analysis due sample number restriction
27	Vlasta Zanki Alujevic´	2006	Royal Institute of Technology (Sweden)	Croatia - No seasonal	45.8	16	6	2003-2004	Descriptive study (considering only electricity)	Linear Regression and consumption indicators	Guestrooms	0.10	>0,01	2	Second use in the same study	Primary data	65	kWh/m²	EUI	R.S.A., T.M., and R.M.U.	27th October to 11th November. 2023	Not used in meta-analysis due sample number restriction
28	Chukwudi Okpala, Howard Njoku, Paul Ako	2023	Engineering Proceedings	Nsukka, Nigeria	6.8	7.4	10	2021	Descriptive study (considering only electricity)	Correlation analysis, consumption indicators and Data Envelopment Analysis	Occupancy rate	0.54	<0,05	General	Main Study	Primary data	51.42	kWh/m²	EUI	R.S.A.	14th Mai, 2024	Used in meta-analysis
28	Chukwudi Okpala, Howard Njoku, Paul Ako	2023	Engineering Proceedings	Nsukka, Nigeria	6.8	7.4	10	2021	Descriptive study (considering only electricity)	Correlation analysis, consumption indicators and Data Envelopment Analysis	Number of employees	0.03	>0,05	General	Main Study	Primary data	51.42	kWh/m²	EUI	R.S.A.	14th Mai, 2024	Used in meta-analysis
