# Supplementary material for: Energy use and its contributors in hotel buildings: A systematic review and meta-analysis
Source: PLoS One. 2024 Oct 24;19(10):e0309745. doi: 10.1371/journal.pone.0309745 (PMC11500959; doi:10.1371/journal.pone.0309745)
Supplement: S2 Table — (PDF) [file pone.0309745.s003.pdf]

S3 Table. Independent variables retrieved from the included studies

Dimension	Variable	Effect sizes
Building characteristics	Floor area	30
Building characteristics	Guestrooms	21
Building characteristics	Star level	5
Building characteristics	Construction year	5
Building characteristics	Building age	4
Building characteristics	Number of floors	4
Building characteristics	Guestrooms area	3
Building characteristics	Retrofit	3
Economics	Occupancy	21
Economics	Guest-nights	8
Economics	Number of guests	5
Economics	Food covers	4
Economics	Room revenue	4
Social	Number of employees	7
Social	Employees density	4
environmental	Carbon emissions	3
environmental	Outdoor temperature	3
environmental	Water use	3
Building characteristics	GFA/guestrooms	2
Building characteristics	Building area	2
Economics	Operation cost	2
Economics	revenue	2
Economics	food and beverage revenue	2
Building characteristics	laundry	1
Building characteristics	5 star	1
Building characteristics	4 star	1
Building characteristics	3 star	1
Building characteristics	restaurants	1
Building characteristics	Garden area	1
Building characteristics	pools (volume)	1
Building characteristics	pools (area)	1
Building characteristics	pools (temperature)	1
Building characteristics	aircon	1
Building characteristics	spa	1
Building characteristics	Kitchenette in room	1
Building characteristics	travelife certified	1
Building characteristics	addicional services areas	1
Building characteristics	group of guests	1
Building characteristics	number of beds	1
Building characteristics	Building stories	1
Building characteristics	(%) of GFA with dining facilities	1
Building characteristics	(%) of GFA with retail shops	1
Building characteristics	GFA/AIRCCOND	1
Building characteristics	Illumination of guestrooms	1
Building characteristics	central air cond	1
Building characteristics	external wall type	1
Building characteristics	window type	1
Economics	Construction cost	1
Economics	Maintenance cost replacement	1
Economics	custo total	1
Economics	Maintenance cost	1
Economics	rank of overnight	1
Economics	Net return	1
Economics	number of diners	1
Economics	ADR	1
Economics	number of repairs	1
Economics	refrigerants reposition	1
Economics	equivalent rooms	1

environmental	Carbon emissions construction	1
environmental	Carbon emission operation	1
environmental	Carbon emissions replacement	1
environmental	Loss rate	1
environmental	energy audit	1
environmental	cold water temperature	1
environmental	renewable energy	1
environmental	recycled kitchen oil	1
environmental	recycled cardboard*	1
environmental	cooling temperature	1
environmental	heating degree days	1
environmental	RDD	1
Social	Tourist number	1
Social	workers per shift	1
Social	average room rate	1
Social	guest per room	1
Social	GRI	1
Social	number of adult guests	1
Social	number of children guests	1
Social	bangkok	1
Social	location district	1
Social	guest of foreing independent tourist	1
Social	guest of group	1
Social	guests from taiwan	1
Social	guests from china	1
Social	guests from north america	1
Social	guests from japan	1
Social	guest from europe	1
