# Supplementary material for: Energy use and its contributors in hotel buildings: A systematic review and meta-analysis
Source: PLoS One. 2024 Oct 24;19(10):e0309745. doi: 10.1371/journal.pone.0309745 (PMC11500959; doi:10.1371/journal.pone.0309745)
Supplement: S3 Table — (PDF) [file pone.0309745.s004.pdf]

S6 Table. Subgroup analyses for the three variables with more than 10 studies

Contributor	Moderators	k	Subgroup	r	95%-CI	Q	T	I² (%)	p-value
Floor area	Statiscal significance	13	Significant	0.7600	[0.5379; 0.8834]	260.80	0.6866	95.4	0.1213
		17	No significant	0.5417	[-0.3022; 0.7169]	255.80	0.5896	93.7	
	Hotel category	25	General	0.6666	[0.4909; 0.7903]	480.2	0.6460	95.0	0.4835
		5	Upscale	0.5391	[0.1054; 0.8005]	51.65	0.5412	92.3	
	Type of energy indicator	18	raw energy	0.8419	[0.7887; 0.8826]	72.42	0.2823	76.5	**<0.0001**
		12	EUI	0.1359	[-0.0098; 0.2759]	30.79	0.1942	64.3	
	Main method	25	Regression	0.6408	[0.4603; 0.7704]	491.99	0.6326	95.1	0.8502
		5	Correlation	0.6743	[0.2593; 0.8791]	43.89	0.6004	90.9	
	Journal quality	7	A (jcr > 2)	0.5513	[-0.0893; 0.8693]	77.36	0.9125	92.2	0.8835
		12	B (> 1 jcr < 2)	0.6763	[0.4035; 0.8386]	368.84	0.6795	97.0	
		11	C (jcr < 1)	0.6626	[0.4723; 0.7939]	78.95	0.4370	87.3	
	Climate Zone	4	Equatorial	0.6266	[-0.1153; 0.9198]	57.77	0.8462	94.8	0.7592
		8	Tropical	0.7639	[0.4483; 0.9101]	219.10	0.7186	96.8	
		8	Sub-tropical	0.5950	[0.0683; 0.8623]	86.34	0.8450	91.9	
		10	Temperate	0.5867	[0.3317; 0.7618]	150.96	0.5105	94.0	
	Continent zone	17	Asia	0.6905	[0.4483; 0.8382]	353.58	0.7362	95.5	0.7685
		10	Europe	0.5867	[0.3317; 0.7618]	150.96	0.5105	94.0	
		3	Africa	0.5829	[-0.2105; 0.9133]	16.44	0.7027	87.8	
	Energy source	25	Electricity	0.6307	[0.4451; 0.7643]	497.92	0.6398	95.2	0.7838
		3	Diesel	0.7098	[-0.1074; 0.9546]	25.99	0.8374	92.3	
		2	Gas	0.7448	[-0.3590; 0.9132]	8.56	0.3969	88.3	
	Decade years	16	2000	0.6254	[0.3883; 0.7849]	244.07	0.6254	93.9	0.6562
		11	2010	0.6387	[0.3150; 0.8293]	274.03	0.7054	96.4	
		3	2020	0.7635	[0.4656; 0.9060]	8.84	0.3615	77.4	
Guestrooms	Statiscal significance	8	Significant	0.7005	[0.3672; 0.8744]	174.79	0.6570	96.0	0.0678
		13	No significant	0.3619	[0.1720; 0.5259]	61.27	0.3332	80.4	
	Hotel category	16	General	0.5605	[0.3317; 0.7271]	268.08	0.5575	94.4	0.1566
		5	Upscale	0.2844	[-0.0807; 0.5822]	15.50	0.3655	74.2	
	Type of energy indicator	13	raw energy	0.6614	[0.4596; 0.7983]	153.69	0.5104	92.2	**0.0004**
		8	EUI	0.1785	[0.0195; 0.3287]	13.75	0.1551	49.1	
	Main method	15	Regression	0.5863	[-0.0002; 0.4876]	253.30	0.5842	94.5	0.0528
		6	Correlation	0.2602	[0.3449; 0.7550]	16.42	0.2706	69.6	
	Journal quality	13	A (jcr > 2)	0.4747	[0.1486; 0.7076]	268.72	0.6479	95.5	0.6948
		8	B (jcr < 2)	0.5417	[0.3287; 0.7023]	33.97	0.3275	79.4	
	Climate Zone	4	Equatorial	0.5488	[0.0828; 0.8179]	22.69	0.5073	86.8	**0.0302**
		9	Tropical	0.6267	[0.2917; 0.8248]	202.44	0.6275	96.0	
		3	Sub-tropical	0.0674	[-0.1980; 0.3236]	3.03	0.1383	34.1	
		5	Temperate	0.4534	[0.1635; 0.6712]	22.77	0.3342	82.4	
	Continent zone	12	Asia	0.5238	[0.1855; 0.7511]	250.56	0.6705	95.6	0.9371
		5	Europe	0.4534	[0.1635; 0.6712]	22.77	0.3342	82.4	
		4	Africa	0.4725	[0.1585; 0.6997]	9.32	0.2798	67.8	
	Energy source	18	Electricity	0.5025	[0.2634; 0.6835]	293.77	0.5782	94.2	0.9297
		3	Diesel or Gas	0.5212	[0.0927; 0.7868]	11.29	0.3880	82.3	
	Decade years	7	2000	0.4520	[0.1760; 0.6621]	35.51	0.3787	83.1	0.7416
		11	2010	0.5021	[0.1311; 0.7497]	244.79	0.6857	95.9	
		3	2020	0.5729	[0.3512; 0.7337]	4.17	0.1749	52.1	
Occupancy	Statiscal significance	8	Significant	0.2417	[-0.1619; 0.5760]	119.52	0.5408	94.1	0.8202
		13	No significant	0.1896	[0.0414; 0.4013]	50.75	0.3558	76.4	
	Hotel category	15	General	0.3557	[0.1153; 0.5567]	149.27	0.4513	90.6	**0.0099**
		6	Upscale	-0.2125	[-0.5237; 0.1487]	17.54	0.3741	71.5	
	Type of energy indicator	12	raw energy	0.2411	[-0.0638; 0.5048]	113.19	0.4921	90.3	0.6770
		9	EUI	0.1371	[-0.2582; 0.4931]	86.70	0.5608	90.8	
	Main method	14	Regression	0.3068	[0.0376; 0.5345]	124.33	0.4692	89.5	**0.0456**
		7	Correlation	-0.0363	[-0.2365; 0.1669]	13.13	0.0374	54.3	
	Journal quality	4	A (jcr > 2)	0.1703	[-0.4800; 0.6998]	26.37	0.6616	88.6	0.3759
		10	B (> 1 jcr < 2)	0.3284	[0.0545; 0.5563]	105.83	0.4257	91.5	
		7	C (jcr < 1)	0.0147	[-0.3308; 0.3566]	21.42	0.3750	72.0	
	Climate Zone	5	Equatorial	0.3258	[-0.0894; 0.6445]	19.56	0.4287	79.6	0.9198
		11	Tropical	0.1685	[-0.1491; 0.4546]	144.52	0.4868	93.1	
		4	Sub-tropical	0.1703	[-0.4800; 0.6998]	26.37	0.6616	88.6	
		1	Temperate	0.1240	[-0.4352; 0.6142]	NA	NA	NA	
	Continent zone	14	Asia	0.2969	[0.0271; 0.5264]	153.96	0.4929	91.6	0.2874
		1	Europe	0.1240	[-0.4352; 0.6142]	NA	NA	NA	
		6	Africa	-0.0407	[-0.3541; 0.2808]	13.54	0.2949	63.1	
	Energy source	19	Electricity	0.2296	[-0.0152; 0.4484]	197.75	0.4948	90.9	0.1428
		2	Diesel	-0.0449	[-0.3113; 0.2282]	0.36	0.0000	0.0	
	Decade years	5	2000	0.5442	[0.2191; 0.7605]	12.24	0.3572	67.3	0.0749
		13	2010	0.0439	[-0.2625; 0.3423]	170.34	0.5337	93.00	
		3	2020	0.1593	[-0.2145; 0.4923]	2.85	0.2125	29.9	
