# Supplementary material for: Energy use and its contributors in hotel buildings: A systematic review and meta-analysis
Source: PLoS One. 2024 Oct 24;19(10):e0309745. doi: 10.1371/journal.pone.0309745 (PMC11500959; doi:10.1371/journal.pone.0309745)
Supplement: S4 Table — (PDF) [file pone.0309745.s005.pdf]

S7 Table. Complete cumulative meta-analyses

Cumulative meta-analysis of floor area per energy data type (raw to EUI)						
Added study	k	r	95% C.I.	p-value	T	I^2 (%)
Chan and Lam (2002) a	1	0.9350	[0.8252; 0.9767]	<0.0001	NA	NA
Chan and Lam (2002) b	2	0.9296	[0.8447; 0.9688]	<0.0001	0.0000	0.0
Deng (2003)	3	0.8883	[0.7718; 0.9471]	<0.0001	0.2242	42.0
Chan and Mak (2004)	4	0.9020	[0.8118; 0.9502]	<0.0001	0.2184	37.3
Alujevic´ (2006) a	5	0.8709	[0.7331; 0.9401]	<0.0001	0.3760	72.4
Alujevic´ (2006) b	6	0.8366	[0.6947; 0.9158]	<0.0001	0.3644	73.5
Bohdanowicz and Martinac (2007) a	7	0.8323	[0.7252; 0.9001]	<0.0001	0.2992	70.8
Bohdanowicz and Martinac (2007) b	8	0.8373	[0.7531; 0.8945]	<0.0001	0.2652	71.5
Priyadarsini et al. (2009) a	9	0.8597	[0.7814; 0.9114]	<0.0001	0.3091	76.9
Wang (2012) a	10	0.8700	[0.8019; 0.9158]	<0.0001	0.3137	82.4
Kong et al. (2012)	11	0.8624	[0.7949; 0.9088]	<0.0001	0.3121	81.6
Lai (2016) a	12	0.8477	[0.7714; 0.8999]	<0.0001	0.3424	83.8
Oluseyi et al. (2016) a	13	0.8424	[0.7686; 0.8940]	<0.0001	0.3364	82.9
Tangon et al. (2018)	14	0.8401	[0.7726; 0.8888]	<0.0001	0.3181	81.8
Nguyen and Rockwood (2019) a	15	0.8378	[0.7730; 0.8853]	<0.0001	0.3117	80.6
Nguyen and Rockwood (2019) b	16	0.8412	[0.7823; 0.8852]	<0.0001	0.2991	79.3
Santiago (2021)	17	0.8414	[0.7838; 0.8847]	<0.0001	0.2957	77.9
Stimac et al. (2023) a	18	0.8419	[0.7887; 0.8826]	<0.0001	0.2823	76.5
Chan and Lam (2002) c	19	0.8202	[0.7483; 0.8731]	<0.0001	0.3622	83.8
Chan and Lam (2002) d	20	0.7955	[0.7063; 0.8598]	<0.0001	0.4156	86.8
Alujevic´ (2006) c	21	0.7735	[0.6623; 0.8513]	<0.0001	0.4944	90.5
Alujevic´ (2006) d	22	0.7548	[0.6329; 0.8401]	<0.0001	0.5230	91.3
Bohdanowicz and Martinac (2007) c	23	0.7402	[0.6104; 0.8313]	<0.0001	0.5482	92.3
Bohdanowicz and Martinac (2007) c	24	0.7235	[0.5806; 0.8231]	<0.0001	0.5896	93.7
Priyadarsini et al. (2009) b	25	0.7086	[0.5622; 0.8119]	<0.0001	0.5949	93.7
Wang (2012) b	26	0.6932	[0.5375; 0.8031]	<0.0001	0.6234	94.9
Lai (2016) b	27	0.6730	[0.5111; 0.7887]	<0.0001	0.6326	94.9
Oluseyi et al. (2016) b	28	0.6551	[0.4893; 0.7752]	<0.0001	0.6376	94.9
Tang et al. (2016)	29	0.6489	[0.4856; 0.7684]	<0.0001	0.6328	94.8
Stimac et al. (2023) b	30	0.6465	[0.4890; 0.7632]	<0.0001	0.6207	94.6

Cumulative meta-analysis of number of guestrooms per sample size						
Added study	k	r	95% C.I.	p-value	T	I² (%)
Santiago (2021)	1	0.7940	[-0.0494; 0.9764]	0.0609	NA	NA
Xin et al. (2012) a	2	0.7049	[0.4031; 0.8684]	0.0001	0.0000	0.0
Xin et al. (2012) b	3	0.5197	[-0.0490; 0.8339]	0.0709	0.4357	65.6
Tang et al. (2016)	4	0.5323	[0.1882; 0.7600]	0.0039	0.2896	52.0
Kong et al. (2012)	5	0.5383	[0.3031; 0.7117]	<0.0001	0.1977	37.0
Oluseyi et al. (2016) a	6	0.5761	[0.3902; 0.7168]	<0.0001	0.1766	34.3
Oluseyi et al. (2016) b	7	0.5042	[0.2631; 0.6860]	0.0001	0.2923	60.5
Priyadarsini et al. (2009) a	8	0.5783	[0.3311; 0.7512]	<0.0001	0.3807	73.4
Priyadarsini et al. (2009) b	9	0.5480	[0.3177; 0.7173]	<0.0001	0.3646	72.5
Deng (2003)	10	0.5180	[0.2997; 0.6848]	<0.0001	0.3543	72.0
Lai (2016) a	11	0.4867	[0.2753; 0.6533]	<0.0001	0.3513	72.3
Lai (2016) b	12	0.4362	[0.2042; 0.6218]	0.0004	0.3973	77.4
Alujevic´ (2006) c	13	0.4576	[0.2453; 0.6281]	<0.0001	0.3863	76.8
Alujevic´ (2006) d	14	0.4334	[0.2292; 0.6011]	<0.0001	0.3787	76.5
Stimac et al. (2023)	15	0.4546	[0.2658; 0.6098]	<0.0001	0.3710	77.0
Alujevic´ (2006) a	16	0.4641	[0.2939; 0.6058]	<0.0001	0.3475	75.9
Alujevic´ (2006) b	17	0.4405	[0.2703; 0.5840]	<0.0001	0.3546	77.7
Tangon et al. (2018)	18	0.4658	[0.3005; 0.6040]	<0.0001	0.3680	79.9
Mechri and Amara (2021)	19	0.4644	[0.3166; 0.5901]	<0.0001	0.3347	78.7
Wang (2012) a	20	0.5151	[0.2929; 0.6845]	<0.0001	0.5762	93.0
Wang (2012) b	21	0.5047	[0.2952; 0.6679]	<0.0001	0.5531	93.5

Cumulative meta-analysis of hotels occupancy per sample size						
Added study	k	r	95% C.I.	p-value	T	I² (%)
Perez et al. (2016)	1	-0.9497	[-0.9946; -0.6025]	0.0015	NA	NA
Santiago (2021)	2	-0.6090	[-0.9940; 0.9035]	0.5282	1.4771	86.7
Okpala et al. (2023)	3	-0.2316	[-0.9357; 0.8428]	0.7526	1.1895	84.7
Chan and Lam (2002) a	4	0.0029	[-0.7556; 0.7581]	0.9955	0.8911	79.6
Alujevic´ (2006)	5	0.0560	[-0.5668; 0.6381]	0.8750	0.6686	72.9
Chan and Lam (2002) b	6	0.2424	[-0.3704; 0.7082]	0.4462	0.6828	77.2
Xin et al. (2012) a	7	0.2211	[-0.2904; 0.6343]	0.4003	0.5952	74.9
Xin et al. (2012) b	8	0.2559	[-0.1683; 0.6002]	0.2347	0.5082	70.8
Tang et al. (2016)	9	0.2918	[-0.0604; 0.5793]	0.1027	0.4363	66.9
Kong et al. (2012)	10	0.2647	[-0.0490; 0.5308]	0.097	0.4034	65.8
Oluseyi et al. (2016) a	11	0.2216	[-0.0712; 0.4793]	0.1366	0.3948	66.9
Oluseyi et al. (2016) b	12	0.2054	[-0.0561; 0.4406]	0.1226	0.3608	64.5
Priyadarsini et al. (2009) a	13	0.2704	[-0.0067; 0.5090]	0.0556	0.4311	73.4
Priyadarsini et al. (2009) b	14	0.2722	[-0.0228; 0.4897]	0.0328	0.3977	71.3
Lai (2016) a	15	0.2175	[-0.0413; 0.4489]	0.0987	0.4350	75.7
Lai (2016) b	16	0.1737	[-0.0830; 0.4088]	0.1836	0.4492	77.5
Nguyen and Rockwood (2019)	17	0.1624	[-0.0711; 0.3790]	0.1718	0.4159	76.4
Tangon et al. (2018)	18	0.1333	[-0.0890; 0.3430]	0.2392	0.4079	77.6
Mechri and Amara (2021)	19	0.1233	[-0.0713; 0.3090]	0.2137	0.3578	76.5
Wang (2012) a	20	0.1632	[-0.0616; 0.3723]	0.1538	0.4533	86.9
Wang (2012) b	21	0.2029	[-0.0276; 0.4129]	0.0839	0.4882	90.4
