# Supplementary material for: Energy use and its contributors in hotel buildings: A systematic review and meta-analysis
Source: PLoS One. 2024 Oct 24;19(10):e0309745. doi: 10.1371/journal.pone.0309745 (PMC11500959; doi:10.1371/journal.pone.0309745)
Supplement: S5 Table — (PDF) [file pone.0309745.s006.pdf]

S8 Table. Estimate of cumulative evidence from the studies included in meta-analysis

Number	Study	Year	Study site	Sample size	Theory support	Period of analysis	Type of data	Cumulative evidence	Observation
1	Silu Bhochhibhoya, Massimo Pizzol, Francesco Marinello, Raffaele Cavalli	2020	10	2.3	10	5	5	NA	Not used in meta-analysis
2	Hyerim Yoon, David Sauri, Antonio Rico	2021	10	0.6	10	5	10	71%	Used in meta-analysis
3	Rodrigo Schons Arenhart, Adriano Mendonça Souza, Roselaine Ruviaro Zanini	2022	1	2.3	5	5	5	37%	Used in meta-analysis
4	Anh Tuan Nguyen, David Rockwood	2019	10	2.5	5	5	10	65%	Used in meta-analysis
5	Joseph H. K. Lai	2016	5	1.5	5	5	10	53%	Used in meta-analysis
6	Wu Xuchao, Rajagopalan Priyadarsini, Lee Siew Eang	2010	5	1.5	5	5	5	NA	Not used in meta-analysis
7	P. O. Oluseyi, O. M. Babatunde, O. A. Babatunde	2016	10	1.4	5	5	10	63%	Used in meta-analysis
8	Dunia E. Santiago	2021	8	0.3	5	5	10	57%	Used in meta-analysis
9	Wilco W. Chan, Barry L. Mak	2004	5	0.5	5	10	5	51%	Used in meta-analysis
10	Wilco W. Chan, Joseph C. Lam	2002	5	0.9	5	10	5	52%	Used in meta-analysis
11	Suwajee Tangon, Jaruwan Chontanawat, Siriluk Chiarakorn	2018	5	3.2	5	5	10	56%	Used in meta-analysis
12	Francisco Javier Diaz Perez, David Chinarro, Adib Guardiola Mouhaffel, Ricardo Diaz Martin, Rosa Pino Otin	2016	8	0.3	5	10	10	67%	Used in meta-analysis
13	Yajuan Xin, Shilei Lu, Neng Zhu, Wei Wu	2012	8	1.0	10	5	10	68%	Used in meta-analysis
14	Mingfang Tang, Xiao Fu, Huiming Cao, Yuan Shen, Hongbing Deng, Gang Wu	2016	8	1.2	5	5	10	58%	Used in meta-analysis
15	Rajagopalan Priyadarsini, Wu Xuchao, Lee Siew Eang	2009	5	1.5	5	5	10	53%	Used in meta-analysis
16	Paulina Bohdanowicz, Ivo Martinac	2007	3	5.6	5	5	5	47%	Used in meta-analysis
17	Jen Chun Wang, Kuo-Tsang Huang	2013	5	1.7	5	5	10	53%	Used in meta-analysis
18	Maja Štimac, Mario Matkovic, Daria Karasalihovic Sedlar	2023	5	2.6	5	10	10	65%	Used in meta-analysis
19	Silu Bhochhibhoya	2016	10	2.3	10	5	10	75%	Used in meta-analysis
20	Houcem Eddine Mechri, Samir Amara	2021	5	6.7	5	5	10	63%	Used in meta-analysis
21	Jen Chun Wang	2012	5	10.0	5	5	10	70%	Used in meta-analysis
22	George Papageorgiou, Andreas Efstathiades, Nicolas Nicolaou, Athanasios Maimaris	2018	5	0.6	5	5	10	51%	Used in meta-analysis
23	Wilco W. Chan, Joseph C. Lam	2002	5	0.6	5	10	10	61%	Used in meta-analysis
24	Juan José Cabello Eras, Vladimir Sousa Santos, Alexis Sagastume Gutiérrez, Mario Álvarez Guerra Plasencia, Dries Haeseldonckx, Carlo Vandecasteele	2016	8	0.1	5	5	10	NA	Not used in meta-analysis
25	Xiangfei Kong, Shilei Lu, Ping Gao, Neng Zhu, Wei Wu, Xuming Gao	2012	8	1.3	5	5	5	49%	Used in meta-analysis
26	Shiming Deng	2003	5	1.5	5	10	10	63%	Used in meta-analysis
27	Vlasta Zanki Alujevic´	2006	8	2.9	5	5	10	62%	Used in meta-analysis
28	Chukwudi Okpala, Howard Njoku, Paul Ako	2023	10	0.5	5	5	10	61%	Used in meta-analysis
				**Cumulative Evidence**				**59%**	
