# Supplementary material for: Energy use and its contributors in hotel buildings: A systematic review and meta-analysis
Source: PLoS One. 2024 Oct 24;19(10):e0309745. doi: 10.1371/journal.pone.0309745 (PMC11500959; doi:10.1371/journal.pone.0309745)
Supplement: S1 Appendix — (PDF) [file pone.0309745.s007.pdf]

S4 Appendix. Meta-analyses coding book

```
# Find the repository:
getwd()

# Meta-analysis of Floor area relationships with energy use in hotels

# read ".csv" file:
dados <- read.csv("FloorArea.csv", as.is=TRUE)
# Check data:
dados
# Load meta and metafor packages:
library(meta)
library(metafor)
# correlation meta-analysis:
c <- metacor (cor=r, n=n, studlab=paste(Authors, Year), data=dados, fixed=FALSE,
random=TRUE, method.tau="DL")
summary(c)
# forest plot of the meta-analysis:
forest(c, xlim=c(-1,1))
# subgroup meta-analysis:
csg1 <- update (c, byvar=sig, print.byvar=FALSE)
summary(csg1)
csg2 <- update (c, byvar=category, print.byvar=FALSE)
summary(csg2)
csg3 <- update (c, byvar=energy, print.byvar=FALSE)
summary(csg3)
csg4 <- update (c, byvar=method, print.byvar=FALSE)
summary(csg4)
csg5 <- update (c, byvar=sjrabc, print.byvar=FALSE)
summary(csg5)
csg6 <- update (c, byvar=climaticalz, print.byvar=FALSE)
summary(csg6)
csg7 <- update (c, byvar=continent, print.byvar=FALSE)
summary(csg7)
csg8 <- update (c, byvar=predsource, print.byvar=FALSE)
summary(csg8)
csg9 <- update (c, byvar=decade, print.byvar=FALSE)
summary(csg9)
forest(csg3, xlim=c(-1,1))
# Meta-regression analyses:
cmr1 <- metareg(c, Latitude)
print(cmr1)
cmr2 <- metareg(c, Longitude)
print(cmr2)
cmr3 <- metareg(c, sig)
print(cmr3)
cmr4 <- metareg(c, category)
print(cmr4)
cmr5 <- metareg(c, energy)
print(cmr5)
cmr6 <- metareg(c, sjr)
print(cmr6)
cmr7 <- metareg(c, Year)
```

```
print(cmr7)
cmr8 <- metareg(c, energy + Year)
print(cmr8)
bubble(cmr5)
# Publication Bias - funnel plot:
funnel(c)
# funnel plot simetry tests:
metabias(c, method="rank")
metabias(c, method="linreg")
#FSN:
fsn(yi=z, vi=vi, data=dados, type="Orwin", target=0.01)
fsn(yi=z, vi=vi, data=dados, type="Rosenthal")
# Cumulative Meta-analysis:
metacum(c, pooled="random", sortvar=-energy)
forest(metacum(c), xlim=c(-1,1))


# Meta-analysis of Guestrooms relationships with energy use in hotels

# read ".csv" file:
dados <- read.csv("Guestrooms.csv", as.is=TRUE)
# Check data:
dados
# correlation meta-analysis:
c <- metacor (cor=r, n=n, studlab=paste(Authors, Year), data=dados, fixed=FALSE,
random=TRUE, method.tau="DL")
summary(c)
# forest plot of the meta-analysis:
forest(c, xlim=c(-1,1))
# subgroup meta-analysis:
csg1 <- update (c, byvar=sig, print.byvar=FALSE)
summary(csg1)
csg2 <- update (c, byvar=category, print.byvar=FALSE)
summary(csg2)
csg3 <- update (c, byvar=energy, print.byvar=FALSE)
summary(csg3)
csg4 <- update (c, byvar=method, print.byvar=FALSE)
summary(csg4)
csg5 <- update (c, byvar=sjrab, print.byvar=FALSE)
summary(csg5)
csg6 <- update (c, byvar=climatez, print.byvar=FALSE)
summary(csg6)
csg7 <- update (c, byvar=continent, print.byvar=FALSE)
summary(csg7)
csg8 <- update (c, byvar=predsource, print.byvar=FALSE)
summary(csg8)
csg9 <- update (c, byvar=decade, print.byvar=FALSE)
summary(csg9)
forest(csg3, xlim=c(-1,1))
# Meta-regression analyses:
cmr1 <- metareg(c, Latitude)
print(cmr1)
cmr2 <- metareg(c, Longitude)
print(cmr2)
```

```
cmr3 <- metareg(c, sig)
print(cmr3)
cmr4 <- metareg(c, category)
print(cmr4)
cmr5 <- metareg(c, energy)
print(cmr5)
cmr6 <- metareg(c, SJR)
print(cmr6)
cmr7 <- metareg(c, Year)
print(cmr7)
cmr8 <- metareg(c, energy + category + Latitude)
print(cmr8)
bubble(cmr8)
# Publication Bias - funnel plot:
funnel(c)
# funnel plot simetry tests:
metabias(c, method="rank")
metabias(c, method="linreg")
#FSN:
fsn(yi=z, vi=vi, data=dados, type="Orwin", target=0.01)
fsn(yi=z, vi=vi, data=dados, type="Rosenthal")
# Cumulative Meta-analysis:
metacum(c, pooled="random", sortvar=n)
forest(metacum(c), xlim=c(-1,1))

# Meta-analysis of Occupancy relationships with energy use in hotels

# read ".csv" file:
dados <- read.csv("Occupancy.csv", as.is=TRUE)
# Check data:
dados
# correlation meta-analysis:
c <- metacor (cor=r, n=n, studlab=paste(Authors, Year), data=dados, fixed=FALSE,
random=TRUE, method.tau="DL")
summary(c)
# forest plot of the meta-analysis:
forest(c, xlim=c(-1,1))
# subgroup meta-analysis:
csg1 <- update (c, byvar=sig, print.byvar=FALSE)
summary(csg1)
csg2 <- update (c, byvar=cat, print.byvar=FALSE)
summary(csg2)
csg3 <- update (c, byvar=energy, print.byvar=FALSE)
summary(csg3)
csg4 <- update (c, byvar=method, print.byvar=FALSE)
summary(csg4)
csg5 <- update (c, byvar=sjrabc, print.byvar=FALSE)
summary(csg5)
csg6 <- update (c, byvar=climatez, print.byvar=FALSE)
summary(csg6)
csg7 <- update (c, byvar=continent, print.byvar=FALSE)
summary(csg7)
csg8 <- update (c, byvar=predsource, print.byvar=FALSE)
summary(csg8)
```

```
csg9 <- update (c, byvar=decade, print.byvar=FALSE)
summary(csg9)
forest(csg2, xlim=c(-1,1))
# Meta-regression analyses:
cmr1 <- metareg(c, Latitude)
print(cmr1)
cmr2 <- metareg(c, Longitude)
print(cmr2)
cmr3 <- metareg(c, sig)
print(cmr3)
cmr4 <- metareg(c, category)
print(cmr4)
cmr5 <- metareg(c, energy)
print(cmr5)
cmr6 <- metareg(c, sjr)
print(cmr6)
cmr7 <- metareg(c, Year)
print(cmr7)
cmr8 <- metareg(c, category + Longitude + Year)
print(cmr8)
bubble(cmr8)
# Publication Bias - funnel plot:
funnel(c)
# funnel plot simetry tests:
metabias(c, method="rank")
metabias(c, method="linreg")
#FSN:
fsn(yi=z, vi=vi, data=dados, type="Orwin", target=0.01)
fsn(yi=z, vi=vi, data=dados, type="Rosenthal")
# Cumulative Meta-analysis:
metacum(c, pooled="random", sortvar=n)
forest(metacum(c), xlim=c(-1,1))

# Meta-analysis of Construction year relationships with energy use in hotels

# read ".csv" file:
dados <- read.csv("ConstructionYear.csv", as.is=TRUE)
# Check data:
dados
# correlation meta-analysis:
c <- metacor (cor=r, n=n, studlab=paste(Authors, Year), data=dados, fixed=FALSE,
random=TRUE, method.tau="DL")
summary(c)
# forest plot of the meta-analysis:
forest(c, xlim=c(-1,1))
#FSN:
fsn(yi=z, vi=vi, data=dados, type="Orwin", target=0.01)
fsn(yi=z, vi=vi, data=dados, type="Rosenthal")

# Meta-analysis of Star Level relationships with energy use in hotels

# read ".csv" file:
dados <- read.csv("StarLevel.csv", as.is=TRUE)
# Check data:
```

```
dados
# correlation meta-analysis:
c <- metacor (cor=r, n=n, studlab=paste(Authors, Year), data=dados, fixed=FALSE,
random=TRUE, method.tau="DL")
summary(c)
# forest plot of the meta-analysis:
forest(c, xlim=c(-1,1))
#FSN:
fsn(yi=z, vi=vi, data=dados, type="Orwin", target=0.01)
fsn(yi=z, vi=vi, data=dados, type="Rosenthal")

# Meta-analysis of Number of Floors relationships with energy use in hotels

# read ".csv" file:
dados <- read.csv("NumberFloors.csv", as.is=TRUE)
# Check data:
dados
# correlation meta-analysis:
c <- metacor (cor=r, n=n, studlab=paste(Authors, Year), data=dados, fixed=FALSE,
random=TRUE, method.tau="DL")
summary(c)
# forest plot of the meta-analysis:
forest(c, xlim=c(-1,1))
#FSN:
fsn(yi=z, vi=vi, data=dados, type="Orwin", target=0.01)
fsn(yi=z, vi=vi, data=dados, type="Rosenthal")

# Meta-analysis of Building Age relationships with energy use in hotels

# read ".csv" file:
dados <- read.csv("BuildingAge.csv", as.is=TRUE)
# Check data:
dados
# correlation meta-analysis:
c <- metacor (cor=r, n=n, studlab=paste(Authors, Year), data=dados, fixed=FALSE,
random=TRUE, method.tau="DL")
summary(c)
# forest plot of the meta-analysis:
forest(c, xlim=c(-1,1))
#FSN:
fsn(yi=z, vi=vi, data=dados, type="Orwin", target=0.01)
fsn(yi=z, vi=vi, data=dados, type="Rosenthal")

# Meta-analysis of Guestrooms Area relationships with energy use in hotels

# read ".csv" file:
dados <- read.csv("GuestroomsArea.csv", as.is=TRUE)
# Check data:
dados
# correlation meta-analysis:
c <- metacor (cor=r, n=n, studlab=paste(Authors, Year), data=dados, fixed=FALSE,
random=TRUE, method.tau="DL")
summary(c)
# forest plot of the meta-analysis:
```

```
forest(c, xlim=c(-1,1))
#FSN:
fsn(yi=z, vi=vi, data=dados, type="Orwin", target=0.01)
fsn(yi=z, vi=vi, data=dados, type="Rosenthal")

# Meta-analysis of Retrofit relationships with energy use in hotels

# read ".csv" file:
dados <- read.csv("Retrofit.csv", as.is=TRUE)
# Check data:
dados
# correlation meta-analysis:
c <- metacor (cor=r, n=n, studlab=paste(Authors, Year), data=dados, fixed=FALSE,
random=TRUE, method.tau="DL")
summary(c)
# forest plot of the meta-analysis:
forest(c, xlim=c(-1,1))
#FSN:
fsn(yi=z, vi=vi, data=dados, type="Orwin", target=0.01)
fsn(yi=z, vi=vi, data=dados, type="Rosenthal")

# Meta-analysis of Guest-nights relationships with energy use in hotels

# read ".csv" file:
dados <- read.csv("GuestNights.csv", as.is=TRUE)
# Check data:
dados
# correlation meta-analysis:
c <- metacor (cor=r, n=n, studlab=paste(Authors, Year), data=dados, fixed=FALSE,
random=TRUE, method.tau="DL")
summary(c)
# forest plot of the meta-analysis:
forest(c, xlim=c(-1,1))
#FSN:
fsn(yi=z, vi=vi, data=dados, type="Orwin", target=0.01)
fsn(yi=z, vi=vi, data=dados, type="Rosenthal")

# Meta-analysis of Number of Guests relationships with energy use in hotels

# read ".csv" file:
dados <- read.csv("NumberGuests.csv", as.is=TRUE)
# Check data:
dados
# correlation meta-analysis:
c <- metacor (cor=r, n=n, studlab=paste(Authors, Year), data=dados, fixed=FALSE,
random=TRUE, method.tau="DL")
summary(c)
# forest plot of the meta-analysis:
forest(c, xlim=c(-1,1))
#FSN:
fsn(yi=z, vi=vi, data=dados, type="Orwin", target=0.01)
fsn(yi=z, vi=vi, data=dados, type="Rosenthal")

# Meta-analysis of Food Covers relationships with energy use in hotels
```

```
# read ".csv" file:
dados <- read.csv("FoodCovers.csv", as.is=TRUE)
# Check data:
dados
# correlation meta-analysis:
c <- metacor (cor=r, n=n, studlab=paste(Authors, Year), data=dados, fixed=FALSE,
random=TRUE, method.tau="DL")
summary(c)
# forest plot of the meta-analysis:
forest(c, xlim=c(-1,1))
#FSN:
fsn(yi=z, vi=vi, data=dados, type="Orwin", target=0.01)
fsn(yi=z, vi=vi, data=dados, type="Rosenthal")

# Meta-analysis of Room Revenue relationships with energy use in hotels

# read ".csv" file:
dados <- read.csv("RoomRevenue.csv", as.is=TRUE)
# Check data:
dados
# correlation meta-analysis:
c <- metacor (cor=r, n=n, studlab=paste(Authors, Year), data=dados, fixed=FALSE,
random=TRUE, method.tau="DL")
summary(c)
# forest plot of the meta-analysis:
forest(c, xlim=c(-1,1))
#FSN:
fsn(yi=z, vi=vi, data=dados, type="Orwin", target=0.01)
fsn(yi=z, vi=vi, data=dados, type="Rosenthal")

# Meta-analysis of Number of Employees relationships with energy use in hotels

# read ".csv" file:
dados <- read.csv("NumberEmployees.csv", as.is=TRUE)
# Check data:
dados
# correlation meta-analysis:
c <- metacor (cor=r, n=n, studlab=paste(Authors, Year), data=dados, fixed=FALSE,
random=TRUE, method.tau="DL")
summary(c)
# forest plot of the meta-analysis:
forest(c, xlim=c(-1,1))
#FSN:
fsn(yi=z, vi=vi, data=dados, type="Orwin", target=0.01)
fsn(yi=z, vi=vi, data=dados, type="Rosenthal")

# Meta-analysis of Employees Density relationships with energy use in hotels

# read ".csv" file:
dados <- read.csv("EmployeesDensity.csv", as.is=TRUE)
# Check data:
dados
# correlation meta-analysis:
```

```
c <- metacor (cor=r, n=n, studlab=paste(Authors, Year), data=dados, fixed=FALSE,
random=TRUE, method.tau="DL")
summary(c)
# forest plot of the meta-analysis:
forest(c, xlim=c(-1,1))
#FSN:
fsn(yi=z, vi=vi, data=dados, type="Orwin", target=0.01)
fsn(yi=z, vi=vi, data=dados, type="Rosenthal")

# Meta-analysis of Outdoor Temperature relationships with energy use in hotels

# read ".csv" file:
dados <- read.csv("OutdoorTemperature.csv", as.is=TRUE)
# Check data:
dados
# correlation meta-analysis:
c <- metacor (cor=r, n=n, studlab=paste(Authors, Year), data=dados, fixed=FALSE,
random=TRUE, method.tau="DL")
summary(c)
# forest plot of the meta-analysis:
forest(c, xlim=c(-1,1))
#FSN:
fsn(yi=z, vi=vi, data=dados, type="Orwin", target=0.01)
fsn(yi=z, vi=vi, data=dados, type="Rosenthal")

# Meta-analysis of Carbon Emissions relationships with energy use in hotels

# read ".csv" file:
dados <- read.csv("CarbonEmissions.csv", as.is=TRUE)
# Check data:
dados
# correlation meta-analysis:
c <- metacor (cor=r, n=n, studlab=paste(Authors, Year), data=dados, fixed=FALSE,
random=TRUE, method.tau="DL")
summary(c)
# forest plot of the meta-analysis:
forest(c, xlim=c(-1,1))
#FSN:
fsn(yi=z, vi=vi, data=dados, type="Orwin", target=0.01)
fsn(yi=z, vi=vi, data=dados, type="Rosenthal")

# Meta-analysis of Water Use relationships with energy use in hotels

# read ".csv" file:
dados <- read.csv("WaterUse.csv", as.is=TRUE)
# Check data:
dados
# correlation meta-analysis:
c <- metacor (cor=r, n=n, studlab=paste(Authors, Year), data=dados, fixed=FALSE,
random=TRUE, method.tau="DL")
summary(c)
# forest plot of the meta-analysis:
forest(c, xlim=c(-1,1))
#FSN:
```

```
fsn(yi=z, vi=vi, data=dados, type="Orwin", target=0.01)
fsn(yi=z, vi=vi, data=dados, type="Rosenthal")
```
