# Supplementary material for: Energy use and its contributors in hotel buildings: A systematic review and meta-analysis
Source: PLoS One. 2024 Oct 24;19(10):e0309745. doi: 10.1371/journal.pone.0309745 (PMC11500959; doi:10.1371/journal.pone.0309745)

S5 Fig. Forest plots for all meta-analyses

1. Floor Area

Study	Total	COR	95%-CI	Weight
Chan and Lam (1) 2002	17	0.94	[0.83; 0.98]	3.1%
Chan and Lam (1b) 2002	17	-0.37	[-0.72; 0.13]	3.1%
Chan and Lam (2) 2002	11	0.92	[0.71; 0.98]	2.8%
Chan and Lam (2b) 2002	11	-0.64	[-0.89; -0.06]	2.8%
Deng 2003	30	0.81	[0.64; 0.91]	3.4%
Chan and Mak 2004	10	0.95	[0.80; 0.99]	2.7%
Alujevic (1) 2006	57	0.68	[0.51; 0.80]	3.5%
Alujevic (2) 2006	32	0.60	[0.32; 0.78]	3.4%
Alujevic (3) 2006	57	0.09	[-0.17; 0.35]	3.5%
Alujevic (4) 2006	32	0.10	[-0.26; 0.43]	3.4%
Bohdanowicz and Martinac (1) 2007	73	0.84	[0.76; 0.90]	3.6%
Bohdanowicz and Martinac (2) 2007	111	0.87	[0.82; 0.91]	3.6%
Bohdanowicz and Martinac (3) 2007	73	0.30	[0.08; 0.50]	3.6%
Bohdanowicz and Martinac (4) 2007	111	0.17	[-0.02; 0.35]	3.6%
Priyadarsini et.al.(1) 2009	29	0.95	[0.90; 0.98]	3.4%
Priyadarsini et.al.(2) 2009	29	0.16	[-0.21; 0.50]	3.4%
Wang 2012	200	0.92	[0.90; 0.94]	3.7%
Wang(2) 2012	200	0.16	[0.03; 0.30]	3.7%
Kong et. al. 2012	26	0.76	[0.52; 0.88]	3.3%
Lai 2016	30	0.55	[0.24; 0.76]	3.4%
Lai(2) 2016	30	-0.15	[-0.48; 0.22]	3.4%
Oluseyi et. al. 2016	28	0.77	[0.55; 0.89]	3.4%
Oluseyi et. al.(2) 2016	28	-0.07	[-0.43; 0.31]	3.4%
Tang et al. 2016	24	0.44	[0.04; 0.72]	3.3%
Tangon et al. 2018	63	0.82	[0.72; 0.89]	3.6%
Nguyen and Rockwood (1) 2019	21	0.80	[0.56; 0.92]	3.3%
Nguyen and Rockwood (2) 2019	45	0.88	[0.80; 0.93]	3.5%
Santiago 2021	6	0.85	[0.14; 0.98]	2.0%
Stimac et. al. (1) 2023	51	0.85	[0.75; 0.91]	3.5%
Stimac et. al. (2) 2023	51	0.58	[0.36; 0.74]	3.5%
**Random effects model**	**1503**	**0.65**	**[0.49; 0.76]**	**100.0%**

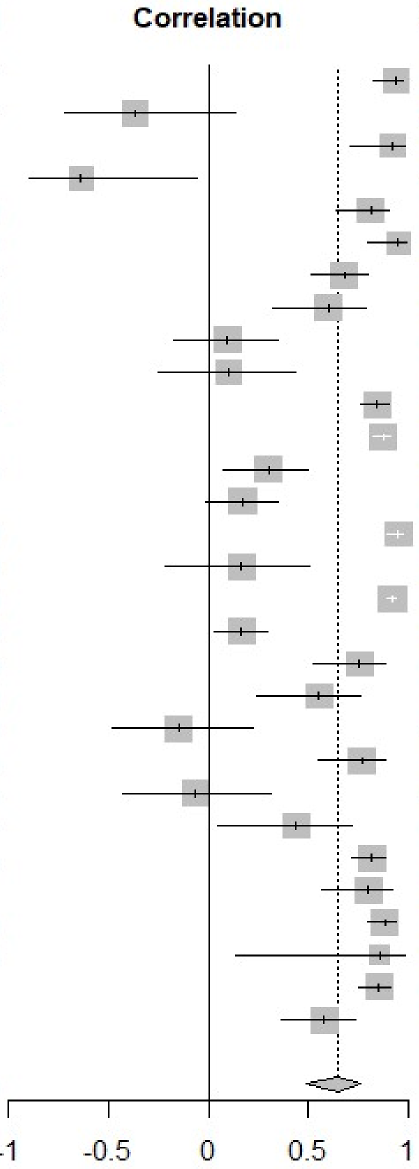

Heterogeneity: $I^2 = 95\%$, $\tau^2 = 0.3852$, $p < 0.01$

2. Guestrooms

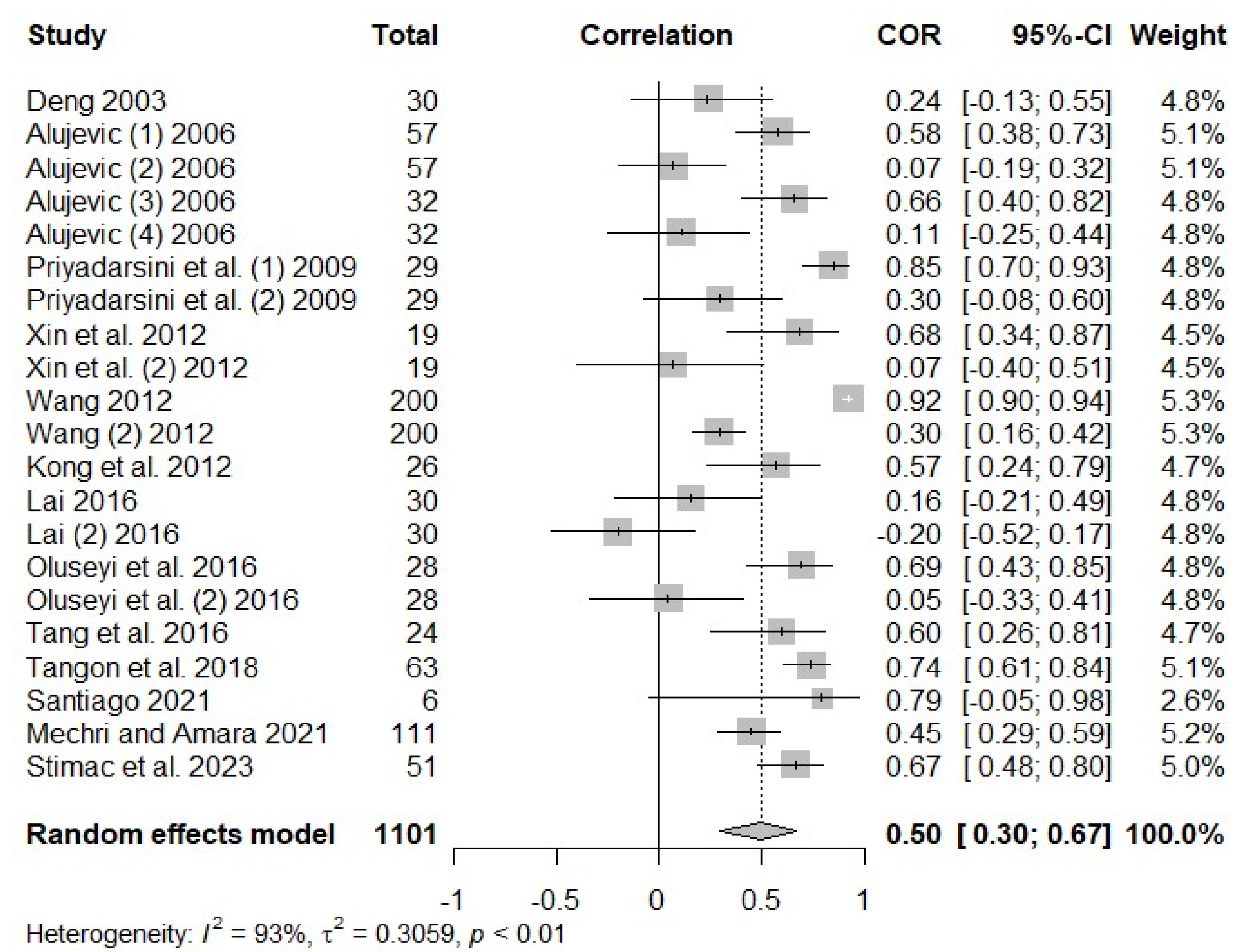

3. Construction Year

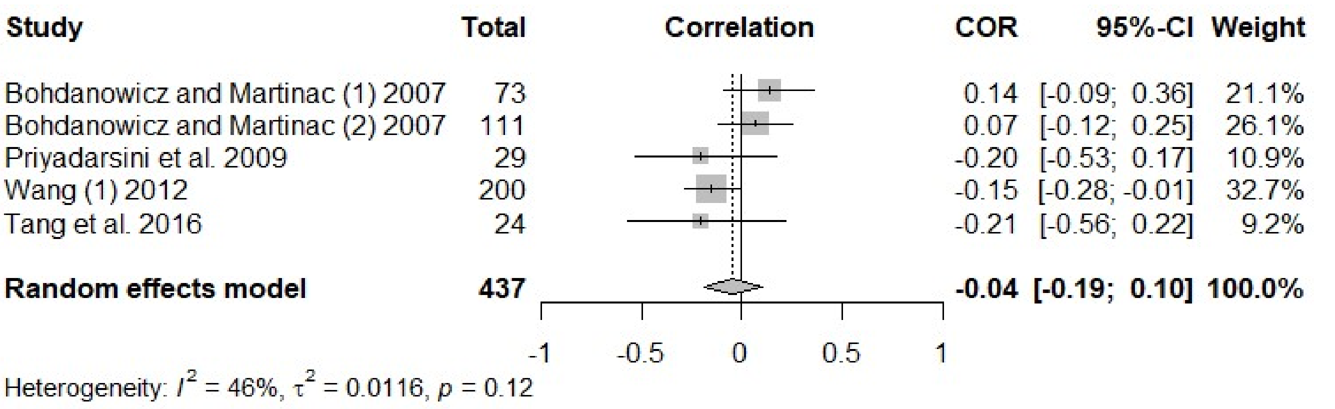

4. Star Level

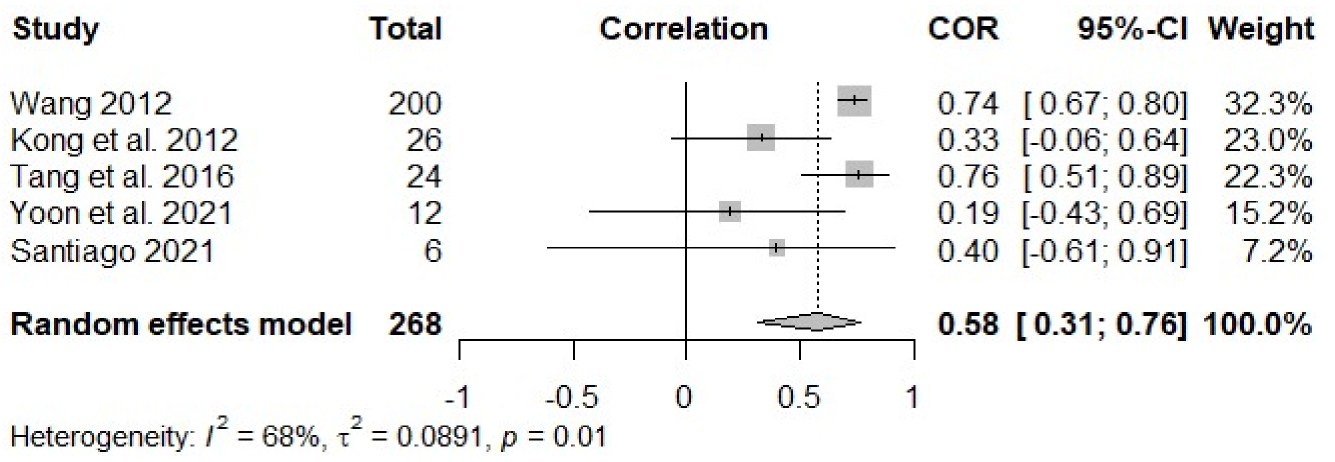

5. Number of Floors

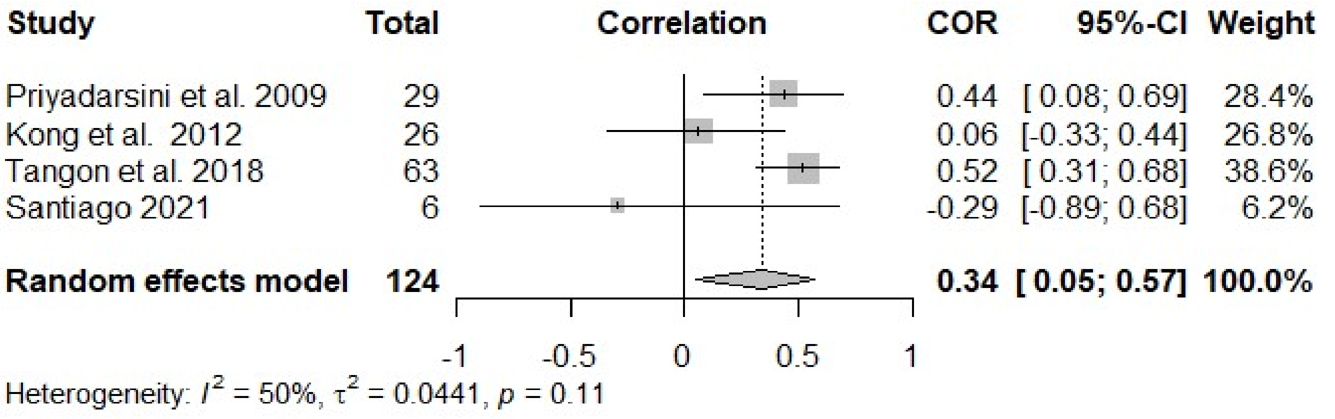

6. Building Age

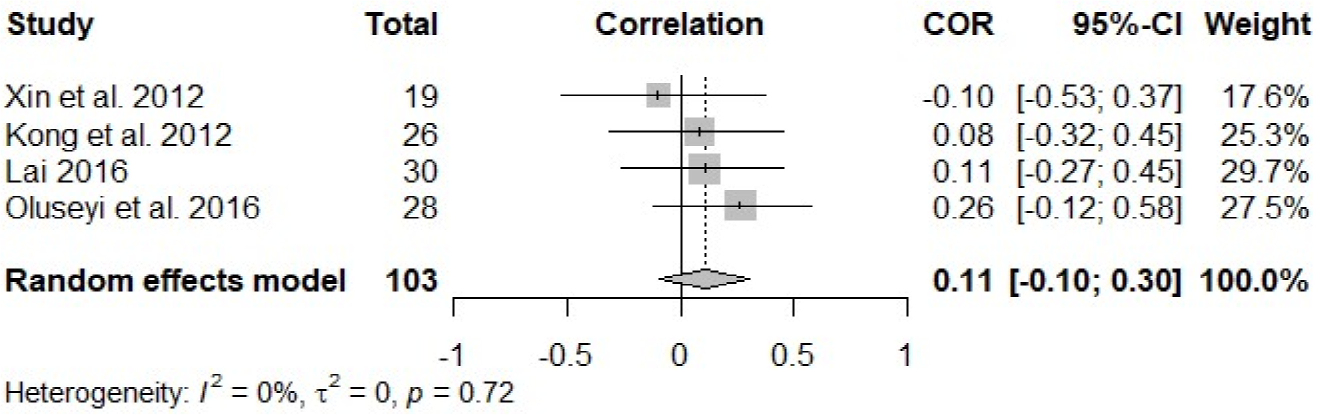

7. Guestrooms Area

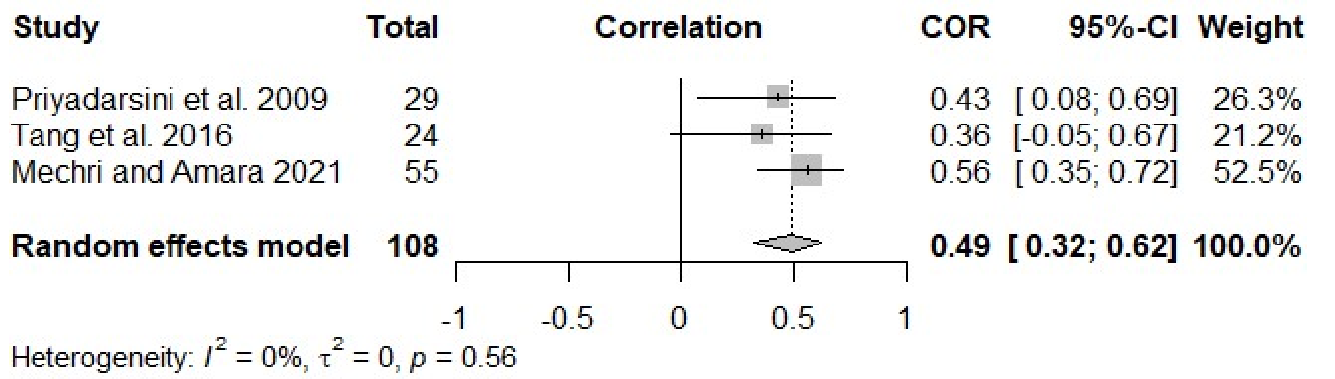

8. Retrofit

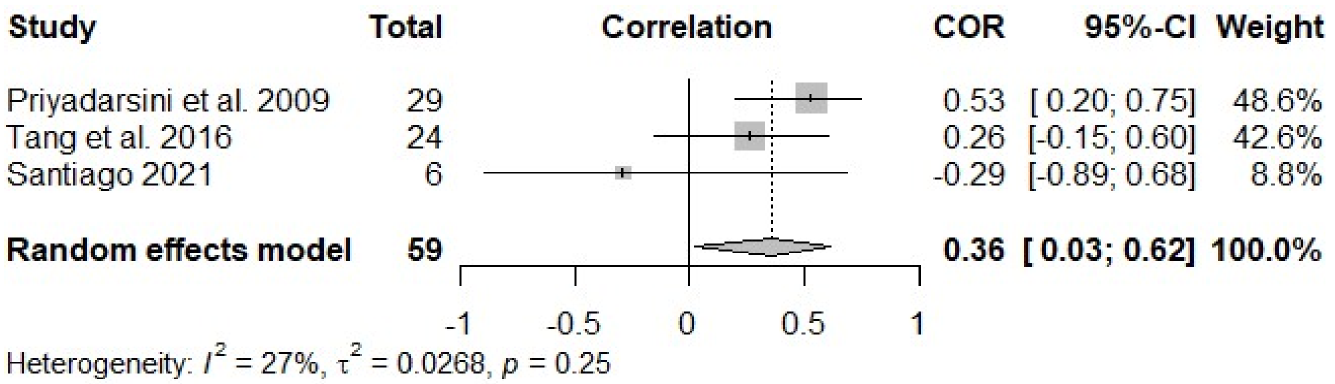

9. Occupancy

Study	Total	COR	95%-CI	Weight
Chan and Lam (1) 2002	11	0.52	[-0.12; 0.85]	3.9%
Chan and Lam (2) 2002	17	0.78	[0.47; 0.92]	4.6%
Alujevic 2006	14	0.12	[-0.44; 0.61]	4.3%
Priyadarsini et al. (1) 2009	29	0.76	[0.55; 0.88]	5.1%
Priyadarsini et al. (2) 2009	29	0.25	[-0.12; 0.57]	5.1%
Xin et al. 2012	19	0.03	[-0.43; 0.48]	4.7%
Xin et al. (2) 2012	19	0.36	[-0.11; 0.70]	4.7%
Wang 2012	200	0.64	[0.55; 0.71]	5.8%
Wang (2) 2012	200	0.71	[0.64; 0.77]	5.8%
Kong et al. 2012	26	0.03	[-0.36; 0.41]	5.0%
Lai 2016	30	-0.39	[-0.66; -0.03]	5.1%
Lai (2) 2016	30	-0.37	[-0.65; -0.01]	5.1%
Oluseyi et al. 2016	28	-0.13	[-0.48; 0.26]	5.1%
Oluseyi et al.(2) 2016	28	0.04	[-0.34; 0.41]	5.1%
Perez et al. 2016	6	-0.95	[-0.99; -0.60]	2.5%
Tang et al. 2016	24	0.42	[0.02; 0.71]	5.0%
Tangon et al. 2018	63	-0.25	[-0.47; -0.01]	5.6%
Nguyen and Rockwood 2019	50	0.00	[-0.28; 0.28]	5.5%
Santiago 2021	6	0.39	[-0.62; 0.91]	2.5%
Mechri and Amara 2021	130	0.00	[-0.17; 0.17]	5.8%
Okpala et al. 2023	10	0.54	[-0.14; 0.87]	3.7%
**Random effects model**	**969**	**0.20**	**[-0.03; 0.41]**	**100.0%**

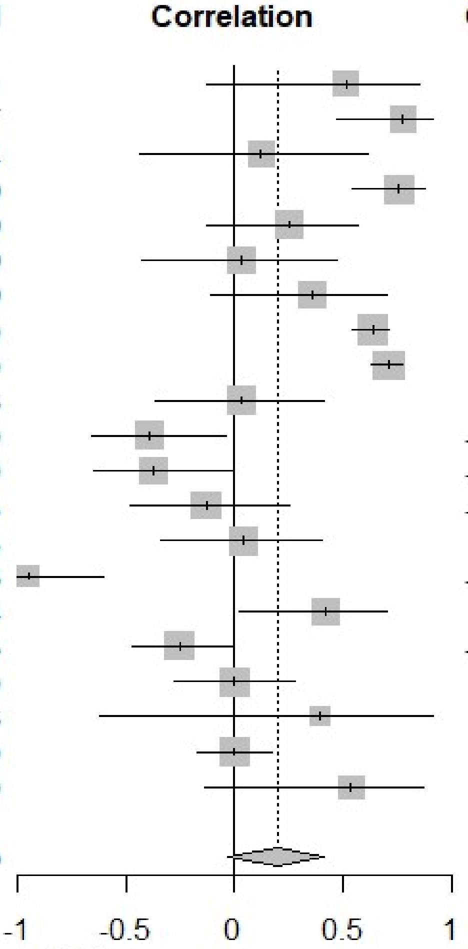

Heterogeneity: $I^2 = 90\%$, $\tau^2 = 0.2384$, $p < 0.01$

10. Guest-nights

Study	Total	COR	95%-CI	Weight
Alujevic 2006	14	0.60	[0.11; 0.86]	9.5%
Bohdanowicz and Martinac (1) 2007	73	0.62	[0.45; 0.74]	15.0%
Bohdanowicz and Martinac (2) 2007	111	0.82	[0.75; 0.87]	15.5%
Priyadarsini et al. 2009	29	0.84	[0.68; 0.92]	12.7%
Oluseyi et al. 2016	28	0.66	[0.38; 0.83]	12.5%
Tangon et al. 2018	63	0.77	[0.65; 0.86]	14.7%
Santiago 2021	6	0.92	[0.43; 0.99]	4.4%
Mechri and Amara 2021	133	0.35	[0.19; 0.49]	15.7%
**Random effects model**	**457**	**0.71**	**[0.54; 0.82]**	**100.0%**

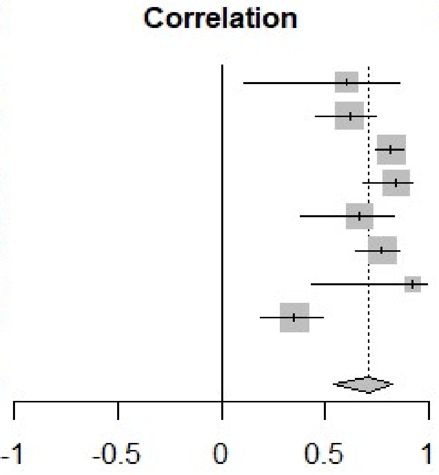

Heterogeneity: $I^2 = 85\%$, $\tau^2 = 0.1188$, $p < 0.01$

11. Number of guests

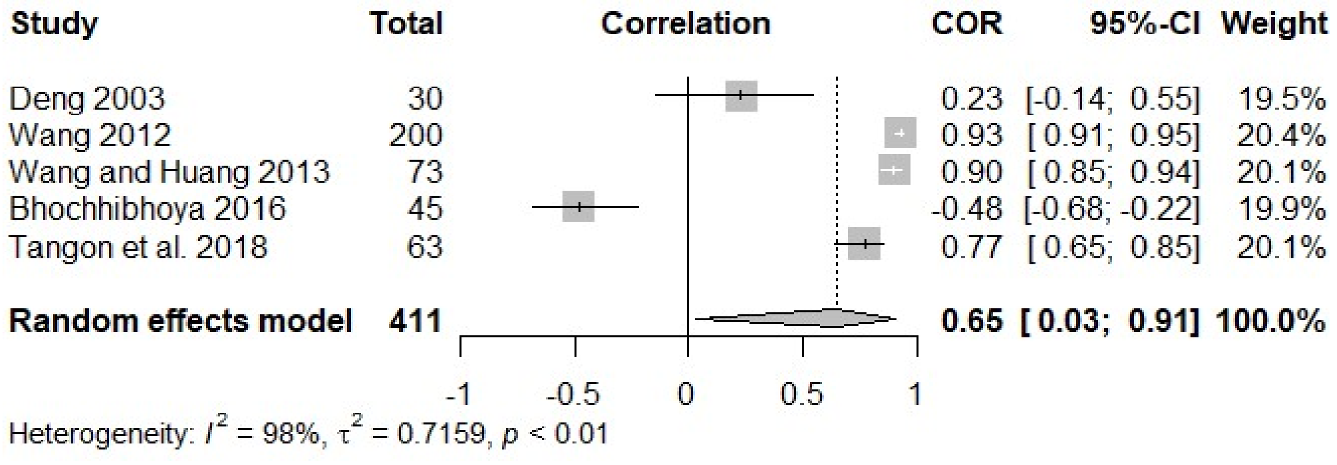

12. Food covers

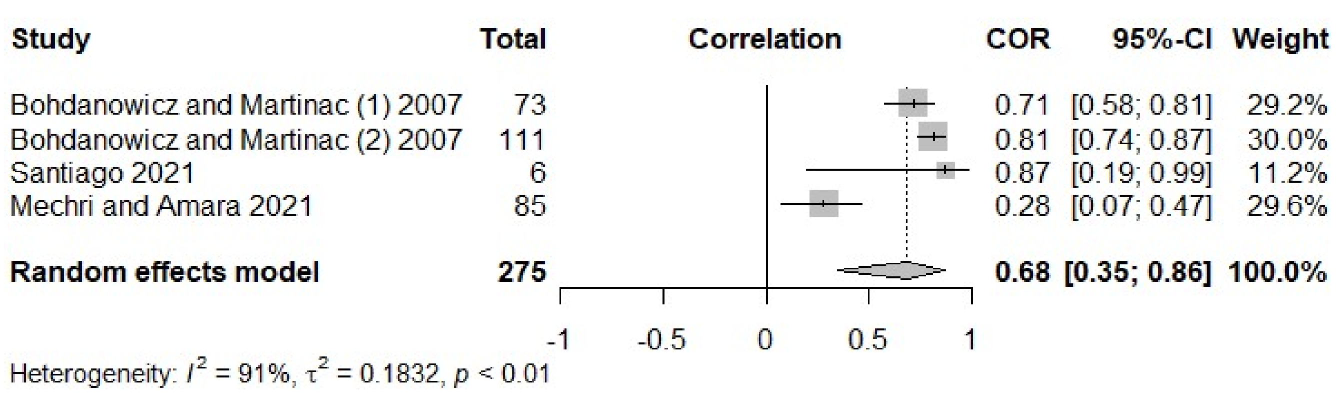

13. Room Revenue

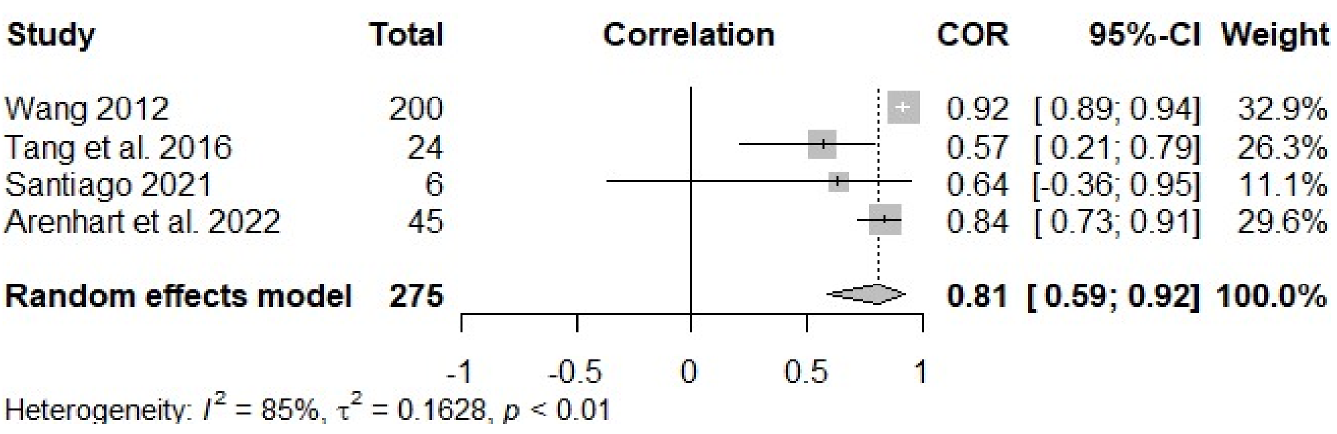

14. Number of Employees

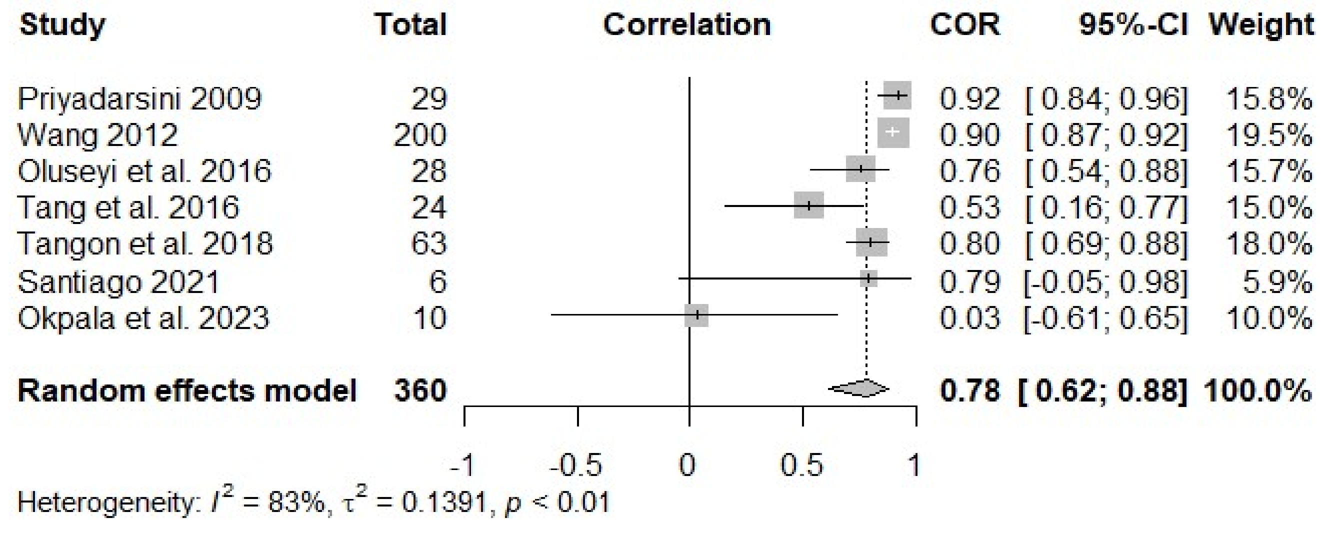

15. Employees Density

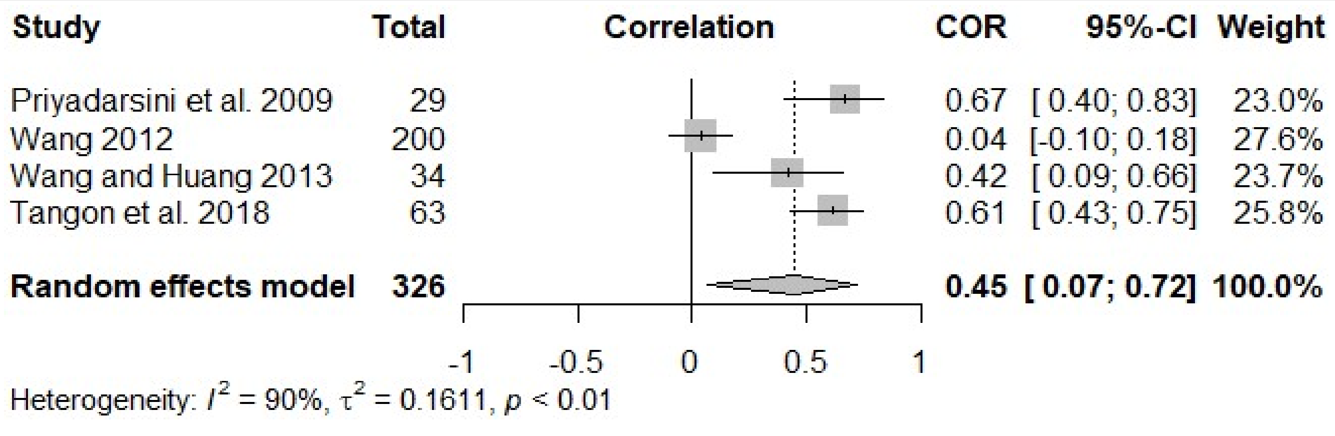

16. Outdoor temperature

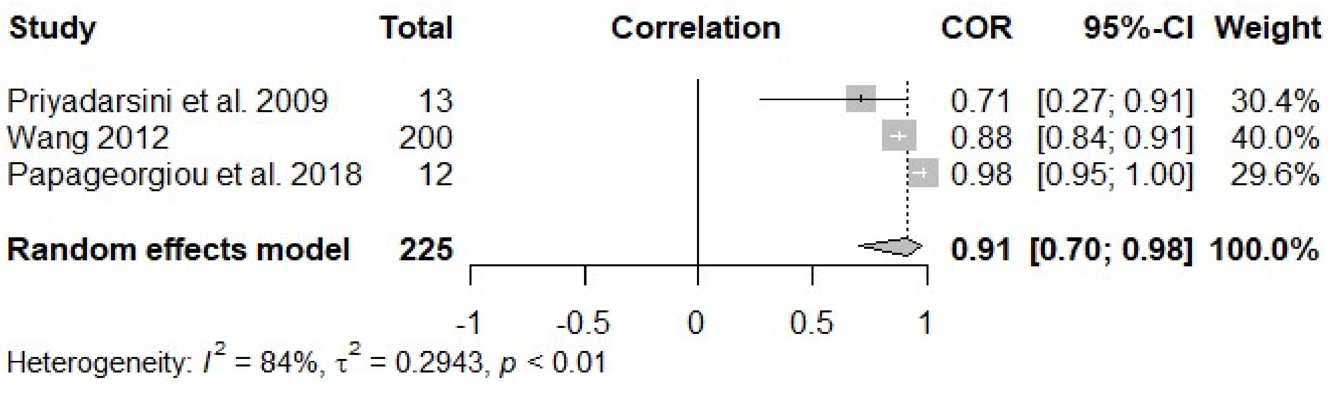

17. Carbon Emissions

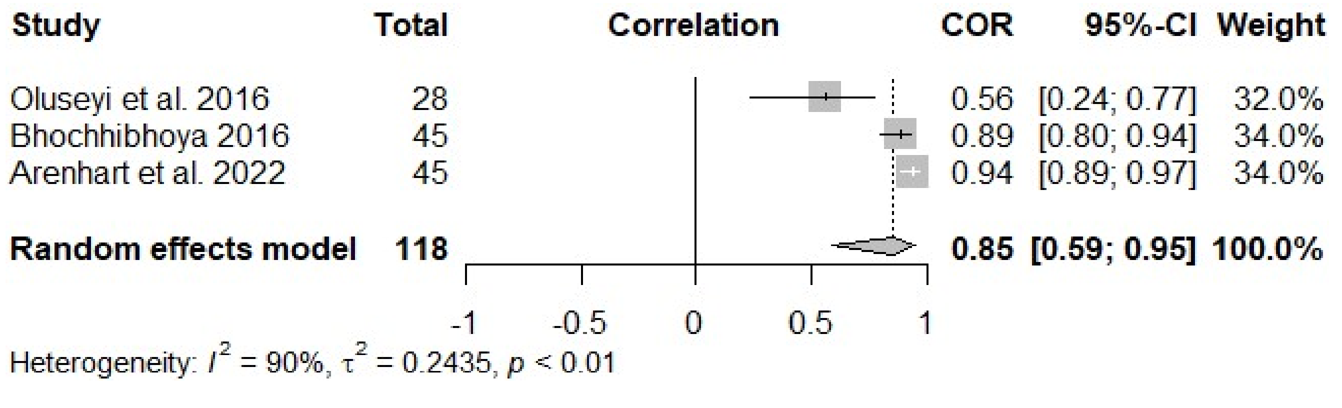

18. Water Use

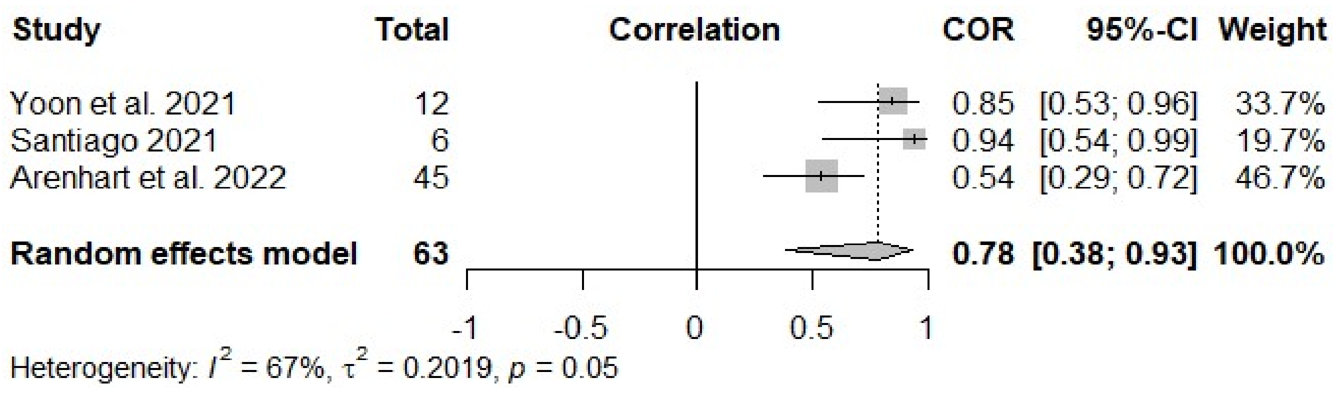

Supplement: S1 Fig — (PDF) [file pone.0309745.s008.pdf]
